# Synthesis, characterization, molecular docking, pharmacokinetics, and molecular dynamics of new *bis*-thiazoles based on *bis*-thiosemicarbazone as anti-coxsackievirus

**DOI:** 10.1038/s41598-024-80753-z

**Published:** 2024-11-26

**Authors:** Thoraya A. Farghaly, Eman M. H. Abbas, Heba S. Abd-Elghaffar, Mohamed A. Elsayed, Dina H. Elnaggar, Ahmed F. El-Sayed, Dina N. Abd-Elshafy, Salwa F. Mohamed

**Affiliations:** 1 Department of Chemistry, Faculty of Science, Cairo University, Giza, 12613 Egypt; 2 Department of Chemistry, Natural and Microbial Products, National Research Centre, Dokki, Cairo Egypt; 3 Applied Organic Chemistry Department, National Research Centre, Dokki, 12622 Cairo Egypt; 4 Microbial Genetics Department, Biotechnology Research Institute, National Research Centre, Giza, Egypt; 5 Egypt Center for Research and Regenerative Medicine (ECRRM), Cairo, Egypt; 6 Water Pollution Research Department, National Research Centre, Dokki, 12622 Cairo Egypt; 7 Research Group Immune- and Bio-markers for Infection, Centre of Excellence for Advanced Science, National Research Centre, Dokki, Cairo, 12622 Egypt

**Keywords:** Thiazoles, Haloketones, Molecular docking, ADMET, Dynamic simulations, Coxsackievirus B, Synthetic biology, Chemical biology, Molecular biology

## Abstract

It was known that the majority of viral infections start off as cutaneous eruptions, which heal on their own in most cases. The prognosis is dependent on the state of immunologic surveillance, just like in other infectious disorders. Therefore, those who are immunosuppressed are more in danger. But recently it’s becoming increasingly clear that eruptions that were once thought to be benign diseases can really cause problems and even death, even in immunocompetent patients. Hence, in this article, our goal was to identify possible potential antiviral candidates. We have synthesized a series of *bis*-thiazole derivatives *via* the reaction of *bis*-thiosemicarbazone derivative **3** with hydrazonoyl chlorides and haloketones in an effort to examine their potential antiviral properties and interactions with the main protease of Coxsackievirus B. Spectroscopic methods and elemental analysis were used to corroborate the structures of the novel *bis*-thiazole derivatives. The most potent derivative, *bis*-thiazole derivative **7a**, was found to have the strongest antiviral activity against Coxsackievirus B (Cox B). Further investigation into its mode of action indicated that compound **7a** has a dual activity that inhibits viral adsorption and replication. The efficacy of many compounds against Coxsackievirus adenovirus targets was assessed using molecular docking. The findings revealed that compounds **7a**, **7c**, **11b** and **11c** have high binding energies, efficiently engaging the active sites of essential Cox B virus proteins such as the Coxsackievirus adenovirus receptor (CAR), 3C-protease, and RNA-dependent RNA polymerase (RdRp). These interactions involved a variety of chemical bonding types, indicating that these substances can inhibit enzyme activity while also exhibiting substantial antiviral effects involving viral replication and adsorption. Furthermore, the computational ADMET study of these compounds indicated conformance to Lipinski’s criteria, implying positive physicochemical properties. Furthermore, MD simulations demonstrated stable complexes of **7a** and **11b** with Coxsackievirus adenovirus receptor (CAR), 3C-protease, and RNA dependent RNA polymerase (RdRp) with RMSD (0.1–0.30, 0.20–0.30, and 0.20–0.35 nm), RMSF (0.1–0.5 nm), and SASA (80–105, 140–150, and 220–235). These outcomes further reinforce the potential of these compounds in current antiviral drug development endeavors. The collective findings underscore the potential of these compounds as candidates for antiviral therapies against Coxsackievirus adenovirus.

**Supplementary Information:**

The online version contains supplementary material available at 10.1038/s41598-024-80753-z.

## Introduction

**Fig. 1:**
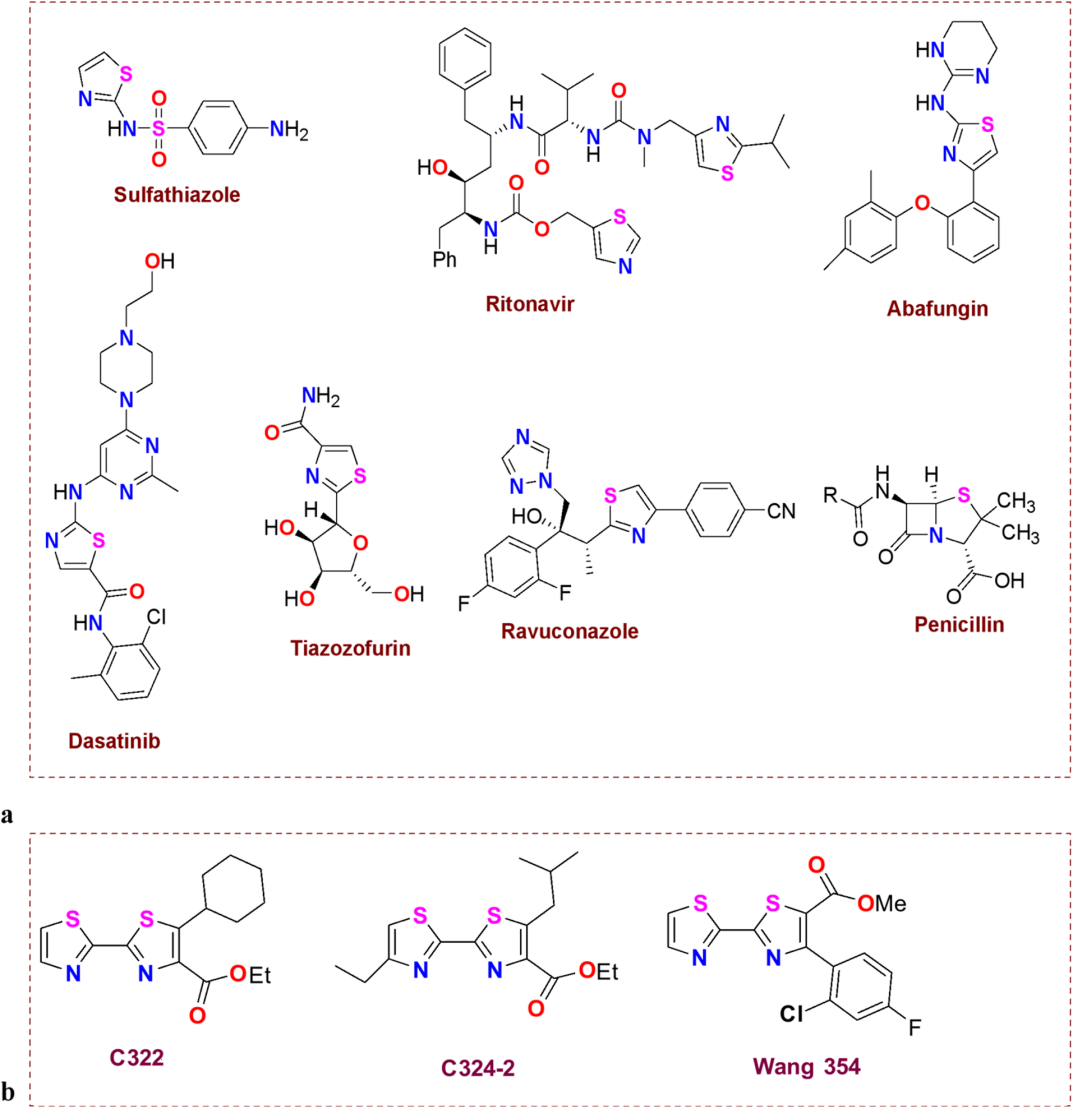
**(a)** Thiazole skeleton containing FDA-approved drugs. **(b)** Some patents antiviral agents containing *bis*-thiazole.

Viral infections can range in severity from mild to life-threatening; some, including HIV, Hepatitis B, and Hepatitis C, are extremely serious problems for global public health^1^. Coxsackieviruses B (Cox B) are known as RNA viruses that belong to the family Picornaviridae. They are transmitted *via *the fecal-oral route, and upon infection, fever, headache, sore throat, gastrointestinal distress, extreme fatigue, as well as chest and muscle pain, occur^2^. More than 90% of the cases don’t develop life-threatening symptoms, but on reaching the pancreas, liver, myocardium, and meninges *via *the bloodstream, they can cause pancreatitis, hepatitis, aseptic meningitis, and myocarditis^3^. Due to a lack of anti-Cox B drugs^4^, antiviral activity of different plant extracts and synthetic compounds was tested as shown to affect the virus, as were the anti-cox B activity of Rheum palmatum^5^, quinoxaline derivatives^6^, and ginsenosides^7^. Focusing on the heterocyclic compounds, we noted that thiazole-containing compounds showed several pharmaceutical applications^8–11^. It is spread in natural products like vitamins (thiamine), peptides^12^, epothilone^13^, chlorophyll^14^, and alkaloids^15^. A long range of biological activities are attributed to the presence of a thiazole ring in the molecules as analgesic^16^, antioxidant^17^, antiallergic^18^, antibacterial^19^, anti-inflammatory^20^, anticancer^21^, antimalarial^22^, and antihypertensive^23^. The Food and Drug Administration (FDA) approved more than eighteen drugs containing thiazole moieties, with the Ritonavir drug containing a *bis*-thiazole moiety in its skeleton, as illustrated in Fig. 1a. Our recently published review article^24^ on the patents antiviral activity of thiazole ring revealed that more than 141 compounds having thiazole ring showed antiviral activity as anti-influenza (H1N1 and H3N2), anti-coronaviruses, anti-HCV, anti-HIV, and anti-HSV-I.

In addition, several studies^25–27^ demonstrated that compounds with more than one thiazole ring unit have good biological activities. Numerous patents in Fig. 1b list *bis*-thiazole compounds as effective antiviral agents^28^. From all the above data and in continuation of our research field in the synthesis of bioactive heterocyclic compounds^29–36^ herein, we synthesized a series of *bis*-thiazole derivatives to investigate their antiviral activity and mechanism of action against Cox B. In addition, the molecular docking, pharmacokinetics ADMET Analysis, and MD stimulation were studied and discussed.

## Results and discussion

### Chemistry

**Scheme 1:**
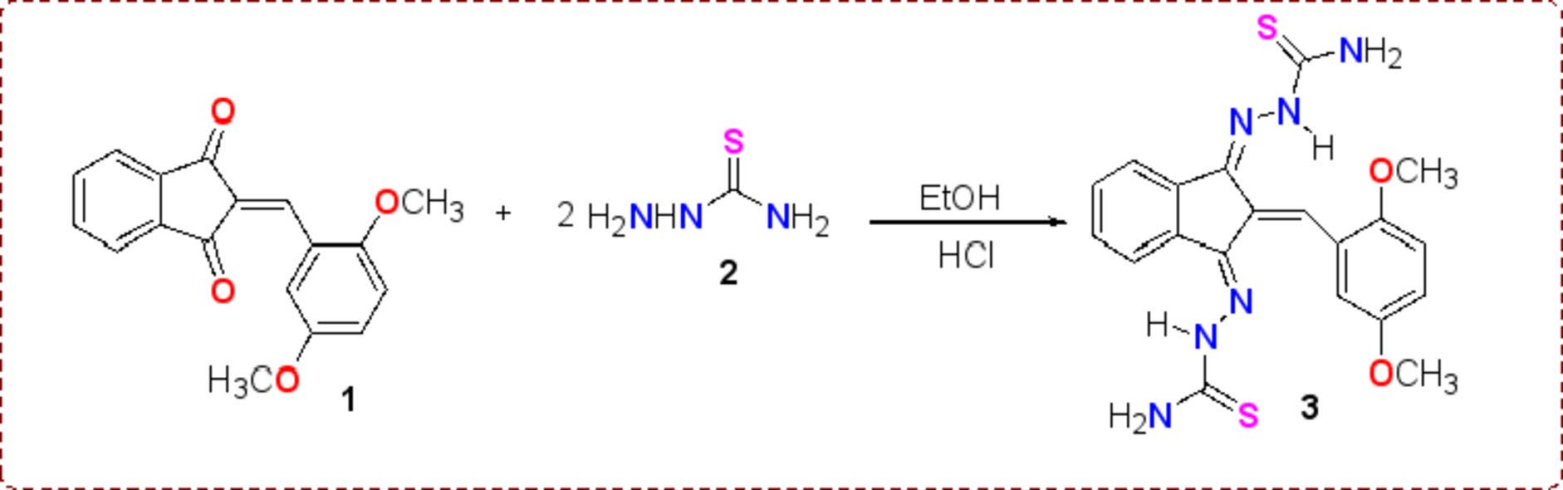
Synthesis of *bis*-thiosemicarbazone derivative **3**.

The condensation reaction of 2-(2,5-dimethoxy-benzylidene)indan-1,3-dione **1**^37^ with two mole equivalents of thiosemicarbazide **2** in ethanol with drops of HCl gave *bis*-thiosemicarbazone **3** (Scheme 1). The structure of *bis*-thiosemicarbazone derivative **3 **was established based on their ^1^H NMR, ^13^C NMR, and IR spectra. As for instance,  ^1^H NMR spectrum of *bis*-thiosemicarbazone derivative **3** revealed six singlet signals at δ = 3.67, 3.73 (two OCH_3_), 8.05, 8.15, 11.38 (three NH), and 8.35 (= CH), in addition to multiple signals in the aromatic region for seven aromatic protons at δ = 6.89–7.60 ppm. The three NH signals in ^1^H NMR of compound **3** are attributed to the non-equivalent two protons of NH_2_ due to one of these two protons forming an H-bond with the C = N as illustrated in Figure S1in the supplementary data Section^24^. In addition, the ^13^C NMR spectrum exhibited a signal at 178.3 ppm, which was ascribed to the carbons of the two thioxo groups having overlapping. The mass of compound **3** was equivalent to the computed values.

**Scheme 2:**
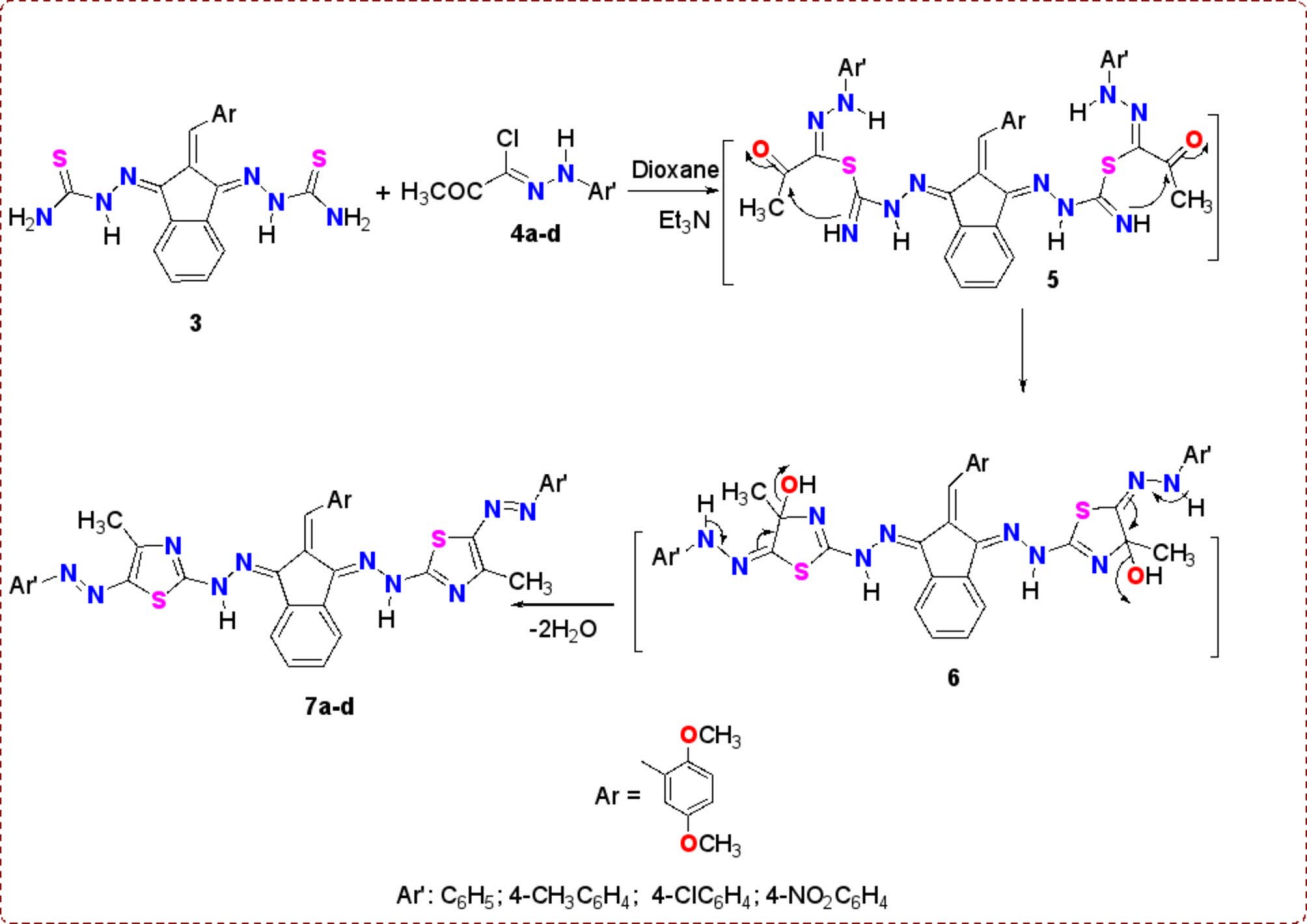
Synthesis of *bis*-thiazole derivatives **7a-d**.

*Bis*-thiosemicarbazone derivative **3** was used as a basic compound for the building of *bis*-thiazole derivatives. So firstly compound **3** (one mole equivalent) was reacted smoothly with hydrazonoyl chloride **4a-d** (two mole equivalents) in dioxane/Et_3_N (the progress of the reactions monitored with TLC). Such reaction proceeded through the formation of two non-isolable intermediates **5** & **6**, followed by the elimination of two water molecules to afford the target *bis*-thiazole derivatives **7a-d** (Scheme 2). ^1^H NMR of all *bis*-thiazole derivatives **7a-d** revealed the characteristic singlet signals of 2CH_3_, 2OCH_3_, =CH, and 2NH protons at δ = 2.27–2.56, 3.71–3.80, 7.41–8.14, and 8.67–8.75 ppm in addition to the other aromatic and aliphatic protons for each derivative. All IR spectra have an absorption band for the NH at ν = 3434 –3394 cm^−1^. ^13^C NMR showed the two methoxy group carbons in derivatives **7a-d** as two signals at roughly 56.0 and 56.8 ppm. Additionally, the products **7a-d**’s molecular weight measurement was found in the anticipated range.

In a similar way and under the same reaction conditions, *bis*-thiosemicarbazone derivative **3** was reacted with α-haloketones **8a**,** b**, **10a-c**, and **12** to afford another series of various *bis*-thiazole derivatives **9a**,** b**, **11a-c**, and **13**, respectively. The structures of all *bis*-thiazole derivatives **9a**,** b**, **11a-c** and **13** were assured based on their spectroscopic data, For example, the IR spectrum of derivative **9b** showed the characteristic absorption bands for C = O and C = N at ν = 1723 and 1638 cm^−1^, respectively. While in its ^1^H NMR spectrum, a doublet signal was observed for 2CH_3_at 1.12 ppm, a quartet signal for two CH-thiazole at 3.97 ppm, and 2NH protons at δ = 11.22 ppm. The signal detected in the ^13^C NMR spectra at 16.7 ppm was attributed to the overlapping carbons of the two methyl groups. Additionally, Compound **11a**’s IR spectrum, revealed the presence of a distinctive absorption band at 3439 cm^−1^, which is linked to the NH group. At 1635 and 1574 cm^−1^, respectively, two medium bands formed that corresponded to the symmetric and asymmetric stretching vibrations of C = N. The creation of compound **11a** was confirmed by the development of a single band for C = O stretching vibrations approximately 1702 cm^−1^, and the presence of a band for the NH group. Furthermore, the target compounds’ effective synthesis was confirmed by the ^1^H NMR spectra. Accordingly, the two singlet peaks of methyl protons at δ 1.78 and 2.30 ppm with six protons integration for each signal and the existence of a singlet signal for NH at δ 11.18 confirmed the synthesis of compound **11a**. While ^13^C NMR provided a signal for the carbonyl carbon at 168.2 ppm (overlapped). For compound **13**, the NH group was detected in the IR spectrum at 3436 cm^−1^. While ^1^H NMR provided multiple signals at 6.94–8.42 ppm for both CH-thiazole with the aromatic protons integrated for twenty protons, and a downfield signal at 11.63 ppm for the two imino protons. Otherwise, MS generated the molecular ion peak corresponding to the confirmed molecular weights of target **13**.

**Scheme 3:**
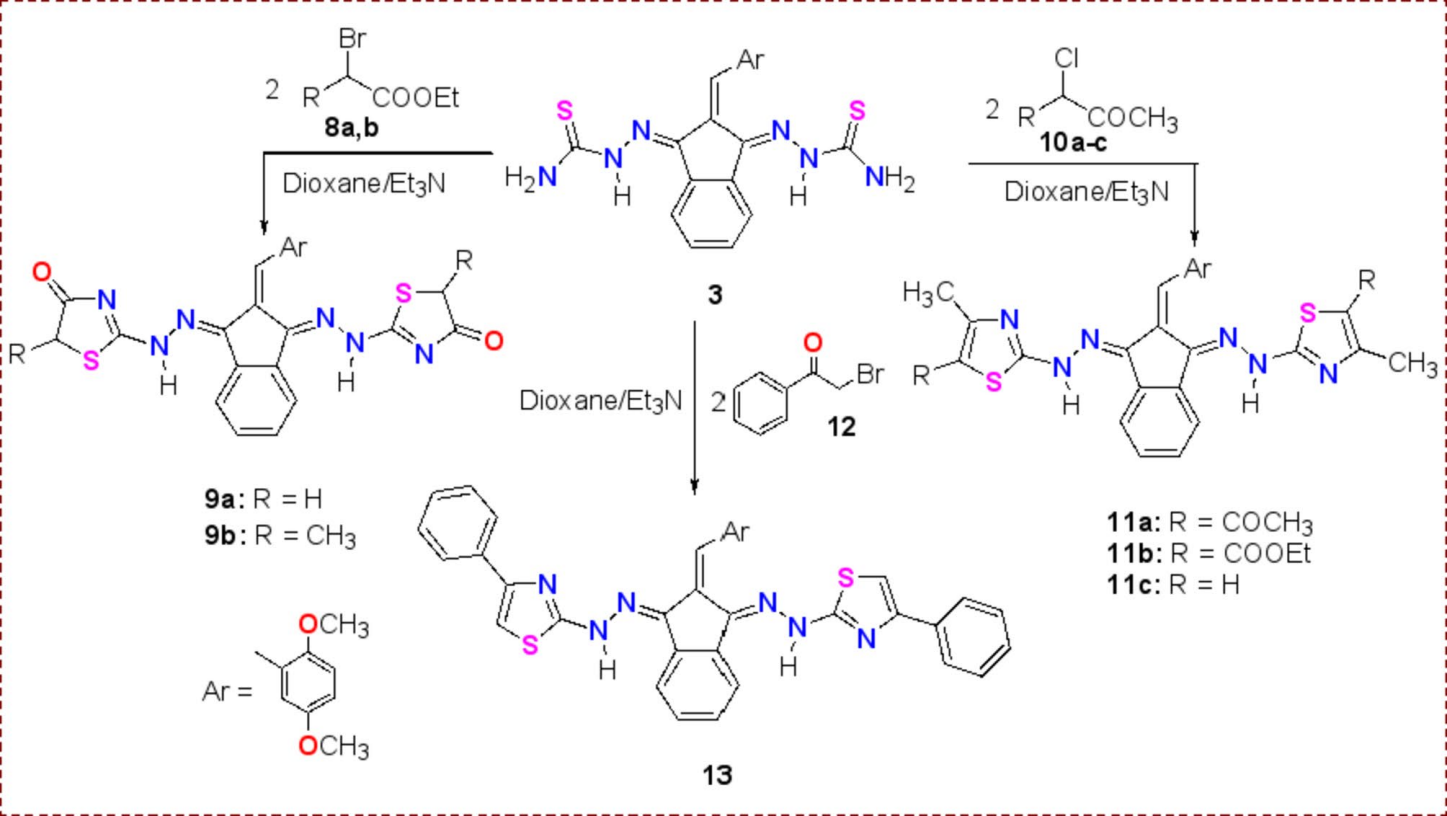
Synthesis of thiazole derivatives **9**, **11** and **13**.

### In vitro antiviral activity

#### Cytotoxicity of tested compounds

Cytotoxicity assay Table 1 shows the safe concentrations of compounds that can be applied to Vero cells without causing any morphological changes after 24 h. Results showed that compounds have different safety effects on cells. Some were highly toxic at low concentration like **9a** and **9b**; others showed higher safety when applied to cells like **1**,** 3**,** 7d**, and **13**. Other compounds were dense and coloured that made masking to the cell sheet, and so it was hard to examine at higher concentrations as compounds **7a**,** 7b**,** 7c**,** 11a**,** 11c**, and **11b**, and accordingly suitable concentrations for each compound were used in the plaque reduction assay.

**Table 1:** Cytotoxicity of tested compounds on Vero cells. Safe concentration + 1, +2, + 3, +4: about 25, 50, 75, 100% of cell sheet was affected respectively, M: masked (color of compounds made cells unseen).

Code	Concentration (µg/100µl)									
	10	20	30	40	50	60	70	80	90	100
**1**	-	-	-	-	-	-	-	-	-	-
**3**	-	-	-	-	-	-	-	-	-	-
**7a**	-	-	-	-	-	+ 1	M	M	M	M
**7b**	-	-	-	-	-	M	M	M	M	M
**7c**	-	-	-	-	-	-	-	-	M	M
**7d**	-	-	-	-	-	-	-	-	-	-
**9a**	-	-	-	-	-	-	+ 1	+ 2	+ 3	+ 4
**9b**	-	-	-	-	-	-	+ 1	+ 1	+ 2	+ 3
**11a**	-	-	+ 1	M	M	M	M	M	M	M
**11b**	-	-	-	-	-	M	M	M	M	M
**11c**	-	-	-	-	-	-	-	-	M	M
**13**	-	-	-	-	-	-	-	-	-	-

#### Antiviral activity of some compounds against Cox B virus

Plaque reduction assay was used to test the antiviral activity of chemical compounds under test at safe concentrations suitable for each compound. Results in Table 2 showed that compound **7a** caused high viral inhibition at tested concentrations.

**Table 2:** Antiviral activity of compounds against Cox B virus. Viral count in control wells (untreated with compounds) was 1.6 × 10^7^, T: toxic.

Code	Concentration (µg/ml)	Viral count (treated)	%Inhibition
**1**	20	1.42 × 10^7^	11
	40	1.6 × 10^7^	0
**3**	20	1.24 × 10^7^	22
	40	0.72 × 10^7^	55
**7a**	20	**0.54 × 10** ^**7**^	**66**
	40	**0 × 10** ^**7**^	**100**
**7b**	20	1.6 × 10^7^	0
	40	1.07 × 10^7^	33
**7c**	20	1.07 × 10^7^	33
	40	1.6 × 10^7^	0
**7d**	20	1.6 × 10^7^	0
	40	0.54 × 10^7^	66
**9a**	20	1.6 × 10^7^	0
	40	1.6 × 10^7^	0
**9b**	20	-	T
	40	-	T
**11a**	20	1.44 × 10^7^	10
	40	0.68 × 10^7^	57
**11b**	20	0.8 × 10^7^	50
	40	0.96 × 10^7^	40
**11c**	20	0.84 × 10^7^	47
	40	0.57 × 10^7^	64
**13**	20	1.6 × 10^7^	0
	40	1.6 × 10^7^	0

#### Mechanism of action of active compounds

**Fig. 2:**
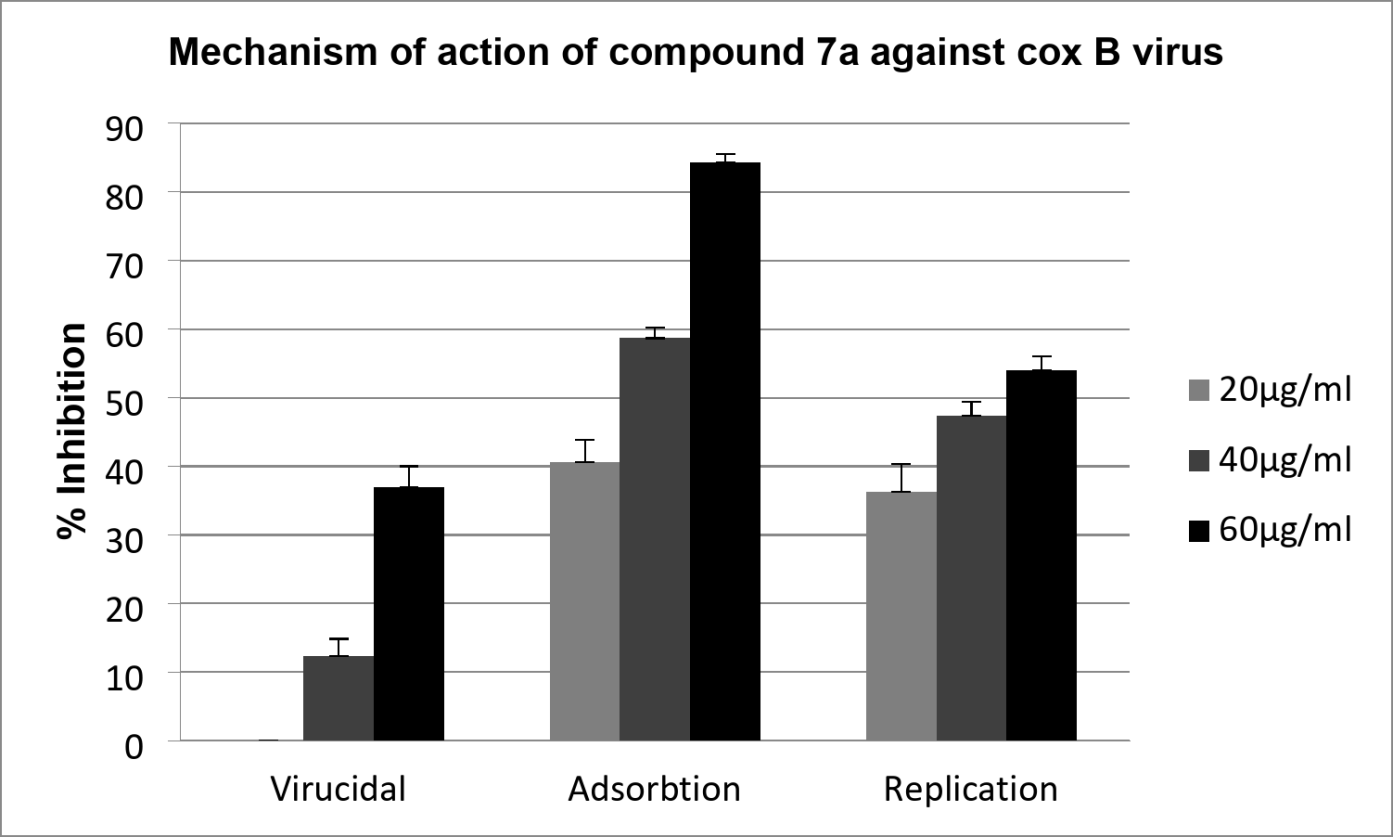
The mechanism of action of bis-thiazole derivative **7a**.

For an active compound having antiviral activity, three possible mechanisms by which it can affect viral infectivity (a) direct effect on the viral particle that we call virucidal; (b) effect on the replicating cycle of the virus, i.e., any of the steps that occur inside the cell supporting viral replication starting from uncoating till assembly and release; (c) effect on the binding site on the cells and thus preventing the virus from being able to enter the target cell and cause infection. On studying the mechanism of action of the most active *bis*-thiazole derivative **7a** (Table 3; Fig. 2), we found that compound **7a** had combined activity between adsorption and replication as it showed higher % inhibition on applying experiment steps that support testing those two mechanisms. Results showed that compound **7a** has a double action, inhibiting both viral adsorption and replication. This means that compound **7a** has the ability to bind to cell receptors of the virus and so prevent viral entry to the target cells, causing an effect on adsorption and also a high % reduction on testing the effect of the compound on replication showed that compound **7a** caused inhibition to one of the important steps needed by the virus to complete its replicating cycle inside the cell.

On the other hand, the tested compound was found to have no direct effect on the viral particle itself, which means that the compound whether didn’t bind with outer surface of the virus or change its configuration and so turned it to be non-infectious.

**Table 3:** The mechanism of action of compound **7a**. Viral count in control wells (untreated with compounds) was 1.6 × 10^7^.

Activity	Concentration (µg/ml)	Viral count (treated)	%Inhibition
Virucidal	20	1.6 × 10^7^	0
	40	1.4 × 10^7^	12
	60	1 × 10^7^	37
Adsorption	20	0.92 × 10^7^	42
	40	0.68 × 10^7^	57
	60	0.24 × 10^7^	85
Replication	20	1 × 10^7^	37
	40	0.8 × 10^7^	48
	60	0.7 × 10^7^	56

### Computational analysis

#### Molecular docking

Molecular docking is commonly used to predict the alignment of small molecule medicinal drugs with their protein targets, as well as the small molecule’s affinity and activity. Docking is an important tool in rational drug design. Given the biological and pharmacological significance of docking investigations, great effort has been made to improve the algorithms for docking prediction^38^. The utility of docking in evaluating the interactions between synthesized compounds and protein receptors. It offers valuable insights into their binding modes and potential biological activities^39–42^.

##### Docking and molecular interaction of synthesized compounds

**Fig. 3:**
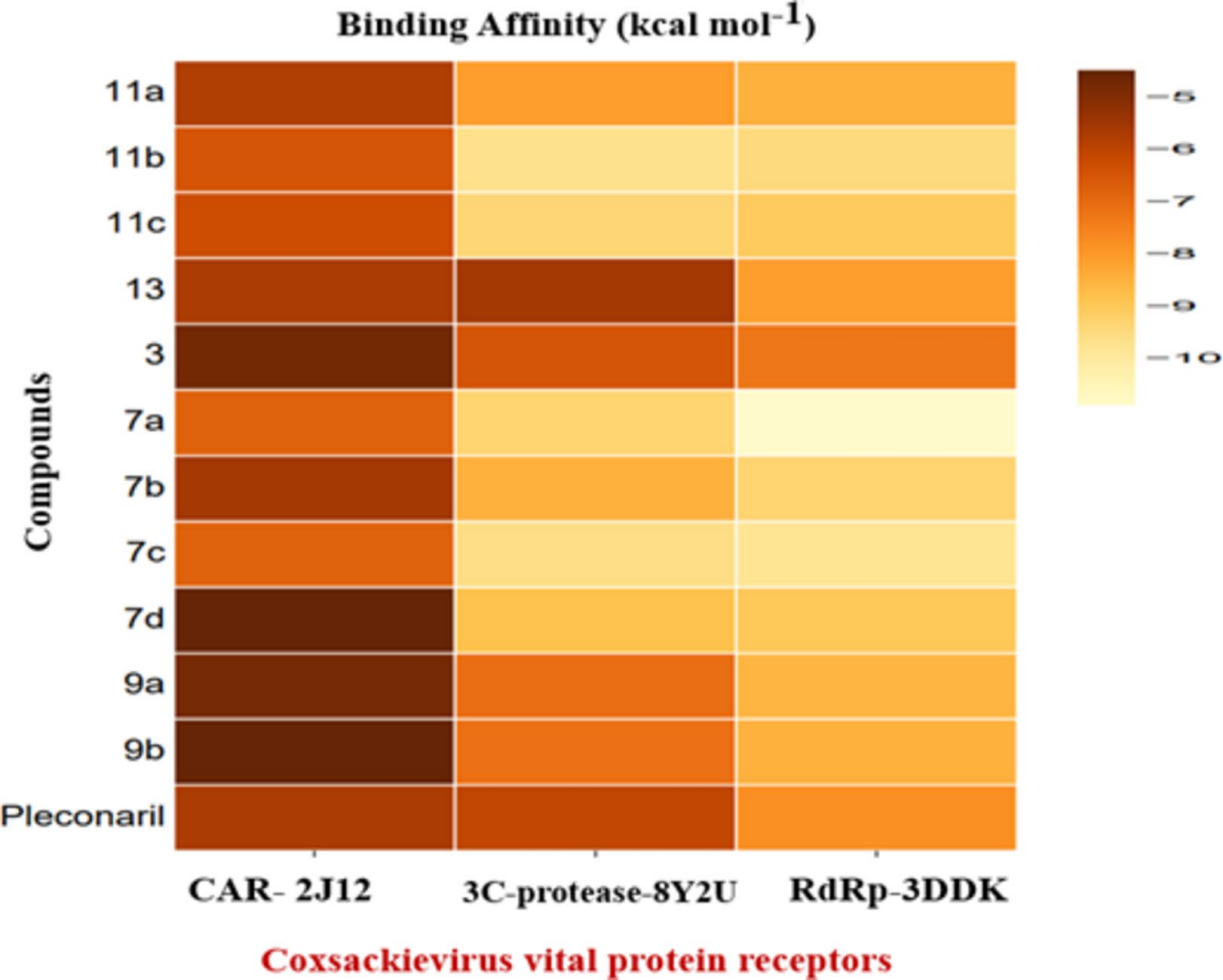
Heat map of binding affinity of compounds with targets of Coxsackievirus vital protein receptors.

Molecular docking was used to study the binding relationships between the generated compounds and protein targets linked to Coxsackievirus crucial protein receptors. The purpose of this analysis was to provide insight into the compounds’ effectiveness. Table 4; Fig. 3 show the findings of the docking investigations that investigated the binding affinities between the compounds and three Coxsackievirus vital protein receptors. Among affinity of compounds with these three proteins, compounds **7a** and **7c** have the highest binding energy with three Coxsackievirus vital protein receptors and comparing with Pleconaril (an antiviral medication, primarily acts against Coxsackievirus B by inhibiting the replication of the virus. Through a search in the Drug Bank Database using Pleconaril, the protein target identified was Capsid protein VP0. This protein plays a crucial role in immature procapsids, being cleaved into capsid proteins. It enables the capsid to remain inactive until the maturation process occurs) as a positive reference drug.

**Table 4:** Binding Affinity of ligands with selected Coxsackievirus vital protein receptors.

No	Ligands	Binding Affinity (kcal mol^−1^)		
		Coxsackievirus adenovirus receptor	3C-protease from coxsackievirus B4	3Dpol RNA dependent RNA polymerase
		(PDB: ID 2J12)	(PDB: ID 8Y2U)	(PDB: ID 3DDK)
**1**	**11a**	−5.80	−8.10	−8.50
**2**	**11b**	−6.50	−9.50	−9.50
**3**	**11c**	−6.30	−9.40	−9.40
**4**	**13**	−5.70	−5.60	−8.10
**5**	**3**	−4.75	−6.50	−7.30
**6**	**7a**	−6.80	−9.70	−10.90
**7**	**7b**	−5.60	−8.50	−9.30
**8**	**7c**	−6.80	−9.30	−9.80
**9**	**7d**	−4.50	−8.85	−9.00
**10**	**9a**	−4.80	−7.10	−8.60
**11**	**9b**	−4.50	−7.15	−8.50
**12**	**Pleconaril**	−5.70	−6.10	−7.80

##### Docking and interaction studies with Coxsackievirus Adenovirus receptor (CAR)

**Fig. 4:**
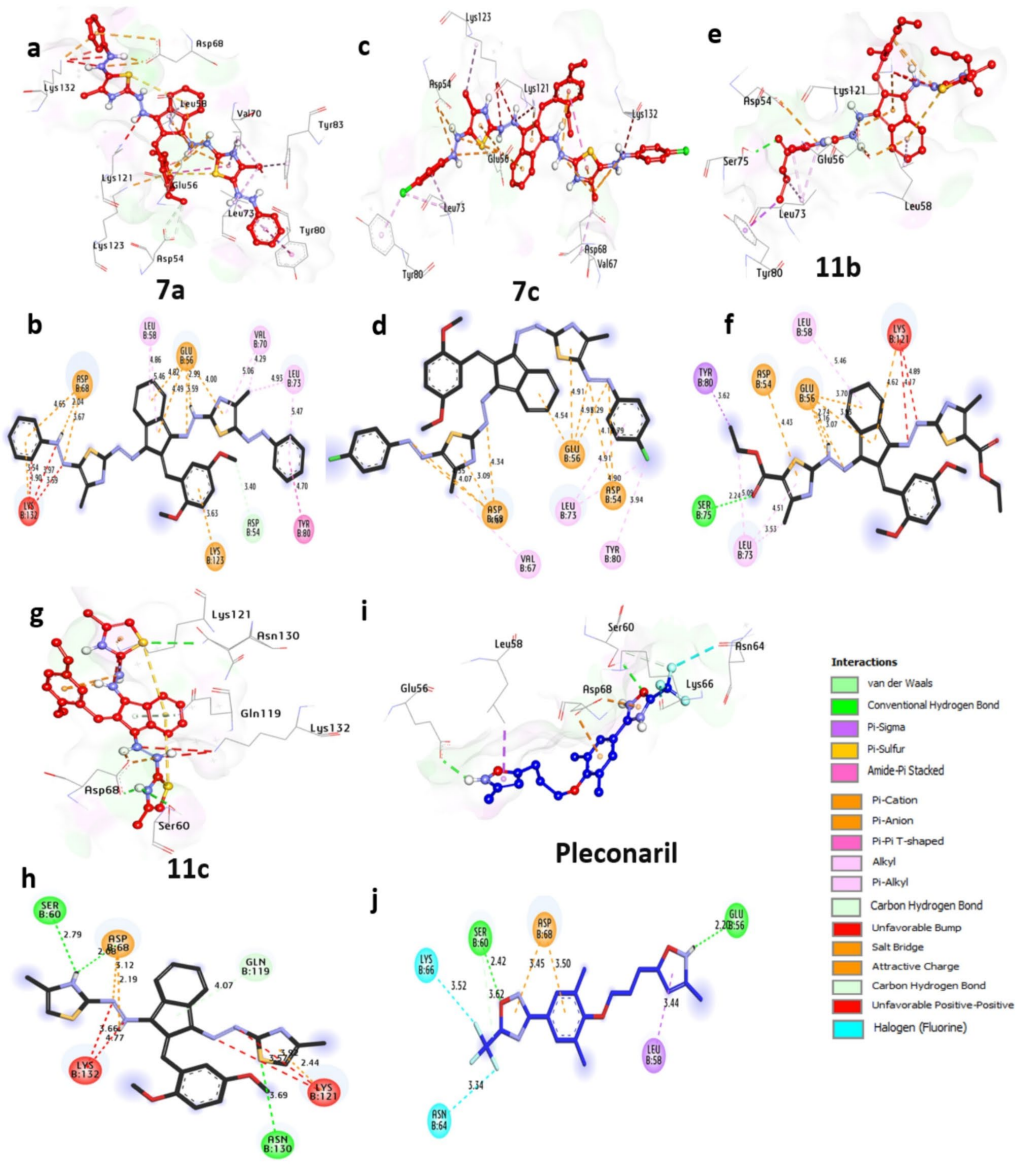
3D representations of the compounds at the binding pocket of the coxsackievirus adenovirus receptor (PDB: ID 2J12): (**a** and **b**) **7a**, (**c** and **d**) **7c**, (**e** and **f**) **11b**, (**g** and **h**) **11c**, (**I** and **j**) **Pleconaril**.

The CAR receptor is the essential protease of adenovirus and, as such, represents a promising target for the treatment of ocular and other adenoviral infections. Based on docking outcomes, the protease exhibits a significant affinity towards **7a**, **7c**, **11b**, and **11c**, showcasing binding energies of −6.80, −6.80, −6.50, and − 6.30 kcal/mol, respectively. These substances establish hydrogen bonds with key residues like Ser75, Asn130, Ser60, and Asp68, while also engaging in hydrophobic interactions such as (Pi-alkyl) with Leu73, Val70, Leu58, Tyr80, Val67, (Pi-cation) with Glu56, Asp68, Lys132, Asp54, Glu56, Asp68, and Lys121, (Pi-sigma) with Tyr80, (C-hydrogen bond) with Gln119 and Asp54, (Pi-sulfur) with Lys121, (unfavourable-bump) with Lys121, and (Pi-Pi-stacked) with Tyr80. Overall, these results indicate that **7a** and **7c** show the most promise among the compounds and merit further investigation as potential coxsackievirus adenovirus receptors. (Fig. 4 and Table S1in the supplementary data section). Our findings are similar to those of^43^, in which docking was employed to evaluate the inhibitory interaction between a molecule and adenovirus receptor protein.

##### Docking and interaction studies with 3C-protease from coxsackievirus

**Fig. 5:**
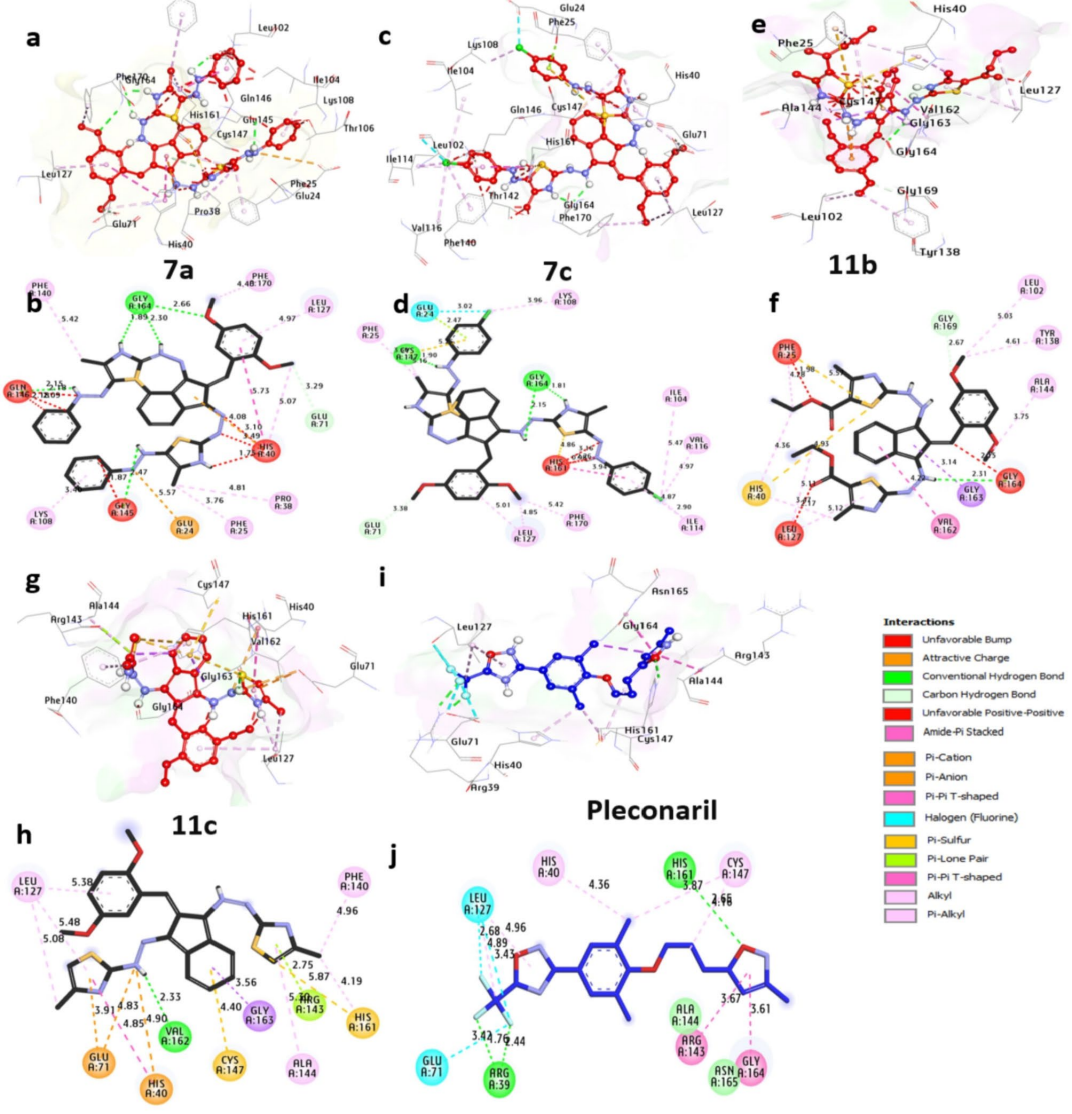
3D representations of compounds conformations at the binding pocket of 3C protease from coxsackievirus (PDB: ID 8Y2U): (**a** and **b**) **7a**, (**c** and **d**) **7c**, (**e** and **f**) **11b**, (**g** and **h**) **11c**, (**I** and **j**) **Pleconaril**.

The 3C protease derived from coxsackievirus functions as a critical viral protease enzyme pivotal in the viral replication process. This cysteine protease is responsible for cleaving viral polyprotein precursors, leading to the production of essential viral proteins crucial for viral replication. Through docking analyses, it is evident that the compound exhibits a robust affinity for **7a**, **7c**, **11b**, and **11c**, with binding energies of −9.60, −9.30, −9.50, and − 9.40 kcal/mol, respectively. These compounds establish hydrogen bonds with Gly164, Gly145, Gln146, Gly147, and Val162, while also engaging in hydrophobic interactions such as (Pi-alkyl) with Leu127, Phe170, Pro38, Lys108, Ile104, Val116, Ile114, His161, Leu102, Tyr138, Ala144, His40, Phe25, Phe140, (halogen) with Glu24, (Pi-Pi T shaped) with His40, (Pi-sulfur) with His161, His40, Phe25, and Cys147, (Pi-cation) with Gly147, Gly147, His40 and Glu71, (carbon-H-bond) with Gly169 and Glu71,(Pi-sigma) with Gly163. The amino acids Gly164, Gln146, Gly147, and Leu127 within the catalytic site appear to augment the binding affinity of the compounds significantly. In summary, these observations indicate that **7a** demonstrates substantial potential as an inhibitor of the 3C protease from coxsackievirus. (Fig. 5and Table S2 in the supplementary data section). These results were similar to^40^ in which protease was used as a therapeutic protein target for drug development.

##### Docking with 3Dpol RNA-dependent RNA polymerase (RdRp) of Coxsackievirus

**Fig. 6:**
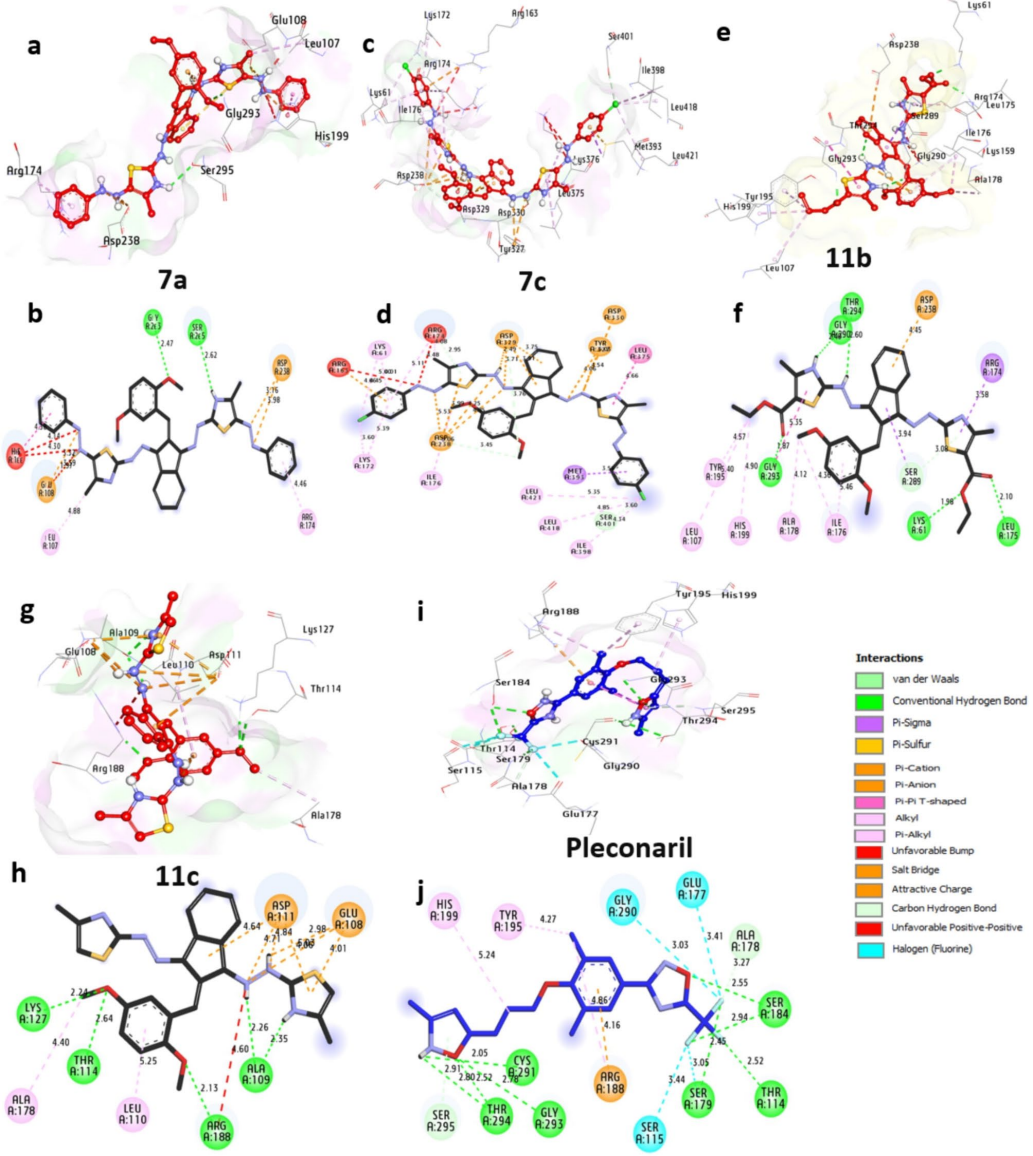
3D representations of compound conformations at the binding pocket of **RdRp** (PDB: ID 3DDK): (**a** and **b**) **7a**, (**c** and **d**) **7c**, (**e** and **f**) **11b**, (**g** and **h**) **11c**, (**I** and **j**) **Pleconaril**.

RdRp is a pivotal enzyme crucial for viral replication, facilitating the synthesis of RNA strands, a fundamental process for viral reproduction within host cells. Docking analyses indicate that compounds **7a**, **7c**, **11b**, and **11c** exhibit the highest affinity, with binding energies of −10.90, −9.80, −9.50, and − 9.40 kcal/mol, respectively. These compounds establish hydrogen bonds with Ser295, Gly290, Leu175, Lys61, Gly293, Thr294, Gly290, Ala109, Arg188, Thr114, and Lys127, while also engaging in hydrophobic interactions such as (Pi-alkyl) with Arg174, Leu107, Ile398, Leu418, Leu421, Ile176, Lys172, Lys61, Ala178, Ile176, His199, Leu107, Leu110, and Tyr195, (Pi-Pi stacked) with His199, (carbon-hydrogen bond) with Ser289, Asp329, Ser401, (Pi-sigma) with Arg174 and Met393, (Pi-anion) with Glu227, Asp330, Tyr195, Asp329, Glu108, Asp111, Asp238. The amino acids Thr294, Arg188, Thr294, and Thr114 in the catalytic site appeared to enhance the binding affinity of the compounds. Overall, the amino acids Thr294, Arg188, Thr294, and Thr114 within the catalytic site appear to significantly enhance the binding affinity of these compounds. In conclusion, these results suggest that **7a** shows promise as a potential candidate for further investigation as an inhibitor of the RdRp of Coxsackievirus. (Fig. 6and Table S3 in the supplementary data section). These results were similar to that of^44^ where they used RdRp as a viral protein receptor for explanations of antiviral activities of the compound through molecular docking analysis.

#### In silico pharmacokinetics ADMET prediction of synthesized compounds

**Fig. 7:**
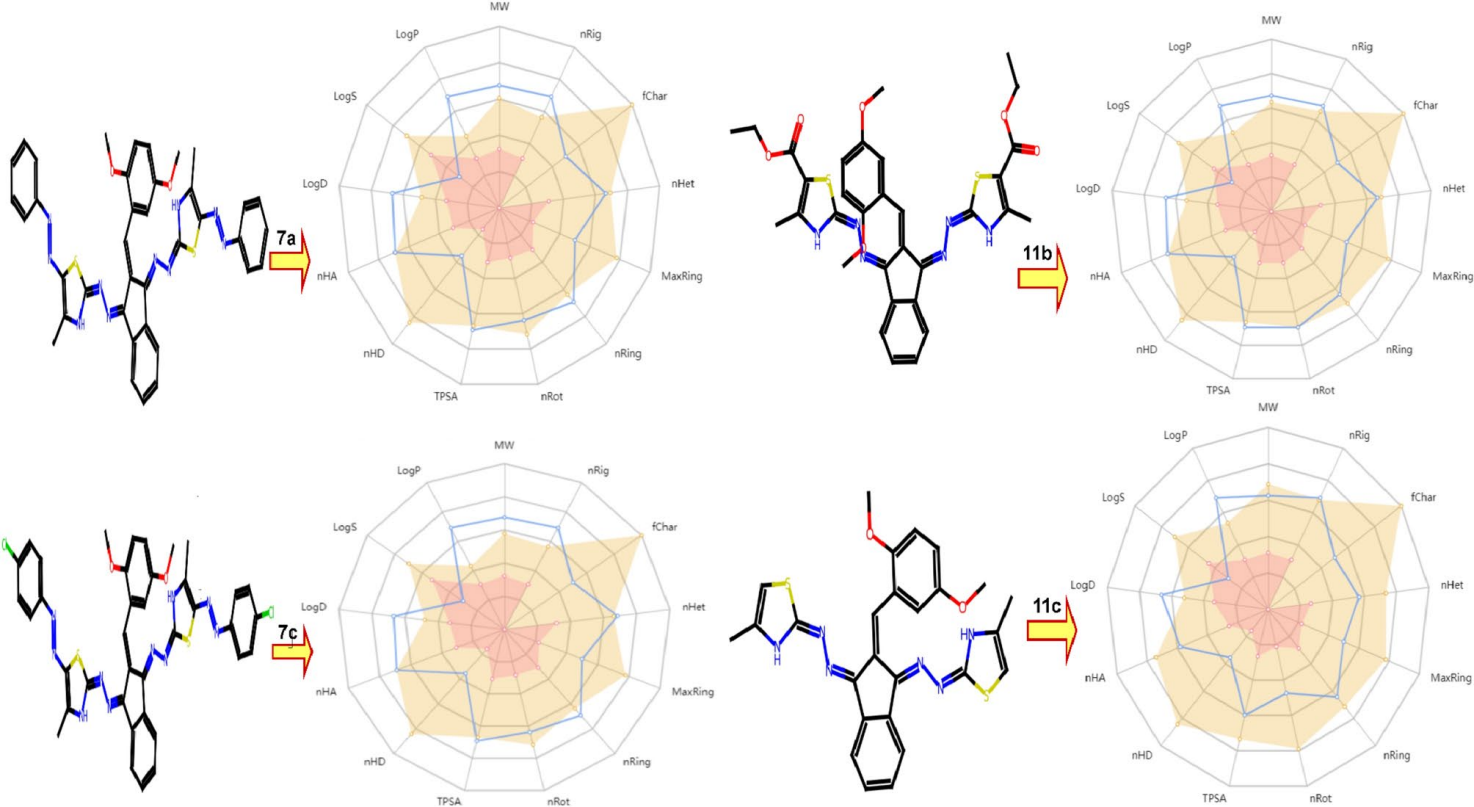
Oral bio-availability graph for compounds with the help of ADMETlab 2.0.

Based on the results of molecular docking for compounds **7a**,** 7c**,** 11b**,** and 11c**, the most promising candidates with the highest affinity concerning ADME and toxicity risks have been pinpointed. Initially, the physicochemical characteristics of the tested compounds are detailed in Table 5; Fig. 7. All relevant criteria were meticulously scrutinized. Consequently, all compounds exhibited a molecular weight exceeding 500 and possessed an adequate number of rotatable bonds (5–11 RBs), a pivotal factor for substantial structural flexibility. The count of hydrogen bond acceptors (HBA) and donors (HBD) was also determined for the four compounds, revealing that each compound featured fewer than 10 HBA and less than 5 HBD, indicating a favorable HBA-HBD equilibrium and enhanced potential for oral bioavailability. Moreover, the optimum reference TPSA were obtained as (20–160), therefore, our compounds displayed relatively high TPSA values, predominantly falling within the optimal range of 99–152 for efficient gut absorption and oral bioavailability. Subsequently, the lipophilicity and water solubility of compounds **7a**,** 7c**,** 11b**, and **11c** were evaluated. The results indicated poor water solubility across all active compounds, with Log S values ranging from − 9.894 to −6.352, signifying limited water solubility. Pharmacokinetic tests were then conducted, revealing high theoretical bioavailability for the compounds, and positioning them as promising drug candidates. However, moderate intestinal absorption was observed for all compounds, alongside the potential to interact with other drugs by inhibiting CYP2C9, CYP2C19, and CYP1A2 enzymes. Moreover, the drug-likeness of the compound was assessed through Lipinski, Golden Triangle, and Pfizer rules. All four compounds satisfied the drug-likeness requirements specified by the Pfizer Rule, suggesting promising physicochemical characteristics for drug development. Notably, all compounds, with the exception of **7a**, adhered to the Lipinski criteria, while none met the criteria set by the Golden Triangle rule. Additionally, the distribution of compounds, including Plasma Protein Binding (PPB), was assessed, revealing high levels of protein binding exceeding 99%, indicative of a low therapeutic index and minimal unbound plasma fraction. Furthermore, the Blood-Brain Barrier (BBB) penetration analysis suggested that all compounds were BBB-, incapable of crossing the blood-brain barrier. Also, according to computational assessments, compounds **7b** and **11b** seem to be relatively safe and non-toxic. Conversely, compounds **7a** and **11c** exhibit mutagenic and tumorigenic effects, as outlined in Table 6.

**Table 5:** Prediction of pharmacokinetics and physicochemical properties of compounds.

Id	ID	7a	7c	11b	11c	Id	ID	7a	7c	11b	11c
**Physicochemical Properties**	MW	724.22	792.14	660.18	516.14	**Metabolism**	CYP1A2-inh	0.612	0.703	0.822	0.969
	Vol	715.109	745.531	635.439	501.775		CYP1A2-sub	0.994	0.991	0.995	0.995
	Dense	1.013	1.063	1.039	1.029		CYP2C19-inh	0.905	0.815	0.935	0.983
	nHA	12	12	12	8		CYP2C19-sub	0.102	0.072	0.068	0.123
	nHD	2	2	2	2		CYP2C9-inh	0.944	0.846	0.981	0.955
	TPSA	148.92	148.92	152.08	99.48		CYP2C9-sub	0.139	0.148	0.072	0.244
	nRot	9	9	11	5		CYP2D6-inh	0	0	0.007	0.017
	nRing	7	7	5	5		CYP2D6-sub	0.008	0.008	0.011	0.028
	MaxRing	9	9	9	9		CYP3A4-inh	0.781	0.435	0.975	0.906
	nHet	14	16	14	10		CYP3A4-sub	0.968	0.973	0.942	0.952
	fChar	0	0	0	0	**Excretion**	CL (Clearance)	2.104	1.822	3.412	3.153
	nRig	45	45	33	31		T12	0.01	0.004	0.012	0.067
	Flex	0.2	0.2	0.333	0.161	**Toxicity**	hERG Blockers	0.001	0.002	0.002	0.003
	nStereo	0	0	0	0		H-HT	0.999	0.999	0.995	0.998
**Solubility**	LogS	−9.182	−9.894	−6.352	−6.552		DILI	0.995	0.994	0.993	0.985
	LogD	5.362	5.47	4.308	4.492		AMES Toxicity	0.915	0.768	0.981	0.952
	LogP	8.296	9.232	5.518	5.043		Rat Oral Acute Toxicity	0.737	0.702	0.118	0.874
	ESOL Log S	−11.34	−12.54	−8.72	−7.62		FDAMDD	0.999	0.998	0.997	0.996
	Ali Log S	−15.66	−16.97	−12.50	−10.25		Skin Sensitization	0.024	0.016	0.025	0.068
	Silicon-IT class	Insoluble	Insoluble	Poorly	Poorly		Carcinogenicity	0.962	0.943	0.949	0.861
**drug-likeness**	Lipinski Rule	Rejected	Accepted	Accepted	Accepted		Eye Corrosion	0.003	0.003	0.003	0.003
	Pfizer Rule	Accepted	Accepted	Accepted	Accepted		Eye Irritation	0.022	0.019	0.021	0.017
	Golden Triangle	Rejected	Rejected	Rejected	Rejected		Respiratory Toxicity	0.086	0.073	0.482	0.052
**Absorption**	Pgp-inh	0.981	0.993	0.997	0.997	**Toxicophore Rules**	**Non-Genotoxic Carcinogenicity**	1	2	0	0
	Pgp-sub	0.006	0.006	0.003	0.006		LD50_oral	0	1	0	0
	HIA	0.013	0.007	0.013	0.008		Genotoxic Carcinogenicity	0	0	0	0
	F (20%)	0.003	0.003	0.782	0.002		SureChEMBL	0	0	0	0
	F (30%)	0.01	0.004	0.023	0.001		NonBiodegradable	1	1	1	1
	Caco-2	−6.064	−6.008	−6.59	−6.417		Skin_Sensitization	1	2	0	0
	MDCK	1.11E-05	8.64E-06	2.33E-05	2.12E-05		_Aquatic Toxicity Rule	4	4	2	2
**Distribution**	BBB	0.002	0	0.034	0.571	**Medicinal Chemistry**	Toxicophores	5	5	0	0
	PPB	110.82%	114.38%	101.38%	99.04%		QED	0.113	0.111	0.187	0.372
	VDss	3.2	4.078	0.037	0.335		Synth	4.009	4.08	3.736	3.791
	Fu	1.31%	0.88%	0.89%	0.87%		Fsp3	0.105	0.105	0.25	0.154

**Table 6:** Prediction of toxicity risks and oral toxicity prediction results of compounds.

No	Ligand	Toxicity risks				Physicochemical properties				
		Mutagenic	Tumorigenic	Irritant	Reproductive	CLogP	Solubility	Molecular Weight	Drug likeness	Drug score
1	**7a**	(-)	(-)	(-)	(-)	14.31	−12.13	724.0	−4.55	0.35
2	**7c**	(+)	(+)	(-)	(-)	15.52	−13.60	792.0	−3.35	0.15
3	**11b**	(-)	(-)	(-)	(-)	10.33	−7.95	660.0	0.71	0.25
4	**11c**	(+)	(+)	(-)	(-)	9.52	−6.86	516.0	3.51	0.12

#### Molecular dynamics simulation (MDS)

**Fig. 8:**
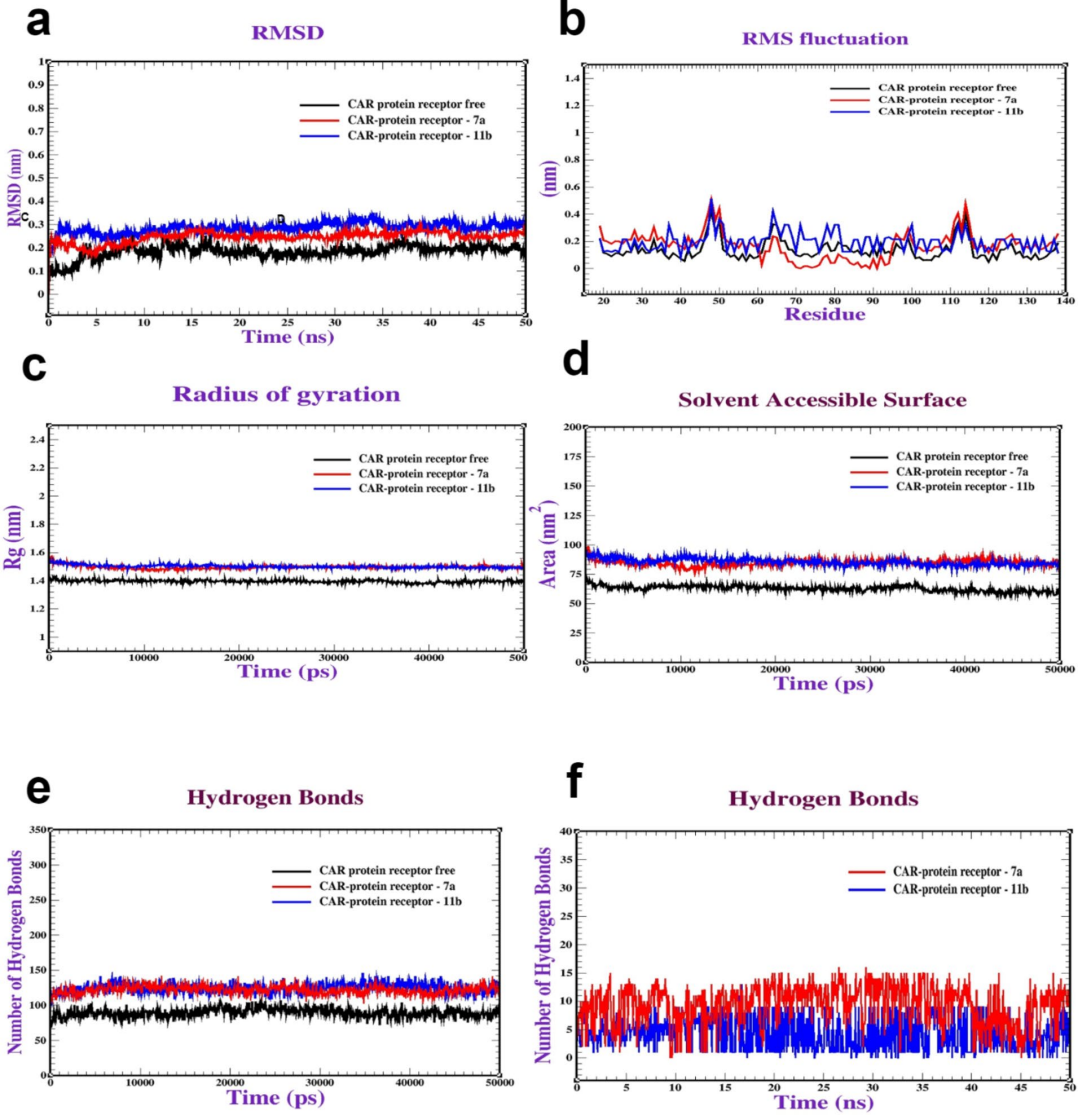
Molecular dynamics of Coxsackievirus Adenovirus Receptor (**CAR**) (PDB: ID 2J12) complexed with **7a** and **11b**: (**a**) RMSD, (**b**) RMSF, (**c**) Radius of gyration (Rg), (**d**) SASA, (**e**) Intramolecular hydrogen bonds and (**f**) Intermolecular hydrogen bonds.

**Fig. 9:**
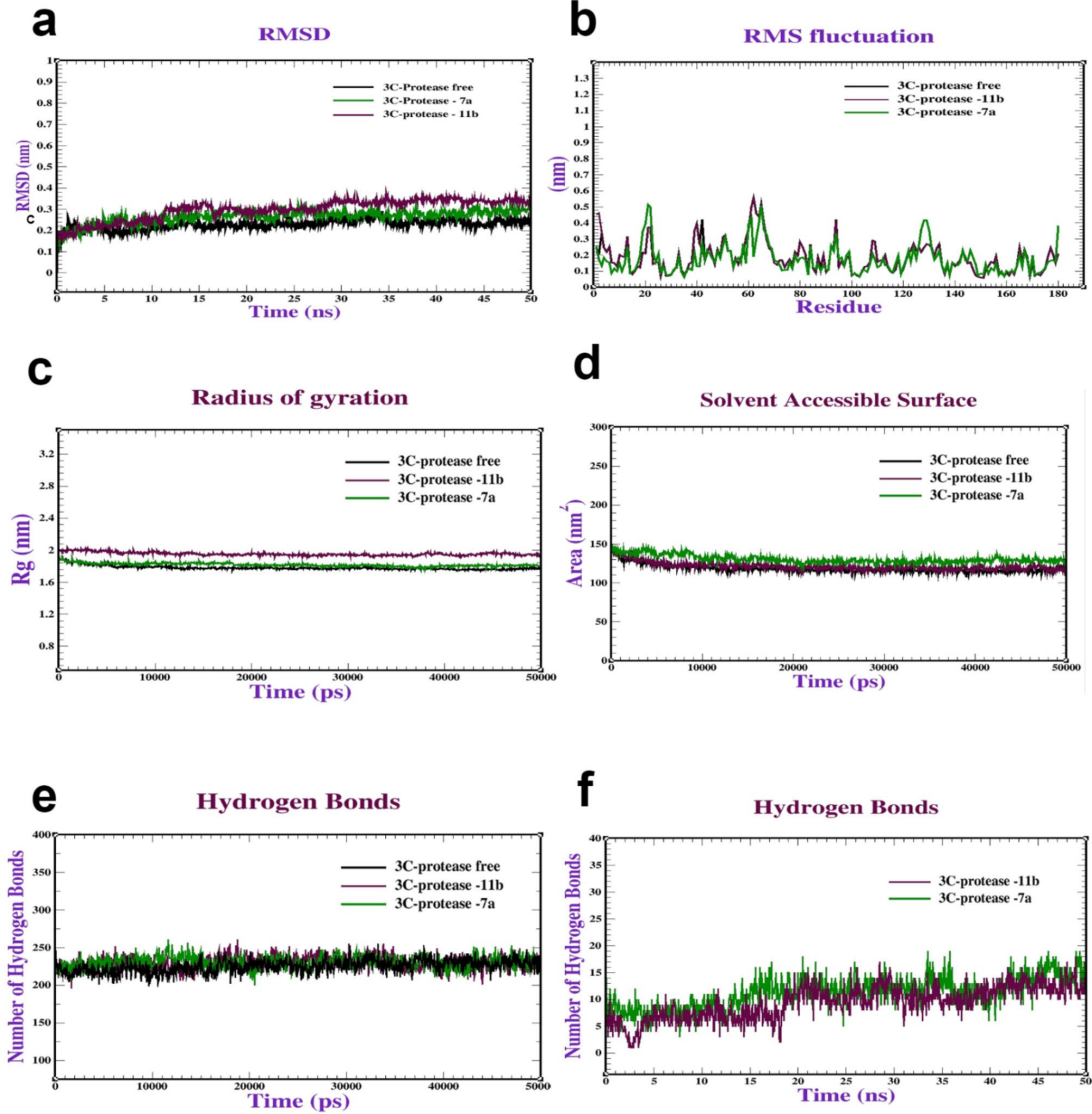
Molecular dynamics of 3C-protease from coxsackievirus (PDB: ID **8Y2U**) complexed with **7a** and **11b**: (**a**) RMSD, (**b**) RMSF, (**c**) Radius of gyration (Rg), (**d**) SASA, (**e**) Intramolecular hydrogen bonds and (**f**) Intermolecular hydrogen bonds.

**Fig. 10:**
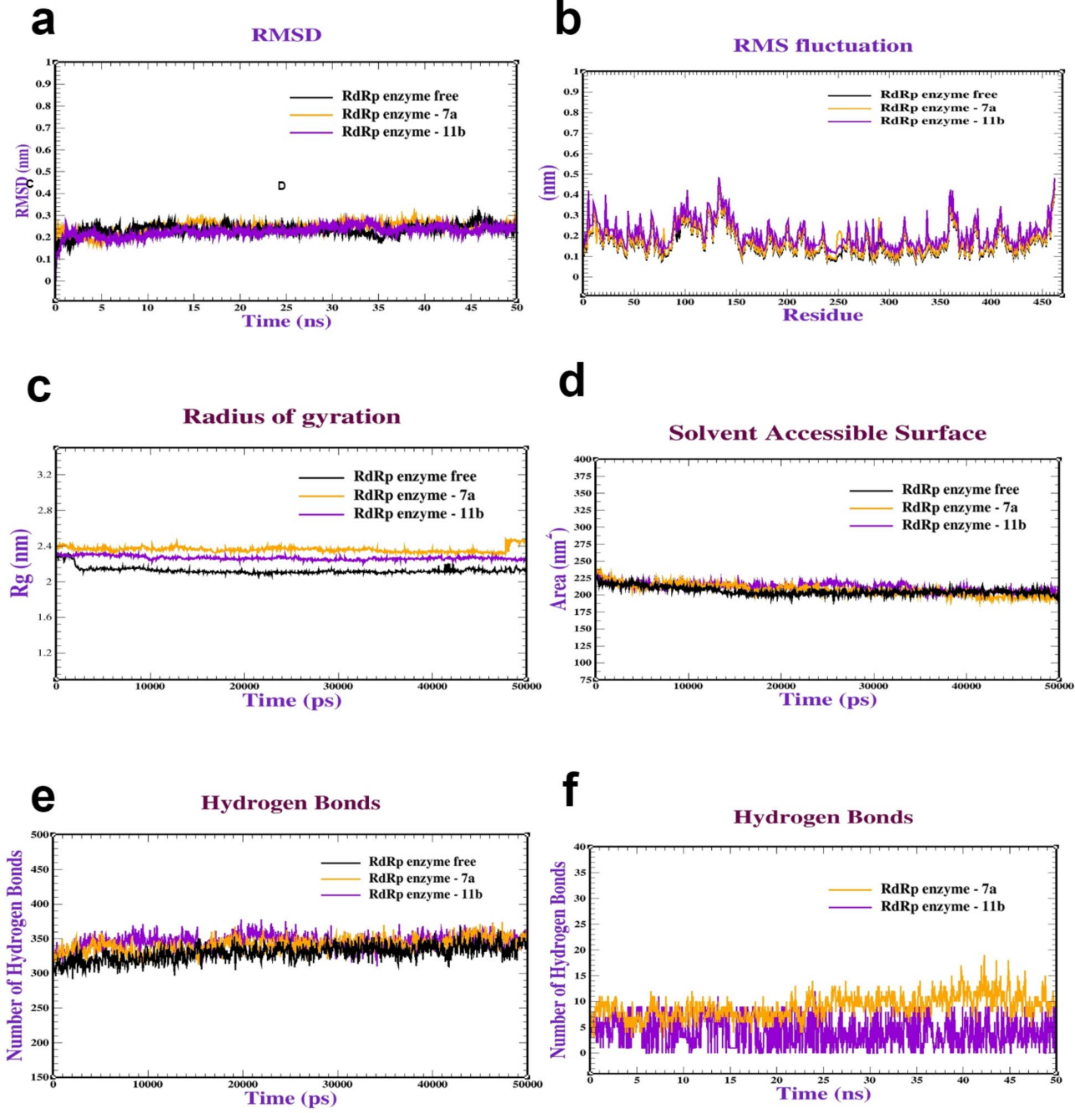
Molecular dynamics of 3Dpol RNA dependent RNA polymerase (RdRp) of Coxsackievirus (PDB: 3DDK) complexed with **7a** and **11b** : (**a**) RMSD, (**b**) RMSF, (**c**) Radius of gyration (Rg), (**d**) SASA, (**e**) Intramolecular hydrogen bonds and (**f**) Intermolecular hydrogen bonds.

Based on the docking of the three coxsackievirus vital receptors with a **7a** and **11b** compound, dynamic simulations were performed to investigate the behavior and stability of the complex at the atomic level. Firstly, the MDS of CAR complexes with **7a** and **11b** were performed to assess the stability and dynamics of the CAR complexes. The Root Mean Square Deviation (RMSD) was used to evaluate the stability of the protein structures. The results depicted in Fig. 8a indicate that both CAR-**7a** and CAR-**11b** remained stable within the ranges of (0.20–0.25 nm) and (0.25–0.30 nm), respectively, and exhibited stabilization after 10 and 15 ns. Furthermore, Root Mean Square Fluctuation (RMSF) was conducted to evaluate the flexibility of amino acid residues throughout the simulation. The majority of residues displayed minimal fluctuations (0.1–0.45 nm), signifying a state of relative stability (Fig. 8b). Moreover, Radius of gyration (Rg) analysis was performed to assess the general conformation of the protein complexes. Figure 8c illustrates Rg values for the CAR-**7a** and CAR-**11b** complexes falling within the range of 1.50 to 1.55 nm (Fig. 8c). The Rg values provide insights into the compactness or expansion of the protein structures during the simulation. Next, Surface Area of Solvent Accessible (SASA) analysis was conducted to understand the protein folding dynamics and stability. SASA values for the CAR-**7a** and CAR-**11b **complexes varied from 80 to 105 nm^2^ (Fig. 8d). Furthermore, the intramolecular and intermolecular hydrogen bonds were analyzed to assess the stability of the complexes. The complexes formed a range of 120–140 intramolecular hydrogen bonds (Fig. 8e). Also, it formed (1–12 bonds) intermolecular interactions bonds (Fig. 8f). Our MD simulations were consistent with^43^, who also performed MD simulations to validate interaction and stability of adenovirus receptor complexes. Secondly, MDs were conducted on the 3C-protease complex with **7a** and **11b**. The results presented in Fig. 9a demonstrate that the RMSD values of the 3C protease with **7a** and **11b** remained consistent within the intervals of (0.20–0.25) and (0.20–0.30) nm respectively, stabilizing after 10 and 30 ns. Additionally, RMSF analysis was performed as illustrated in Fig. 9b. The most residues exhibited minor fluctuations, typically ranging from 0.1 to 0.5 nm. Figure 9c exhibits the Rg values of 3C-protease with (**7a** and **11b**), varying between (1.80–190 nm). Furthermore, Fig. 9d depicts the SASA values for 3C-protease with (**7a** and **11b**) falling within the range of 140–150 nm^2^. Also, Fig. 9e illustrate the intramolecular hydrogen bonds in 3C-protease with (**7a** and **11b**), ranging from 220 to 245 bonds. Regarding intermolecular hydrogen bonds, 3C-protease with (**7a** and **11b**) formed intermolecular hydrogen bonds (1–10) bonds (Fig. 9f). Finally, MDs were performed to assess the stability of the RdRp complex with **7a** and **11b**. As illustrated in Fig. 10a, the RdRp-**7a** and RdRp-**11b** complexes demonstrated stability, with an RMSD ranging between 0.20 and 0.35 nm, achieving stability after 10 ns. RMSF analysis in Fig. 10b revealed minor variations, typically between 0.1 and 0.50 nm. Additionally, the Rg values for RdRp-**7a** fluctuated between 2.30 and 2.40 nm, as shown in Fig. 10c. Furthermore, Fig. 10d shows the SASA values for RdRp-**7a** and RdRp-**11b** ranged from 220 to 235 nm^2^. Lastly, Fig. (10e and 10f) showcased the intramolecular and intermolecular hydrogen bonds formed by the RdRp complex with **7a** and **11b.** Initially, the complexes exhibited a range of 300–350 intramolecular hydrogen bonds. Compounds **7a** and **11b **formed intermolecular hydrogen bonds in the range of (1–8) bonds. Our molecular dynamics were comparable to those performed by^45^, who used MD simulations to validate the contact and stability of receptor complexes.

## Conclusion

In conclusion, we succeeded in creating a new series of *bis*-thiazole derivatives linked to 2-(2,5-dimethoxy-phenyl)−1,3-dihydrazino-indane *via* the reaction of *bis*-thiosemicarbazone with hydrazonoyl chlorides and haloketones. The antiviral activity of all synthesized derivatives against Cox B revealed that *bis*-thiazole derivative **7a** is the most active derivative, affecting both virus adsorption and replication. Furthermore, through molecular docking, compounds **7a**, **7c**, **11b**, and **11c** showed strong binding energies and effective interactions with key proteins of the Cox B virus, indicating their potential as antiviral agents. These interactions, characterized by diverse chemical bonding types, suggest the potential of these compounds to inhibit enzyme activity and exhibit significant antiviral effects, impacting viral replication and adsorption. The ADMET analysis confirming adherence to Lipinski’s criteria underscores the favorable physicochemical properties of these compounds. Additionally, the MD simulations revealing stable complexes of **7a** and **11b** with essential viral proteins further support their promise in ongoing antiviral drug development efforts. The collective findings underscore the potential of these compounds as candidates for advancing antiviral therapies against Coxsackievirus adenovirus.

## Experimental

### Chemistry

#### General methods

All reagents were purchased at the highest available purity from Sigma-Aldrich and were used without further purification. Melting points were measured on a Gallenkamp melting point apparatus. IR spectra were applied on Shimadzu FT-IR 8101 PC infrared spectrophotometers (Shimadzu, Tokyo, Japan) using KBr disks. ^1^H NMR spectra were run at 400 MHz, and ^13^C NMR spectra were run at 100 MHz in deuterated dimethyl sulfoxide (DMSO-*d*_*6*_). Chemical shifts are given in parts per million and are related to that of the solvent. Mass spectra were recorded on a Bruker Daltonics spectrometer. Elemental analyses were recorded on an Elementar-Vario EL (Germany) automatic analyzer. Analytical thin-layer chromatography (TLC) was performed using silica gel 60 F254 glass plates, which was used to track each chemical reaction’s development and verify each derivative’s purity. Compound spots were visualized by UV light (254 nm).

#### Synthesis of *bis*-thiosemicarbazone derivative 3

**2**,**2’-((1*E***,**3*Z*)−2-((*E*)−2**,**5-dimethoxybenzylidene)−1*H*-indene-1**,**3(2*H*)-diylidene)bis-(hydrazine-1-carbothioamide) (3).**

In a 50 mL round Q.F. flask we added 0.01 mol of 2-(2,5-dimethoxy-benzylidene)-indan-1,3-dione **1** (2.94 g) with 0.02 moles of thiosemicarbazide **2** (≈ 2 g) in abs. ethanol (40 mL). The mixture was heated under reflux to dissolve the thiosemicarbazide. After that, 2 mL of conc. HCl was added and the reflux was completed to 2 h. Following cooling of the solution, compound 3 was obtained as yellow crystals by filtering and crystallizing the solid *bis*-thiosemicarbazone derivative 3 from ethanol/dioxane.

Yellow crystal, mp. 231–233 °C. IR (ν): 3429, 3315, 3174 (NH₂, NH), 3010 (sp^2^ CH₂), 2939 (sp^3^ CH) 1598(C = N), 1526, 1494, 1430, 1362, 1262, 1078 cm^−1^. ^1^H NMR (DMSO-d_6_) 3.67 (s, 3 H, OCH_3_), 3.73 (s, 3 H, OCH_3_), 6.88–6.94 (m, 5 H, Ar-H), 7.61 (d, *J*= 8.5 Hz, 2 H, Ar-H), 8.10 (s, 2 H, NH) 8.15 (s, 2 H, NH) 8.34 (s, 1 H, = CH) 11.38 (s, 2 H, NH). ^13^C NMR 56.1 (OCH_3_), 56.7 (OCH_3_), 110.5, 113.6, 117.9, 123.3, 138.4, 138.6, 152.8, 153.8, 178.3 (two carbon overlapped). HR-ESI-MS 441.2516 [M + H]^+^. Anal. Calcd. (Found) for: C_20_H_20_N_6_O_2_S_2_ (440.54): C, 54.53 (54.42); H, 4.58 (4.43); N, 19.08 (19.01)%.

### General method for synthesis of *bis*-thiazole derivatives 7a-d, 9a, b, 11a-c and 13

A mixture of *bis*-thiazole **3** (10 mmol) with the appropriate hydrazonyl chloride (20 mmol) **4a-d** or α-haloketones **8a**,** b**,** 10a-c** or **12** and triethylamine (few drops) in dioxane (20 mL) was refluxed till all of the starting materials have disappeared (monitored by TLC). The solvent was evaporated and the solid formed was filtered off and recrystallized from dioxane/ethanol mixture to give compounds **7a-d**, **9a**,** b**, **11a-c** and **13**.

**2**,**2’-(((1*E***,**3*E*)−2-(2**,**5-dimethoxybenzylidene)−1*H*-indene-1**,**3(2*H*)-diylidene)bis(hydrazin-1-yl-2-ylidene))bis(4-methyl-5-((*E*)-phenyldiazenyl)thiazole) (7a).**

Brown solid, mp. 123–125 °C. IR (ν): 3394 (NH), 1602 (C = N), 1541, 1491, 1356, 1253, 1221, 1171, 1039 cm^−1^. ^1^H NMR (DMSO-d_6_) 2.59 (s, 6 H, 2CH_3_) 3.71 (s, 3 H, OCH_3_), 3.80 (s, 3 H, OCH_3_) 7.01–7.44 (m, 17 H, Ar-H), 7.47 (s, 1 H, =CH), 8.77 (s, 2 H, 2NH). ^13^C NMR (DMSO-d_6_) 17.6 (2CH_3,_ two methyl overlapped), 56.0 (OCH_3_), 56.9 (OCH_3_) 103.3 110.2, 113.7, 116.7, 126.4, 129.5, 132.1, 134.8, 136.4, 145.7, 148.3, 152.7, 154.0, 168.9. HR-ESI-MS 725.1879 [M + H]^+^. Anal. Calcd. (Found) for: C_38_H_32_N_10_O_2_S_2_ (724.86): C, 62.97(62.86); H, 4.45 (4.39); N, 19.32 (19.29)%.

**2**,**2’-(((1*E***,**3*E*)−2-(2**,**5-dimethoxybenzylidene)−1*H*-indene-1**,**3(2*H*)-diylidene)bis(hydrazin-1-yl-2-ylidene))bis(4-methyl-5-((*E*)-*p*-tolyldiazenyl)thiazole) (7b).**

Dark brown solid, mp. 132–135 °C. IR (ν): 3394 (NH), 2919 (sp^3^ CH), 1608 (C = N), 1539, 1492, 1423, 1220, 1170, 1039 cm^−1^. ^1^H NMR (DMSO-d_6_) 2.21 (s, 6 H, 2CH_3_), 2.27 (s, 6 H, 2CH_3_), 3.76 (s, 3 H, OCH_3_), 3.80 (s, 3 H, OCH_3_), 7.05–7.51 (m, 16 H, ArH and = CH), 8.96 (s, 2 H, 2NH). ^13^C NMR (DMSO-d_6_) 17.6 (2CH_3,_ two methyl overlapped), 55.9 (OCH_3_), 56.7 (OCH_3_) 102.9, 103.6 109.8, 113.7, 116.4, 123.9, 129.5, 132.1, 134.8, 136.8, 152.0, 153.8, 158.6, 162.8, 168.5, 168.7.

HR-ESI-MS 753.8992 [M + H]^+^. Anal. Calcd. (Found) for: C_40_H_36_N_10_O_2_S_2_ (752.91): C, 63.81 (63.66); H, 4.82 (4.71); N, 18.60 (18.53)%.

**2**,**2’-(((1*E***,**3*E*)−2-(2**,**5-dimethoxybenzylidene)−1*H*-indene-1**,**3(2*H*)-diylidene)bis(hydrazin-1-yl-2-ylidene))bis(5-((*E*)-(4-chlorophenyl)diazenyl)−4-methylthiazole) (7c).**

Brown solid, mp. 119–121 °C. IR (ν): 3420 (NH), 2919 (sp^3^ CH), 1602 (C = N), 1545, 1488, 1422, 1249, 1167, 1087 cm^−1^. ^1^H NMR (DMSO-d_6_) 2.56 (s, 6 H, 2CH_3_) 3.78 (s, 3 H, OCH_3_), 3.82 (s, 3 H, OCH_3_), 7.06–7.46 (m, 15 H, ArH and = CH), 8.76 (s, 2 H, 2NH). ^13^C NMR (DMSO-d_6_) 16.7 (2CH_3,_ two methyl overlapped), 56.0 (OCH_3_), 57.4 (OCH_3_) 102.8, 103.7, 109.8, 112.0, 113.7, 116.4, 123.4, 126.9, 133.4, 136.1, 139.1, 150.4, 153.1, 157.0, 160.1, 170.1. HR-ESI-MS 794.2627 [M + H]^+^. Anal. Calcd. (Found) for: C_38_H_30_Cl_2_N_10_O_2_S_2_ (793.75): C, 57.50 (57.45); H, 3.81 (3.69); N, 17.65 (17.52)%.

**2**,**2’-(((1*E***,**3*E*)−2-(2**,**5-dimethoxybenzylidene)−1*H*-indene-1**,**3(2*H*)-diylidene)bis(hydrazin-1-yl-2-ylidene))bis(4-methyl-5-((*E*)-(4-nitrophenyl)diazenyl)thiazole) (7d).**

Dark brown solid, mp. 181–183 °C. IR (ν): 3434 (NH), 2920 (sp^3^ CH), 1595 (C = N), 1545, 1491, 1428, 1327, 1255, 1217, 1157, 1106, 1020 cm^−1^. ^1^H NMR (DMSO-d_6_) 2.51 (s, 6 H, 2CH_3_), 3.74 (s, 3 H, OCH_3_), 3.78 (s, 3 H, OCH_3_), 7.05–8.14 (m, 16 H, ArH and = CH), 8.75 (s, 2 H, 2NH).

^13^C NMR (DMSO-d_6_) 13.1 (2CH_3,_ two methyl overlapped), 56.0 (OCH_3_), 56.8 (OCH_3_) 104.5, 109.8, 110.6, 114.2, 116.4, 117.6, 123.3, 126.0, 128.2, 129.1, 135.2, 136.9, 138.6, 150.4, 152.2, 153.1, 154.0, 168.4. HR-ESI-MS 815.2184 [M + H]^+^. Anal. Calcd. (Found) for: C_38_H_30_N_12_O_6_S_2_ (814.85): C, 56.01 (55.93); H, 3.71 (3.59); N, 20.63 (20.58)%.

**2**,**2’-(((1*E***,**3*E*)−2-(2**,**5-dimethoxybenzylidene)−1*H*-indene-1**,**3(2*H*)-diylidene)bis(hydrazin-1-yl-2-ylidene))bis(thiazol-4(5*H*)-one) (9a).**

Yellow solid, mp. 130–132 °C. IR (ν): 3424 (NH), 2955 (sp³ CH), 1743 (C = O), 1616 (C = N), 1531, 1497, 1464, 1408, 1348, 1264 1173, 1036. ^1^H NMR (DMSO-d_6_) 3.74 (s, 3 H, OCH_3_), 3.80 (s, 3 H, OCH_3_), 4.59 (s, 4 H, 2CH₂), 6.98–7.49 (m, 7 H, Ar-H), 8.74 (s, 1 H, = CH), 11.34 (s, 2 H, NH). ^13^C NMR (DMSO-d_6_) 29.8 (2CH_2,_ two methylene overlapped.), 56.5 (OCH_3_), 57.7 (OCH_3_) 114.6, 119.9, 120.7, 122.5, 123.0, 124.2, 129.1, 131.2, 133.4, 136.5, 137.8, 148.3, 150.0, 153.1, 154.0, 159.7, 185.0. HR-ESI-MS 521.1413 [M + H]^+^. Anal. Calcd. (Found) for: C_24_H_20_N_6_O_4_S_2_ (520.58): C, 55.37 (55.28); H, 3.87 (3.74); N, 16.14 (16.04)%.

**2**,**2’-(((1*E***,**3*E*)−2-(2**,**5-dimethoxybenzylidene)−1*H*-indene-1**,**3(2*H*)-diylidene)bis(hydrazin-1-yl-2-ylidene))bis(5-methylthiazol-4(5*H*)-one) (9b).**

Buff solid, mp. 198–200 °C. IR (ν): 3424 (NH), 2939 (sp³ CH), 1723 (C = O), 1638 (C = N), 1564, 1495, 1455, 1370, 1328, 1252, 1644 cm^−1^. ^1^H NMR (DMSO-d_6_) 1.12 (d, *J* = 5.4 Hz, 6 H, 2CH_3_), 3.72 (s, 3 H, OCH_3_), 3.77 (s, 3 H, OCH_3_), 3.97 (q, *J*= 6.8 Hz, 2 H, 2CH), 7.01–7.31 (m, 7 H, Ar-H), 8.52 (s, 1 H, = CH), 11.22 (s, 2 H, NH). ^13^C NMR (DMSO-d_6_) 16.7 (2CH_3,_ two methyl overlapped), 30.2, 57.7 (OCH_3_), 58.6 (OCH_3_) 112.0, 112.9, 115.1, 118.5, 121.2, 123.0, 124.2, 125.1, 129.1, 129.9, 130.8, 135.6, 136.9, 148.3, 154.0, 155.7, 160.5, 183.2. HR-ESI-MS 549.1424 [M + H]^+^. Anal. Calcd. (Found) for: C_26_H_24_N_6_O_4_S_2_ (548.64): C, 56.92 (56.83); H, 4.41 (4.35); N, 15.32 (15.29)%.

**1**,**1’-((((1*E***,**3*E*)−2-(2**,**5-dimethoxybenzylidene)−1*H*-indene-1**,**3(2*H*)-diylidene)bis(hydrazin-1-yl-2-ylidene))bis(4-methylthiazole-2**,**5-diyl))bis(ethan-1-one) (11a).**

Creamy (Off-White) solid, mp. 218 –210 °C. IR (ν): 3439 (br. NH), 3152 (sp² CH), 3000, 2919 (SP³ CH), 1702 (C = O), 1635 (C = N), 1574, 1497, 1425, 1361, 1261, 1165, 1133, 1098 cm^−1^. ^1^H NMR (DMSO-d_6_) 1.78 (s, 6 H, 2CH_3_), 2.30 (s, 6 H, 2CH_3_), 3.70 (s, 3 H, OCH_3_), 3.75 (s, 3 H, OCH_3_) 6.91–7.29 (m, 7 H, Ar-H), 8.22 (s, 1 H, = CH), 11.18 (s, 2 H, NH). ^13^C NMR (DMSO-d_6_) 17.7 (2CH_3,_ two methyl overlapped), 23 (CH_3_), 56.6 (OCH_3_), 57.3 (OCH_3_) 103.2, 103.5, 109.1, 111.2, 113.3, 115.2, 116.4, 119.0, 122.5, 123.8, 126.9, 136.2, 155.6, 156.9, 163.6, 168.2. HR-ESI-MS 601.1593 [M + H]^+^. Anal. Calcd. (Found) for: C_30_H_28_N_6_O_4_S_2_ (600.71): C, 59.98 (59.82); H, 4.70 (4.68); N, 13.99 (13.84)%.

**Diethyl 2**,**2’-(((1*E***,**3*E*)−2-(2**,**5-dimethoxybenzylidene)−1*H*-indene-1**,**3(2*H*)-diylidene)bis-(hydrazin-1-yl-2-ylidene))bis(4-methylthiazole-5-carboxylate) (11b).**

Yellow solid, mp. 115–117 °C. IR (ν): 3428 (NH), 3074 (sp² CH), 2922 (sp³ CH), 1698 (C = O), 1621(C = N), 1578, 1495, 1427, 1372, 1313, 1275, 1169, 1045 cm^−1^. ^1^H NMR (DMSO-d_6_) 1.19 (t, *J* = 6.3 Hz, 6 H, 2CH_3_), 2.06 (s, 6 H, 2CH_3_), 3.70 (s, 3 H, OCH_3_), 3.75 (s, 3 H, OCH_3_), 4.38 (q, *J* = 7.9 Hz, 4 H, 2CH_2_), 6.34–7.23 (m, 7 H,, Ar-H), 8.23 (s, 1 H, = CH), 11.26 (s, 2 H, NH). ^13^C NMR (DMSO-d_6_) 11.8 (CH_3_) 17.9 (CH_3_), 55.6 (OCH_3_), 56.5 (OCH_3_), 59.5 (CH₂), 103.3, 103.7, 109.4, 111.5, 113.3, 115.1, 116.4, 119.4, 112.1, 123.8, 126.9, 136.9, 155.7, 156.6, 163.6, 168.4 (C = O). HR-ESI-MS 661.2856 [M + H]^+^. Anal. Calcd. (Found) for: C_32_H_32_N_6_O_6_S_2_ (660.76): C, 58.17 (58.06); H, 4.88 (4.68); N, 12.72 (12.63)%.

**2**,**2’-(((1*E***,**3*E*)−2-(2**,**5-dimethoxybenzylidene)−1*H*-indene-1**,**3(2*H*)-diylidene)bis(hydrazin-1-yl-2-ylidene))bis(4-methylthiazole) (11c).**

Brown solid, mp. 178–180 °C. IR (ν): 3439 (NH), 3078 (sp² CH), 1579 (C = N), 1494, 1428, 1373, 1274, 1216, 1167, 1137 cm^−1^. ^1^H NMR (DMSO-d_6_) 2.12 (s, 6 H, 2CH_3_), 3.75 (s, 3 H, OCH_3_), 3.81 (s, 3 H, OCH_3_), 6.34 (s, 2 H, thiazole-H), 6.91–7.24 (m, 7 H, Ar-H), 8.27 (s, 1 H, = CH), 11.20 (s, 2 H, NH). ^13^C NMR (DMSO-d_6_) 17.6 (CH_3_), 55.1, 56.5 (2OCH_3_), 102.8, 105.4, 109.4, 109.8, 113.3, 116.7, 121.6, 122.9, 124.2, 127.7, 130.0, 136.4, 147.0, 151.8, 153.5, 158.3, 168.9. HR-ESI-MS 517.1412 [M + H]^+^. Anal. Calcd. (Found) for: C_26_H_24_N_6_O_2_S_2_ (516.64): C, 60.44 (60.31); H, 4.68 (4.59); N, 16.27 (16.18)%.

**2**,**2’-(((1*E***,**3*E*)−2-(2**,**5-dimethoxybenzylidene)−1*H*-indene-1**,**3(2*H*)-diylidene)bis(hydrazin-1-yl-2-ylidene))bis(4-phenylthiazole) (13).**

Brown solid, mp. 193–195 °C. IR (ν): 3430(NH), 2922(sp^3^ CH), 1534, 1277, 1218, 1044 cm^−1^. ^1^H NMR (DMSO-d_6_) 3.76 (s, 3 H, OCH_3_), 3.81 (s, 3 H, OCH_3_), 6.94–8.42 (m, 20 H, Ar-H, =CH and thiazole-H), 11.63 (s, 2 H, 2NH). ^13^C NMR (DMSO-d_6_) 56.0, 57.4 (2OCH_3_), 102.8, 104.5, 109.8, 111.1, 111.5, 113.3, 116.4, 118.1, 123.3, 126.4, 128.2, 129.4, 130.0, 133.9, 135.2, 136.9, 139.1, 150.9, 151.3, 152.2, 152.7, 153.5, 168.4, 178.0. HR-ESI-MS 641.2004 [M + H]^+^. Anal. Calcd. (Found) for: C_36_H_28_N_6_O_2_S_2_ (640.78): C, 67.48 (67.36); H, 4.40 (4.29); N, 13.12 (13.09)%.

### Biological activity

**Compounds: **10 mg were dissolved in 1 mL (10% DMSO and 90% deionized water), sterilized by 1% antibiotic–antimycotic mixture and stored in −20.

**Cells: **Vero cell line was used. The cells were propagated in DMEM medium (Lonza, USA) supplemented with 10% fetal bovine serum (Gibco, USA), 1% antibiotic–antimycotic mixture (Lonza, USA).

**Virus: **Cox B virus was propagated and titrated to give final count of 0.9 × 10^5^ viral particle/mL.

#### Cytotoxicity assay

Cytotoxic effect of compounds was measured by inoculation different doses of compounds on tested cells then examining them microscopically after 24 h to determine any morphological changes that appeared on cells as a result of being subjected to the compounds^46^. Vero cells were seeded onto 96-well plates, which were then incubated for the whole night. After a 24-hour incubation period at 37˚C in a humidified incubator with 5% CO_2_, compounds were injected at concentrations of 10, 20, 30, 40, 50, 60, 70, 80, 90, and 100 µg /100 µl. Any morphological changes were monitored under a microscope.

**Plaque reduction assay**.

This test was done according to Tebas et al.^47^, In a 12-well plate Vero cells (10^5^ cells/mL) were cultivated for 1 day at 37^o^C. The Cox B virus was mixed with the safe concentration of the compound and incubated for 1 h at 37^o^C before being added to the cells. Following the removal of the growth media, the cells were injected with virus-extract combinations (100 mL/well) and then rinsed with PBS (Phosphate Buffer Saline). Following a one-hour contact period for virus adsorption, the cell monolayer was supplemented with one milliliter of Dulbecco’s Modified Eagles Media (DMEM) containing 2% agarose. The plates were then allowed to solidify and incubated at 37 °C until the development of viral plaques. Formalin (10%) was added for 2 h then plates were stained with crystal violet. Control wells were included where untreated virus was incubated with Vero cells and finally plaques were counted and percentage reduction in plaques formation in comparison to control wells was recorded as following:

% inhibition = (viral count(untreated) - viral count(treated))/viral count(untreated)X100.

#### Mechanism of virus inhibition

For a compound having an antiviral effect, this might be due to the effect on viral replication, the effect on viral adsorption, or the direct effect on the viral particle.

**Viral replication**^48^ Assay was carried out in a 6-well plate where Vero cells were cultivated (10^5^ cells/ml) for 1 day at 37^o^C. The cells were directly inoculated with the virus. After an hour of incubation, unadsorbed viral particles were eliminated by repeatedly washing the cells with PBS.

The compound was applied at different concentrations, and after 1 h contact time, 2 mL of DMEM medium supplemented with 2% agarose were added to the cell monolayer. Plates were left to solidify then incubated till the appearance of plaques.

**Viral adsorption**^49^ Vero cells were cultivated in a 6-well plate (10^5^ cells/mL) for 1 day at 37^o^C. The compound was applied at different concentrations in 200 mL medium without supplements and coincubated with the cells for 2 h at 4^o^C. The virus was coincubated with the pretreatment cells for one hour after the unadsorbed extract was eliminated by washing the cells three times in PBS. Next, 2 mL of DMEM supplemented with 2% agarose were added. The plates were left to solidify and incubated till the appearance of plaques.

**Virucidal**^50^ the assay was conducted in a 6-well plate with Vero cells that were cultured for two days at 37 °C (10^5^ cells/mL). A volume of 200 mL serum-free DMEM containing 1.5 × 10^2^ PFU from Cox B was added to the concentration of the compound resulting in viral inhibition, after 1 h incubation, The mixture was diluted three times using serum-free media, which leaves almost no extract but still permits the existence of virus particles to develop on Vero cells. 100 mL of each dilution was then introduced to the Vero cell monolayer. The DMEM overlayer was added to the cell monolayer after a one-hour contact period. The plates were left to solidify then incubated till the appearance of plaques.

### Computational methods

#### Molecular docking simulation

**Fig. 11:**
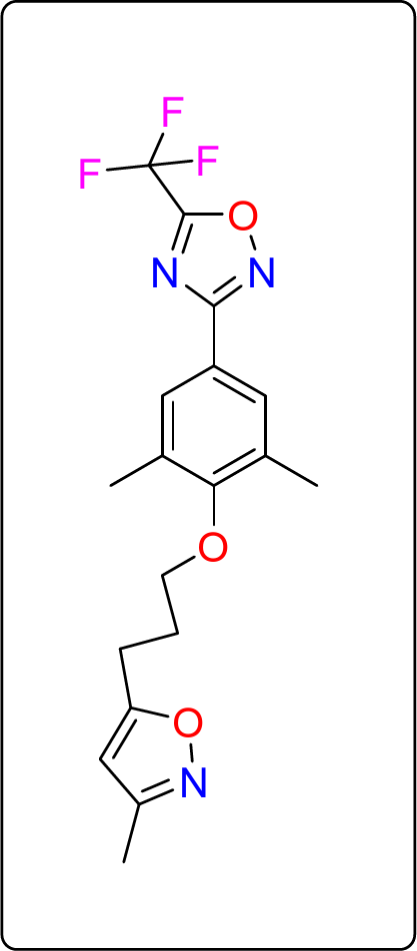
The structure of Pleconaril.

To investigate the antiviral ability of the promising compounds, Cox B virus protein receptors were obtained from the Protein Data Bank, as listed in Table (7). The crystal structures of the target receptors were preprocessed by removing water molecules, ions, and existing ligands using PyMOL software. Remarkably, one of the reference antiviral drugs used as a positive ligand for docking with the chosen COX B structure was Pleconaril (see Fig. 11). Subsequently, hydrogen atoms were added to the receptor molecule using Autodock Vina and saved in a pdbqt format. Moreover, each compound was minimized and converted to a mol2 format using Open Babel^51^. Ligand-centered maps were generated using the AutoGrid program. Additionally, the 2-D bond interactions between the target and ligands were analyzed using the Discovery Studio 4.5 program.

**Table 7:** List of target proteins, PDB IDs, active site coordinates, native ligands, and references.

No	Protein Targets	PDB ID	Resolution Å	Active site coordinates:			Reference Ligands	Co-crystalized ligand	Binding site residues	RMSD Value	Reference
				X	Y	Z					
1	Coxsackievirus adenovirus (CAR)	**2J12**	1.50 Å	24.50	9.21	3.54	**Pleconaril**	**-**	**Ser60** **Glu56**	0.54	^**52**^
**2**	3C-protease of coxsackievirus	**8Y2U**	2.01 Å	25.43	5.22	10.0	**Pleconaril**	**-**	**His40** **Glu71** **Cys147**	0.45	^**53**^
**3**	3Dpol RNA dependent RNA polymerase (RdRp)	**3DDK**	2.25 Å	6.50	38.55	−14.50	**Pleconaril**	**G74**	**Thr143** **Arg144** **Ala145**	1.54	^**54**^

#### In-silico pharmacokinetics ADME and toxicity prediction

The physicochemical parameters and ADMET of compounds were calculated using the ADMETlab 2.0^55^.

#### Molecular dynamics (MD) simulation

Molecular dynamics (MD) simulation is widely used to explain protein-ligand complexes’ binding interactions and binding affinities. In this study, MD simulations were performed using GROMACS 2018 software to further verify the rationality and reliability of the docking results. The topology of the protein was constructed using the CHARMM36 force field parameters. Also, the topology of compounds was generated using the Geoff server. After coordinating position restrictions were placed on ligands. NVT and NPT equilibrium were performed for 1000 ps in 300 K under a 1.0 bar atmosphere. After the MD simulations, Root Mean Square Deviation (RMSD), Root Mean Square Fluctuation (RMSF), and radius of gyration (Rg) were calculated^56^.

## Electronic supplementary material

Below is the link to the electronic supplementary material.

Supplementary Material 1

## Data Availability

Correspondence and requests for materials should be addressed to D.E. or S.M.
