# Supplementary material for: Synthesis, characterization, molecular docking, pharmacokinetics, and molecular dynamics of new bis-thiazoles based on bis-thiosemicarbazone as anti-coxsackievirus
Source: Sci Rep. 2024 Nov 26;14:29378. doi: 10.1038/s41598-024-80753-z (PMC11599599; doi:10.1038/s41598-024-80753-z)
Supplement: Supplementary file 1 — Supplementary Material 1 [file 41598_2024_80753_MOESM1_ESM.pdf]

SUPPLEMENTARY MATERIAL TO

Synthesis, Characterization, Molecular Docking, Pharmacokinetics, and Molecular Dynamics of New *Bis*-Thiazoles Based on *Bis*-Thiosemicarbazone as Antiviral Agents with Mode of Action

Thoraya A. Farghaly,[a] Eman M. H. Abbas,[b] Heba S. Abd-Elghaffar,[c] Mohamed A. Elsayed,[c] Dina H. Elnaggar,[c*] Ahmed F. El-Sayed,[d,e] Dina N. Abd-Elshafy,[f,g] and Salwa F. Mohamed.[c*]

Table (S1): Molecular interactions of ligands with amino acids of Coxsackievirus Adenovirus Receptor (CAR) (PDB: ID 2J12)

No	Protein	Ligand	3D Structure	Hydrophilic Interactions		Hydrophobic Contacts		No. of H-Bonds	No. of Total Bonds	affinity kcal mol^{-1}
				Residue (H- Bond)	Length	Residue (Bond type)	Length			
1	coxsackievirus receptor (PDB:ID 2J12)	7a	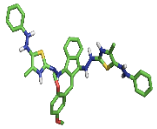	-	-	Tyr80, (Pi-Pi-stacked)	4.70	0	18	-6.80
						Leu73, (Pi-alkyl)	5.47			
						Leu73, (Pi-alkyl)	4.93			
						Val70, (Pi-alkyl)	4.29			
						Leu58, (Pi-alkyl)	4.86			
						Asp54, (C-hydrogen bond)	3.40			
						Lys123, (Pi-cation)	3.63			
						Glu56, (Pi-cation)	4.49			
						Asp68, (Pi-cation)	3.67			
						Lys132, (Pi-cation)	2.99			
2		7c	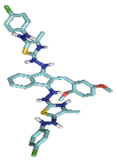	-	-	Leu73, (Pi-alkyl)	4.91	0	14	-6.80
						Leu73, (Pi-alkyl)	4.90			
						Tyr80, (Pi-alkyl)	3.94			
						Val67, (Pi-alkyl)	4.66			
						Asp54, (Pi-cation)	4.90			
						Glu56, (Pi-cation)	4.54			
						Glu56, (Pi-cation)	4.91			
						Asp68, (Pi-cation)	4.34			
						Asp68, (Pi-cation)	3.09			

3		**11b**	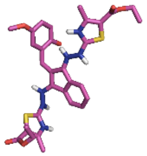	Ser75, (H- Bond)	2.24	Tyr80, (Pi-sigma)<br>Leu73, (Pi-alkyl)<br>Leu73, (Pi-alkyl)<br>Leu73, (Pi-alkyl)<br>Leu58, (Pi-alkyl)<br>Asp54, (Pi-cation)<br>Glu56, (Pi-cation)<br>Glu56, (Pi-cation)<br>Glu56, (Pi-cation)<br>Lys121, (Pi-cation)	3.62<br>4.51<br>3.53<br>5.09<br>5.46<br>4.43<br>3.70<br>2.27<br>3.53<br>4.62	**1**	**16**	**-6.50**
4		**11c**	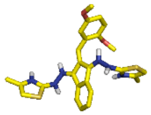	Asn130, (H- Bond)<br>Ser60, (H- Bond)<br>Asp68, (H- Bond)	3.69<br>2.79<br>3.69	Gln119, (C-hydrogen bond)<br>Lys121, (Pi-sulfur)<br>Lys121,(unfavourable-bump)<br>Asp68, (Pi-anion)<br>Asp68, (Pi-cation)	4.07<br>2.44<br>3.57<br>3.12<br>2.19	**3**	**11**	**-6.30**
5		**Pleconaril**	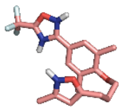	Ser60, (H- Bond)<br>Glu56, (H- Bond)	2.42<br>2.20	Ala120, ((Pi-sigma)<br>Cys122, ((Pi-cation)<br>Leu201, ((Pi-cation)<br>Ser60, (C-hydrogen bond)<br>Lys66, (halogen)<br>Asn64, (halogen)	5.26<br>6.11<br>5.12<br>3.56<br>3.52<br>3.34	**2**	**8**	**-5.70**

Table (S2): Molecular interactions of ligands with 3C-protease from coxsackievirus (PDB: ID 8Y2U):

	Protein	Ligand	3D Structure	Hydrophilic Interactions		Hydrophobic Contacts		No. of	No. of Total Bonds	affinity kcal mol^{-1}
				Residue (H- Bond)	Length	Residue (Bond type)	Length			
1	3C-protease from coxsackievirus (PDB: ID 8Y2U)	7a	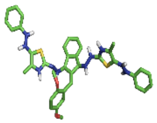	Gly164, (H- Bond)<br>Gly164, (H- Bond)<br>Gly164, (H- Bond)<br>Gly145, (H- Bond)<br>Gln146, (H- Bond)	2.66<br>2.30<br>1.89<br>2.47<br>2.15	His40, (Pi-Pi T shaped)<br>Glu71, (carbon H- bond)<br>Leu127, (Pi-alkyl)<br>Phe170, (Pi-alkyl)<br>His40, (Pi-alkyl)<br>Pro38, (Pi-alkyl)<br>Phe25, (Pi-alkyl)<br>Lys108, (Pi-alkyl)<br>Glu24, (Pi-cation)<br>Lys108, (Pi-alkyl)	5.73<br>3.29<br>4.97<br>4.48<br>5.07<br>4.81<br>3.76<br>3.40<br>5.27<br>5.42	5	21	-9.60
2		7c	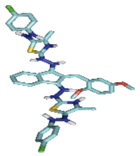	Gly164, (H- Bond)<br>Gly164, (H- Bond)<br>Gly147, (H- Bond)	1.81<br>2.15<br>5.16	Glu24, (halogen)<br>Glu71, (carbon H- bond)<br>Phe25, (Pi-alkyl)<br>Lys108, (Pi-alkyl)<br>Ile104, (Pi-alkyl)<br>Val116, (Pi-alkyl)<br>Ile114, (Pi-alkyl)<br>Phe170, (Pi-alkyl)<br>Leu127, (Pi-alkyl)<br>Leu127, (Pi-alkyl)<br>His161, (Pi-alkyl)<br>Gly147, (Pi-cation)<br>His161, (Pi-sulfur))	3.02<br>3.38<br>3.60<br>3.96<br>5.47<br>4.97<br>2.90<br>5.42<br>4.85<br>5.01<br>3.94<br>5.21<br>4.86	3	16	-9.30

3		11b	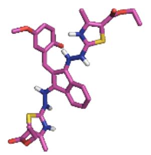	Gly164, (H- Bond)	2.31	Gly169, (carbon H- Bond)<br>Leu102, (Pi-alkyl)<br>Tyr138, (Pi-alkyl)<br>Ala144, (Pi-alkyl)<br>Leu127, (Pi-alkyl)<br>His40, (Pi-alkyl)<br>Phe25, (Pi-alkyl)<br>Gly163, (Pi-sigma)<br>His40, (Pi-sulfur)<br>Phe25, (Pi-sulfur))	2.67<br>5.03<br>4.61<br>3.75<br>5.12<br>3.22<br>4.28<br>3.14<br>4.93<br>5.57	1	17	-9.50
4		11c	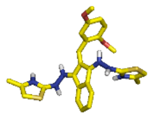	Val162, (H- Bond)	2.33	Phe140, (Pi-alkyl)<br>His161, (Pi-alkyl)<br>Ala144, (Pi-alkyl)<br>Leu127, (Pi-alkyl)<br>Gly163, (Pi-sigma)<br>Cys147, (Pi-sulfur)<br>His161, (Pi-sulfur))<br>His40, (Pi-cation)<br>Glu71, (Pi-cation)	4.96<br>4.19<br>5.30<br>5.08<br>3.56<br>4.40<br>5.87<br>4.90<br>3.91	1	15	-9.40
5		Pleconaril	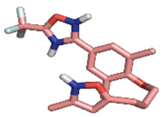	His161, (H- Bond)<br>Arg39, (H- Bond)<br>Arg39, (H- Bond)	4.66<br>2.44<br>4.76	Glu71, (halogen)<br>Leu127, (halogen)<br>Arg143, (Pi-Pi T-shaped)<br>Gly164, (Pi-Pi T-shaped)<br>Leu127, (Pi-alkyl)<br>His40, (Pi-alkyl)<br>Cys147, (Pi-alkyl)	2.68<br>3.42<br>3.67<br>3.61<br>3.87<br>4.36<br>2.56	3	15	-6.10

Table (S3): Molecular interactions of ligands with amino acids of RdRp (PDB: ID 3DDK):

	Protein	Ligand	3D Structure	Hydrophilic Interactions		Hydrophobic Contacts		No. of H-Bonds	No. of Total Bonds	affinity kcal mol^{-1}
				**Residue (H- Bond)**	**Length**	**Residue (Bond type)**	**Length**			
1	**RNA Dependent RNA Polymerase of Coxsackievirus**	**7a**	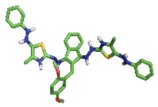	**Ser295, (H- Bond)**<br>**Gly290, (H- Bond)**	**2.62**<br>**2.47**	**Arg174, (Pi-alkyl)**<br>**Leu107, (Pi-alkyl)**<br>**Glu108, (Pi-anion)**<br>**Asp238, (Pi-cation)**<br>**His199, (Pi-Pi stacked)**<br>**Glu227, (Pi-anion)**	**4.46**<br>**4.88**<br>**3.39**<br>**3.98**<br>**4.55**<br>**3.30**	**2**	**11**	**-10.90**
2		**7c**	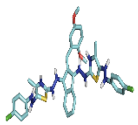	-	-	**Ile398, (Pi-alkyl)**<br>**Leu418, (Pi-alkyl)**<br>**Leu421, (Pi-alkyl)**<br>**Ile176, (Pi-alkyl)**<br>**Lys172, (Pi-alkyl)**<br>**Lys61, (Pi-alkyl)**<br>**Arg174, (Pi-alkyl)**<br>**Met393, (Pi-sigma)**<br>**Asp330, (Pi-anion)**<br>**Tyr195, (Pi-anion)**<br>**Asp329, (Pi-anion)**<br>**Ser401, (carbon H-bond)**<br>**Asp329, (carbon H-bond)**<br>**Asp238, (carbon H-bond)**	**4.34**<br>**4.85**<br>**5.35**<br>**5.53**<br>**3.60**<br>**4.40**<br>**5.11**<br>**3.55**<br>**4.54**<br>**4.06**<br>**3.75**<br>**3.60**<br>**3.76**<br>**3.45**	**0**	**24**	**-9.80**

3		11b	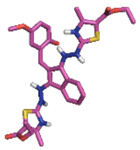	Leu175, (H- Bond)<br>Lys61, (H- Bond)<br>Gly293, (H- Bond)<br>Thr294, (H- Bond)<br>Gly290, (H- Bond)	2.10<br>1.98<br>1.87<br>2.60<br>2.44	Ala178, (Pi-alkyl)<br>Ile176, (Pi-alkyl)<br>His199, (Pi-alkyl)<br>Leu107, (Pi-alkyl)<br>Tyr195, (Pi-alkyl)<br>Arg174, (Pi-sigma)<br>Asp238, (Pi-anion)<br>Arg174, (Pi-sigma)<br>Ser289, (carbon H-bond)	4.36<br>4.12<br>4.90<br>5.40<br>4.57<br>3.58<br>4.45<br>3.94<br>3.08	5	16	-9.50
4		11c	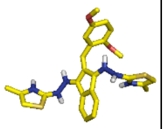	Ala109, (H- Bond)<br>Ala109, (H- Bond)<br>Arg188, (H- Bond)<br>Thr114, (H- Bond)<br>Lys127, (H- Bond)	2.35<br>2.26<br>2.13<br>2.64<br>2.24	Ala178, (Pi-alkyl)<br>Leu110, (Pi-alkyl)<br>Glu108, (Pi-anion)<br>Glu108, (Pi-anion)<br>Asp111, (Pi-anion)<br>Asp111, (Pi-anion)	4.40<br>5.26<br>4.01<br>2.98<br>4.64<br>4.71	5	11	-9.40
5		Pleconaril	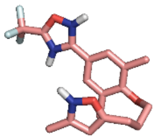	Ser184, (H- Bond)<br>Ser184, (H- Bond)<br>Thr114, (H- Bond)<br>Ser179, (H- Bond)<br>Cys291, (H- Bond)<br>Gly293, (H- Bond)<br>Thr294, (H- Bond)<br>Thr294, (H- Bond)	2.55<br>2.94<br>2.52<br>2.45<br>2.05<br>2.78<br>2.80<br>2.91	His199, (Pi-alkyl)<br>Tyr195, (Pi-alkyl)<br>Arg188, (Pi-alkyl)<br>Arg188, (Pi-anion)<br>Glu177, (halogen)<br>Gly290, (halogen)<br>Ser179, (halogen)<br>Ser115, (halogen)<br>Ala178, (carbon H-bond)<br>Ser295, (carbon H- Bond)	5.24<br>4.27<br>4.86<br>4.16<br>3.41<br>3.03<br>3.44<br>3.05<br>3.27<br>2.91	8	18	-8.20

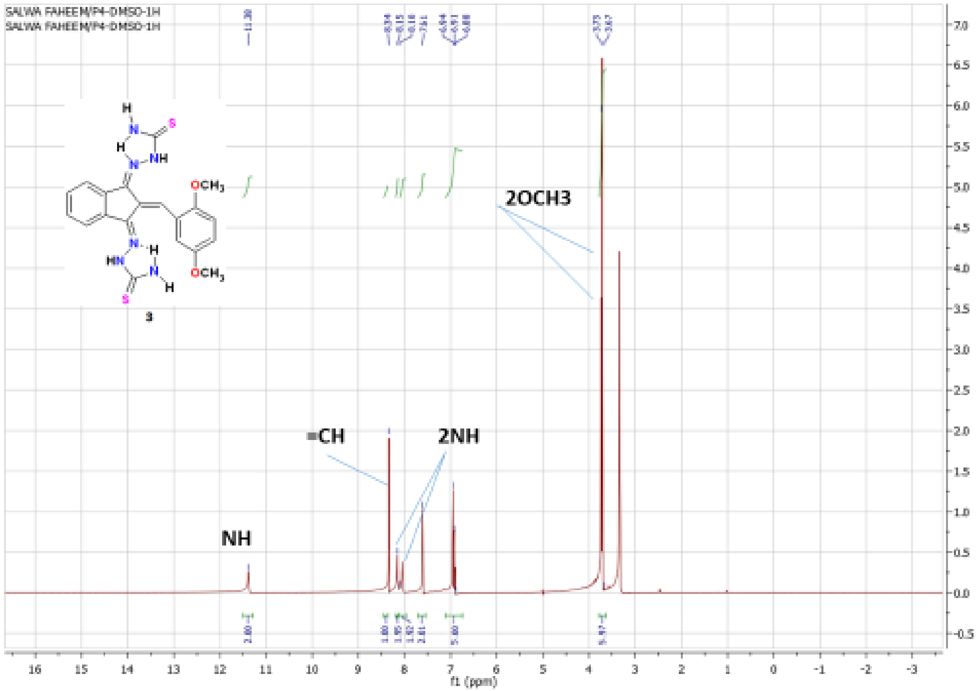

Figure S1. The ^{1}H NMR spectrum of bis-thiosemicarbazone derivative **3** declares the H-bond of one of its NH_2 protons with the C=N

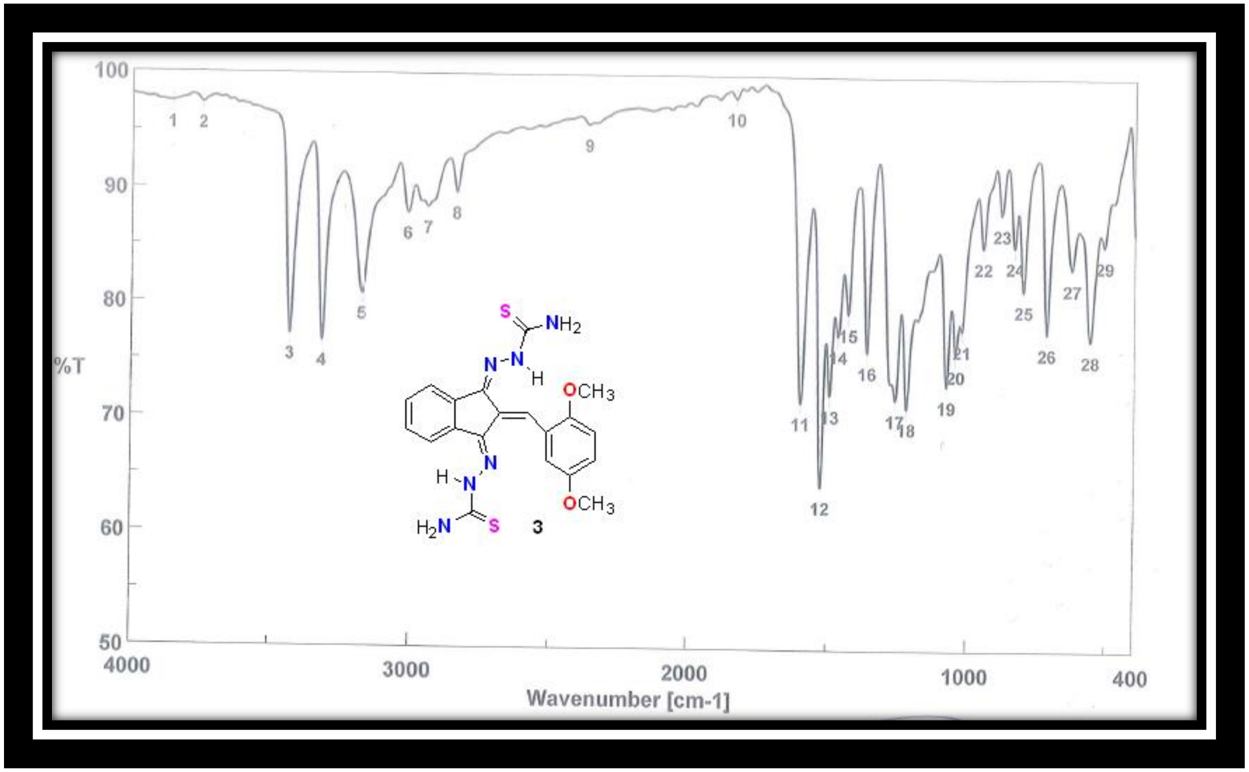

Figure S2. IR spectrum of compound (**3**)

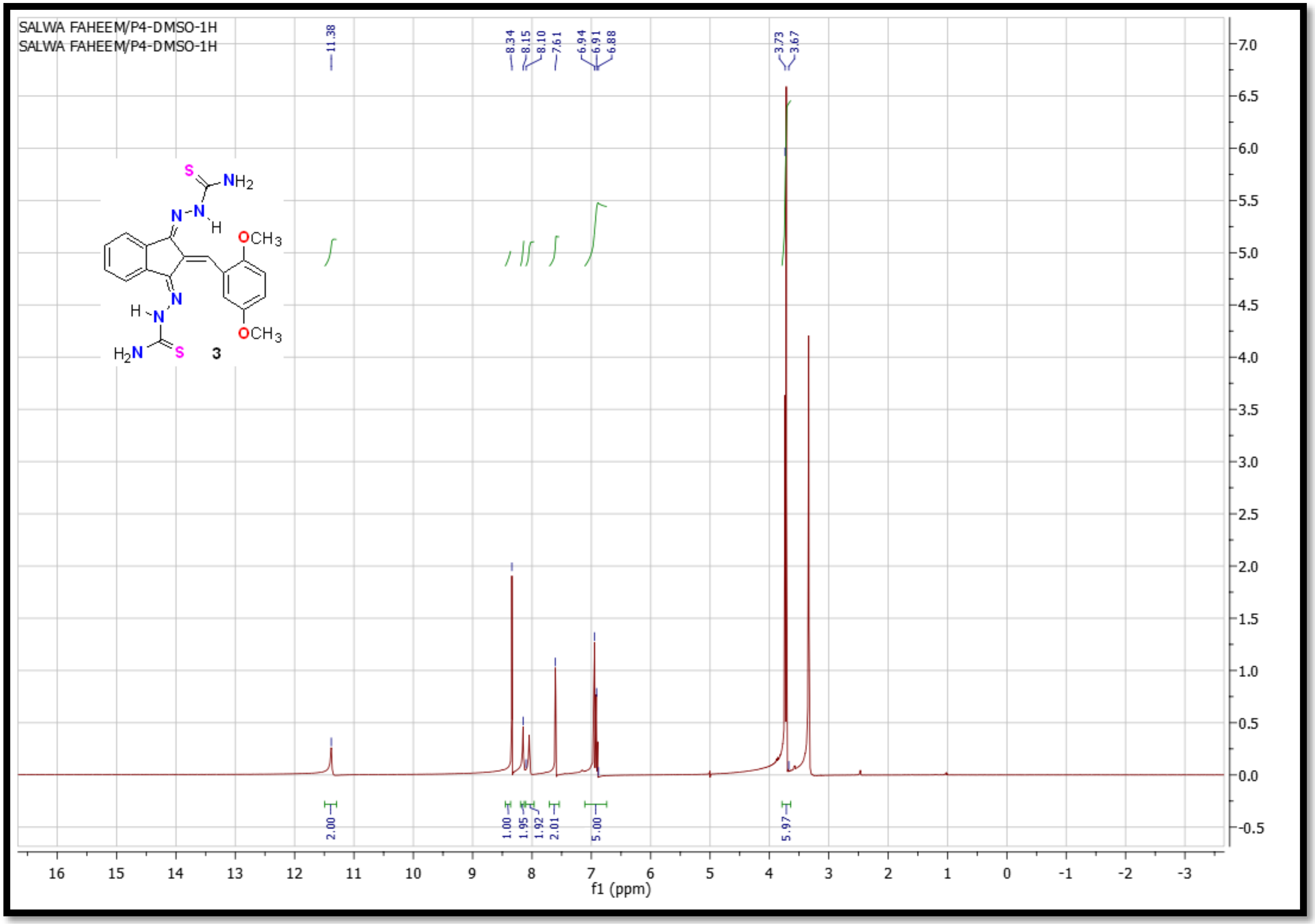

Figure S3. ^{1}H-NMR spectrum of compound (**3**)

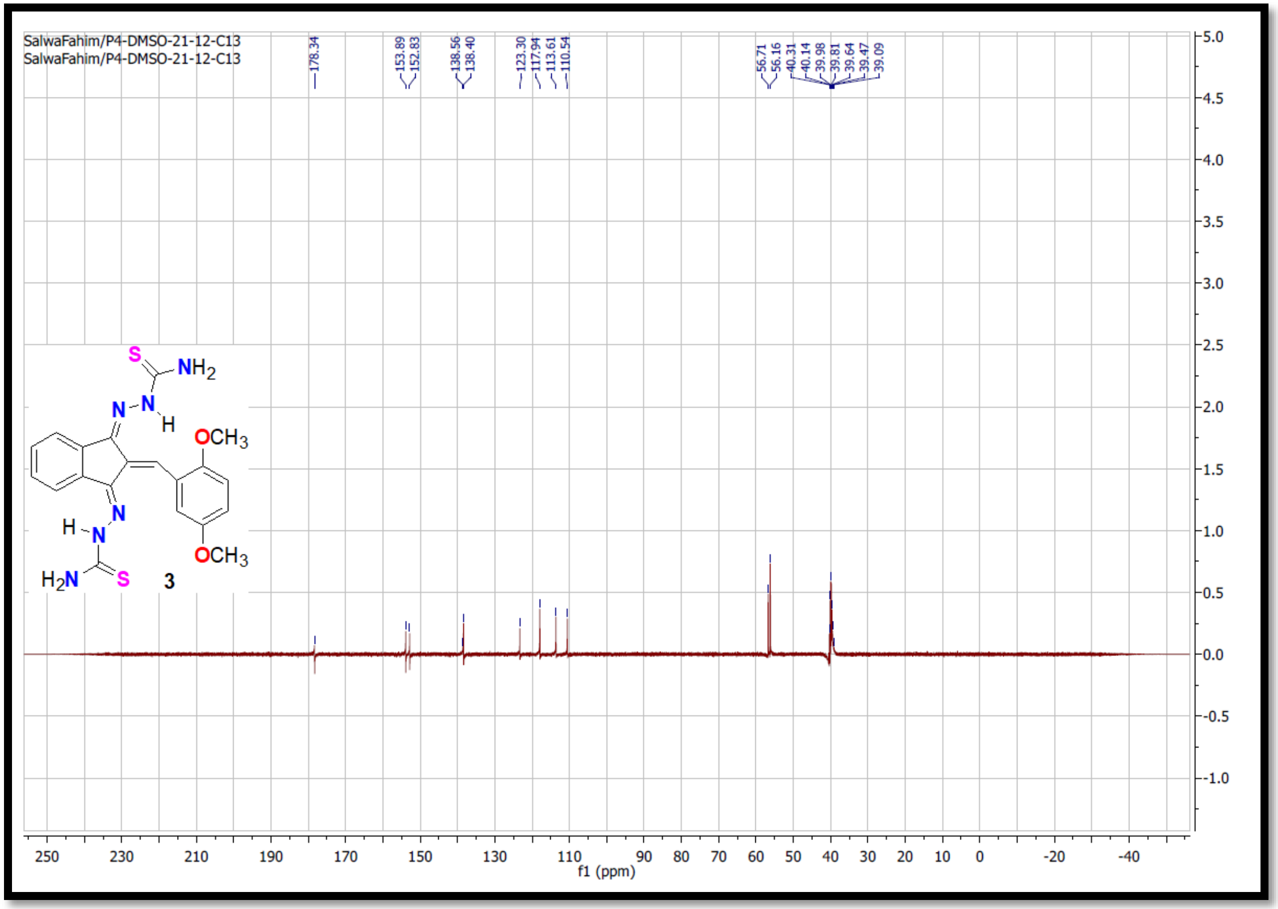

Figure S4. ^{13}C-NMR spectrum of compound (**3**)

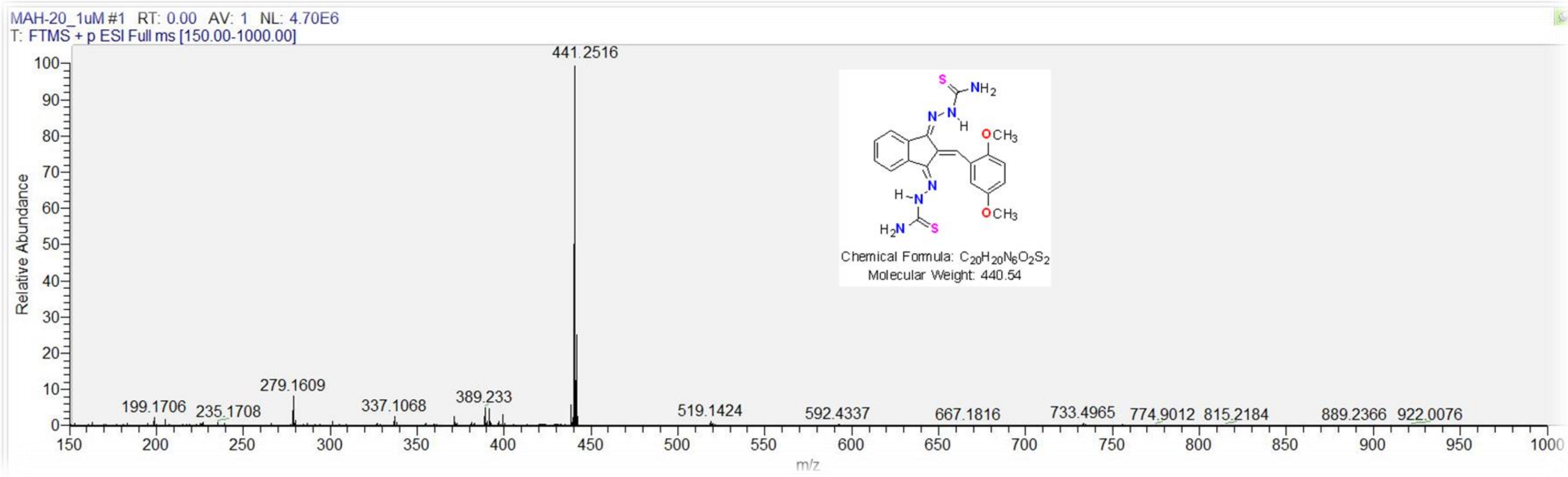

Figure S5. Mass spectrum of compound (**3**)

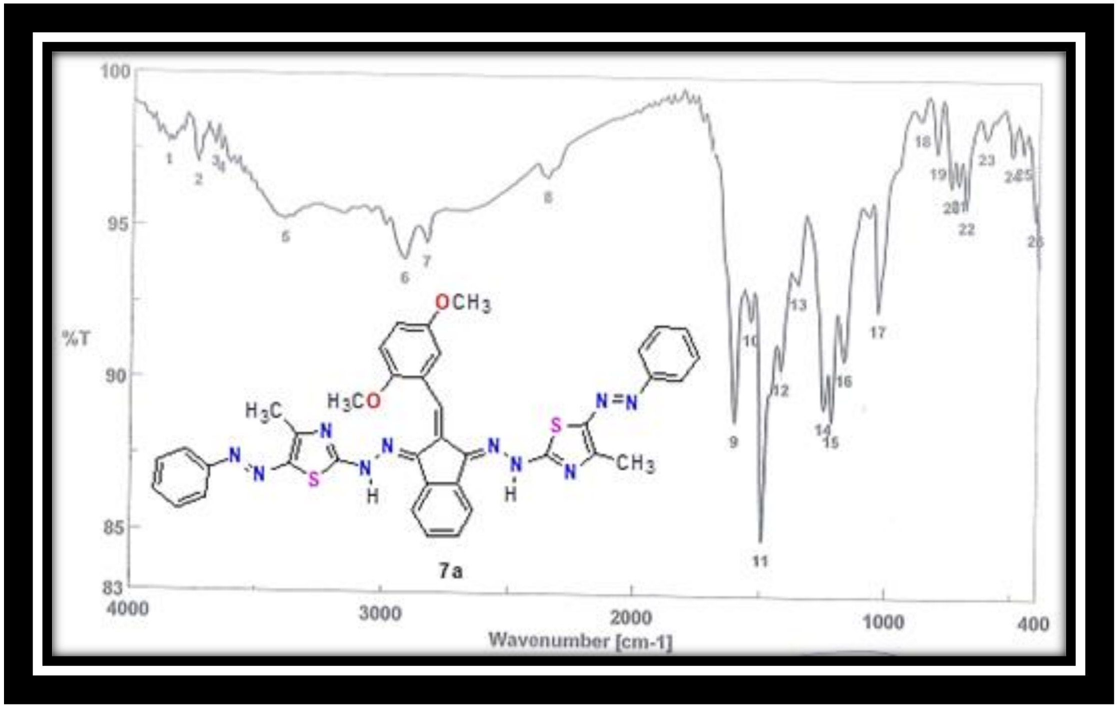

Figure S6. IR spectrum of compound (**7a**)

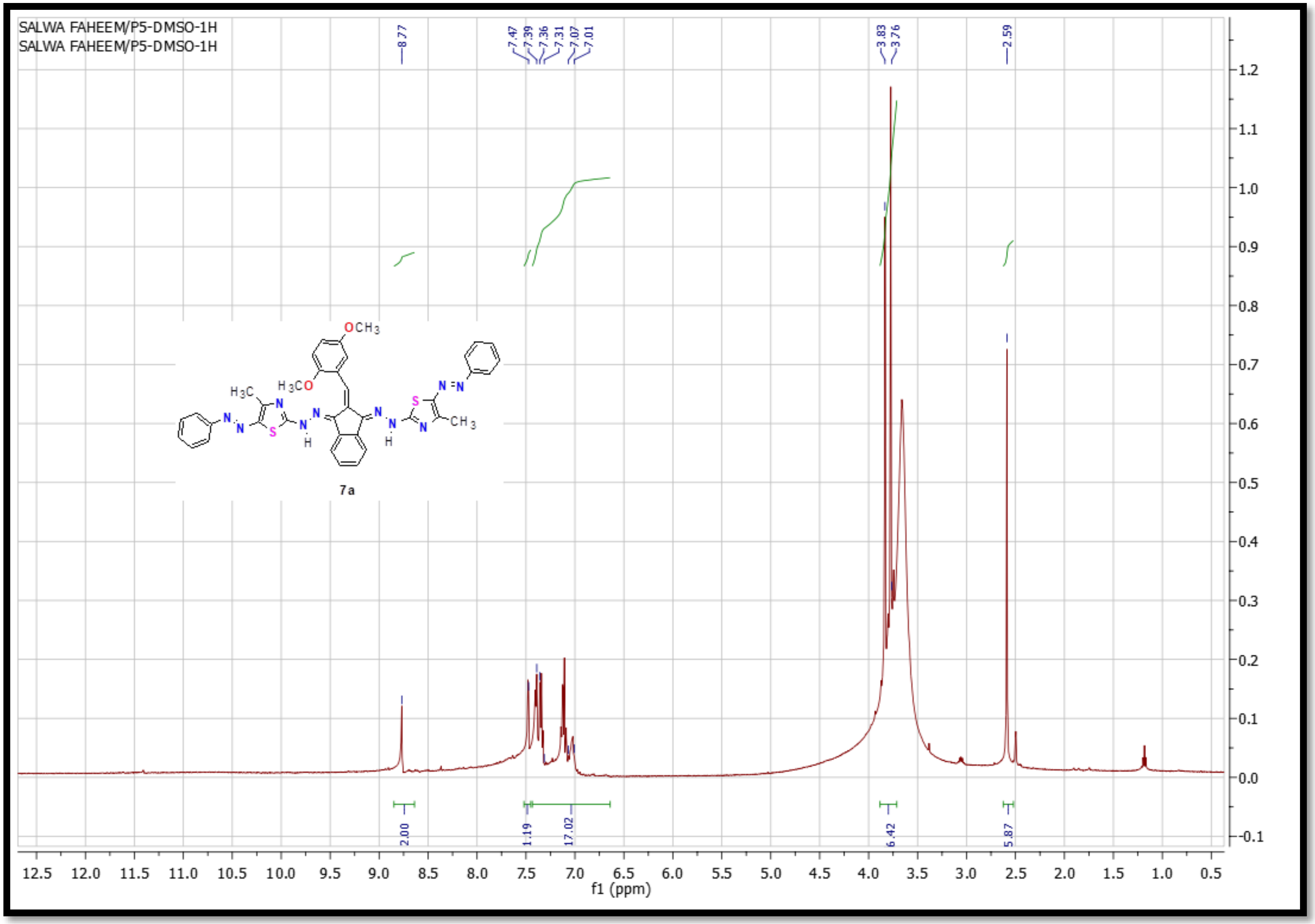

Figure S7. ^{1}H-NMR spectrum of compound (**7a**)

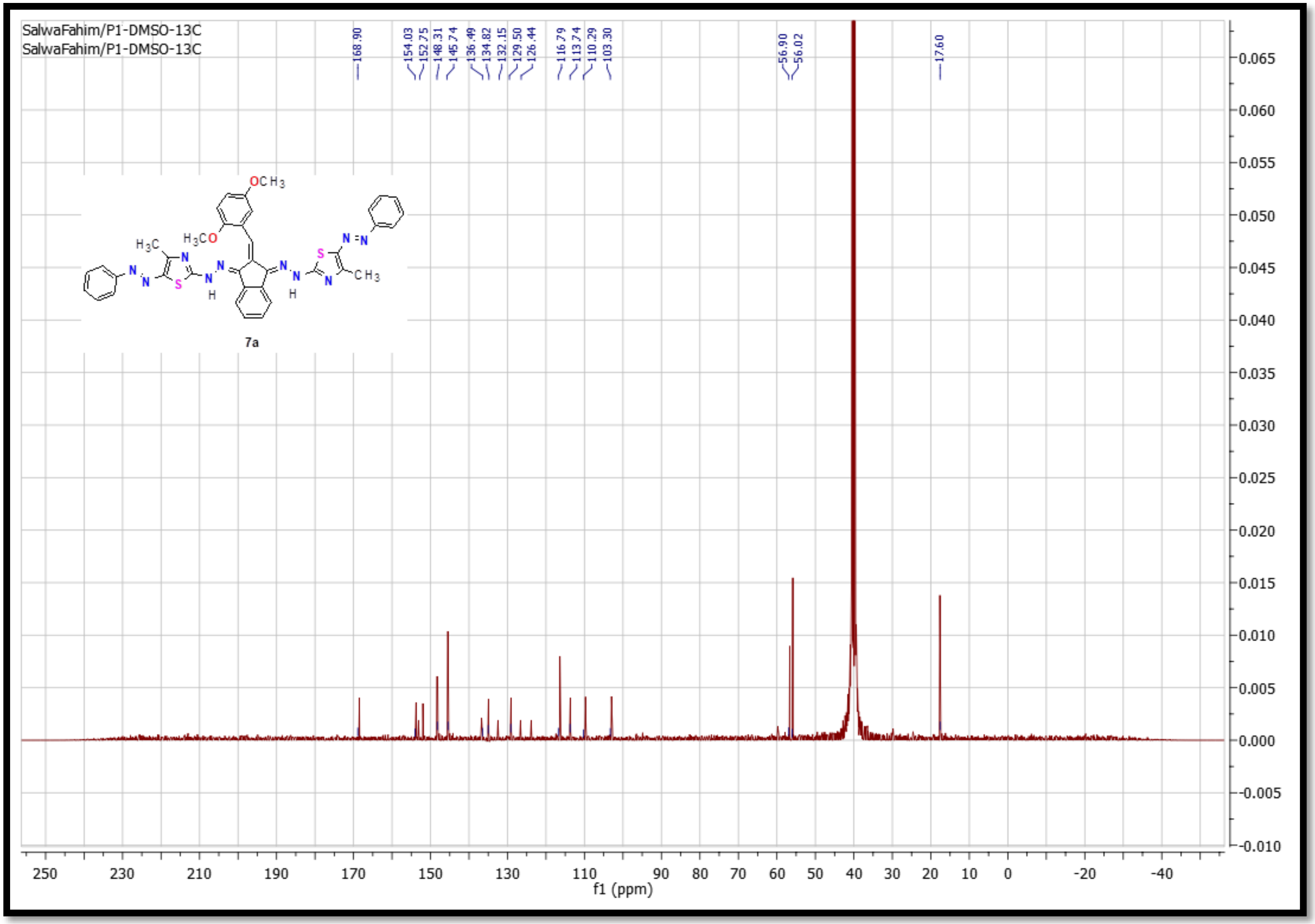

Figure S8. ^{13}C-NMR spectrum of compound (**7a**)

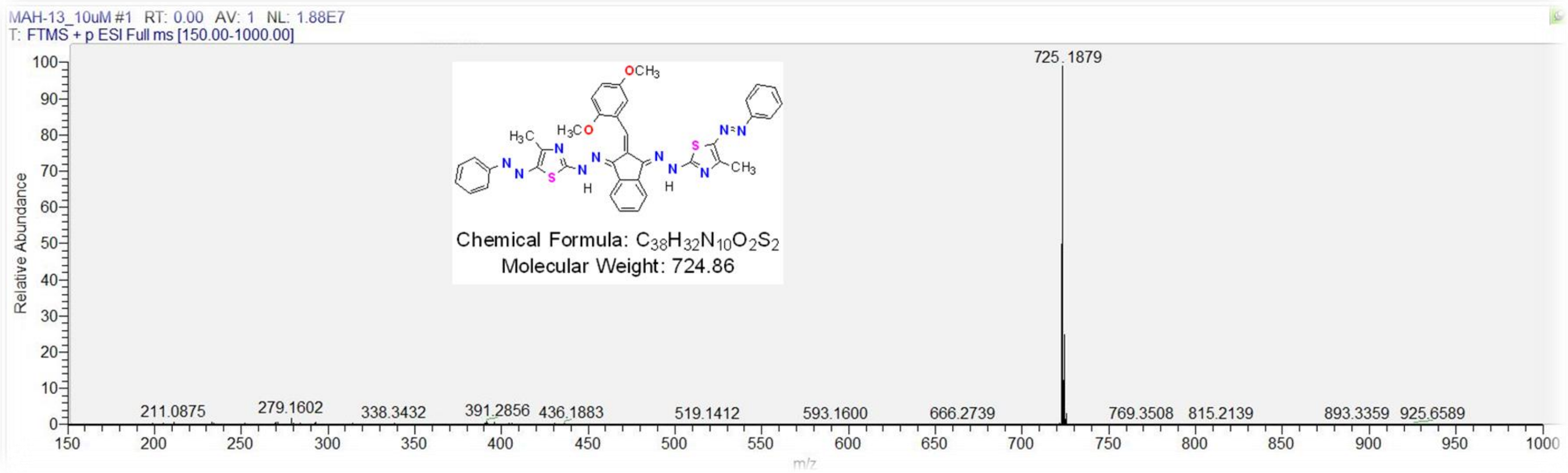

Figure S9. Mass spectrum of compound (**7a**)

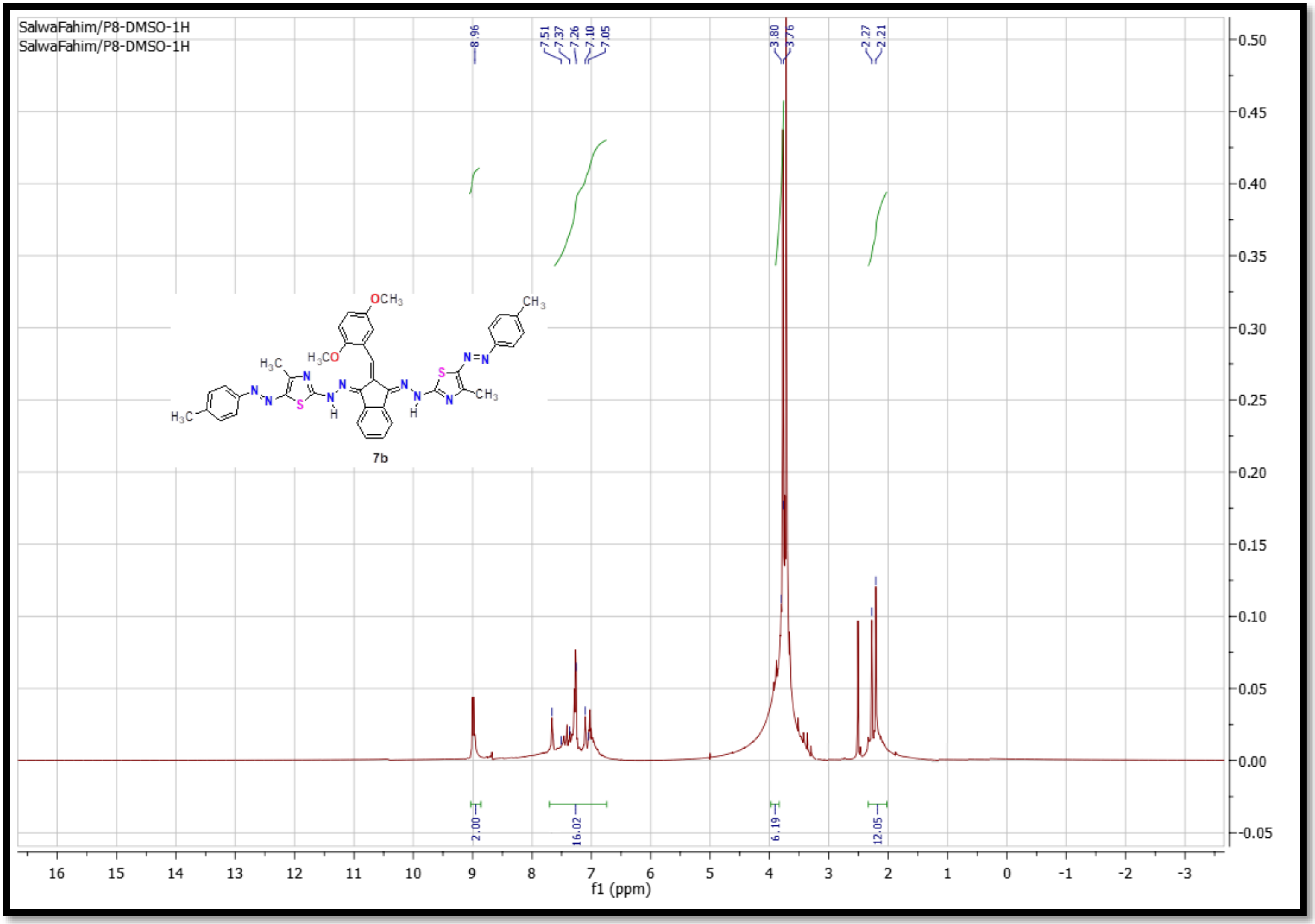

Figure S10. ^{1}H-NMR spectrum of compound (**7b**)

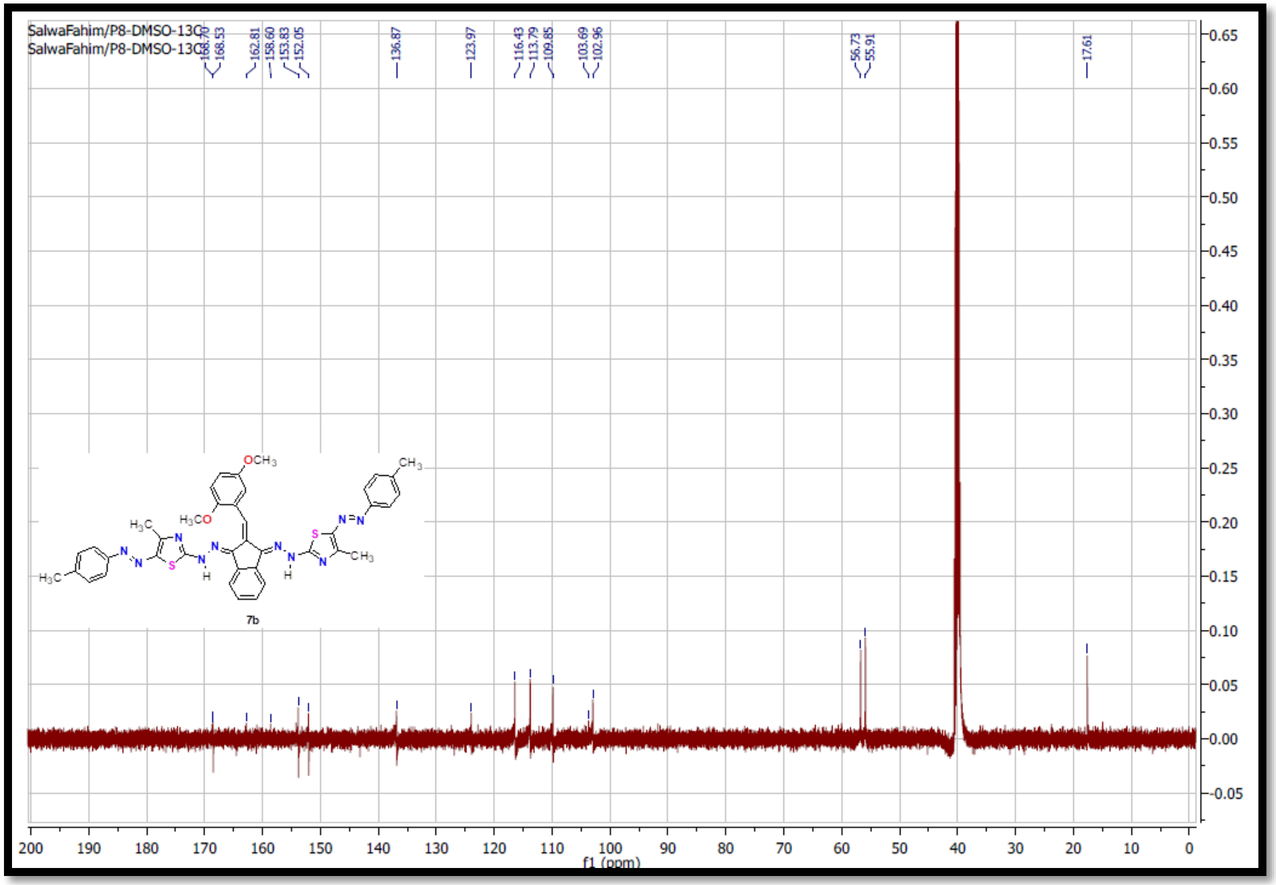

Figure S11. ^{13}C-NMR spectrum of compound (**7b**)

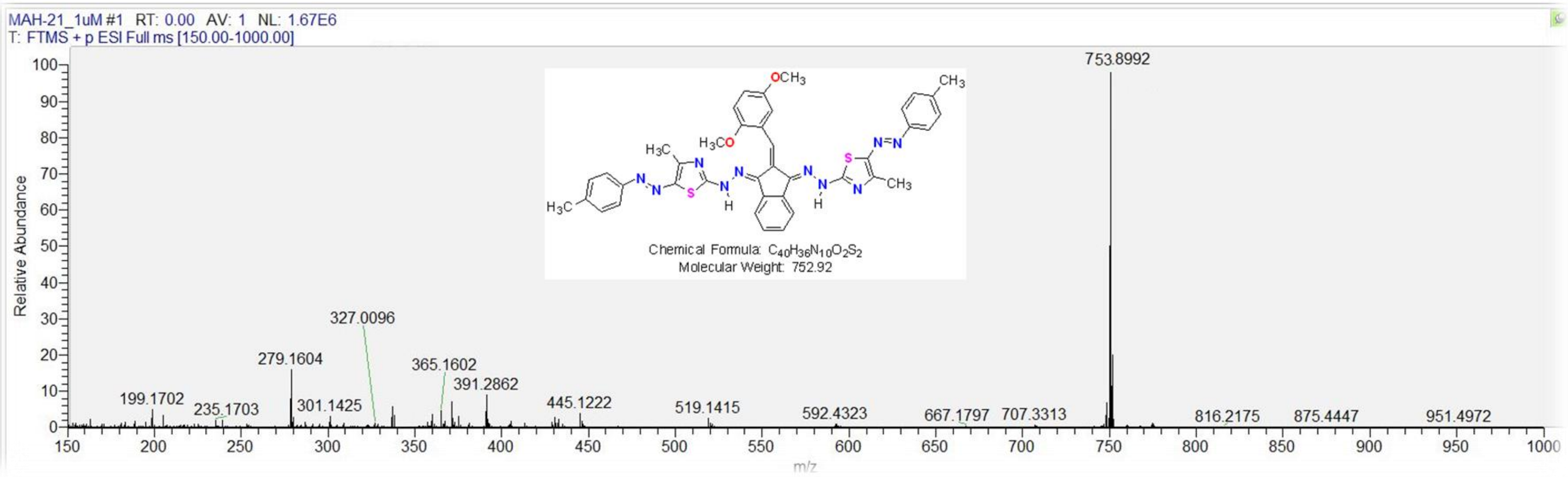

Figure S12. Mass spectrum of compound (**7b**)

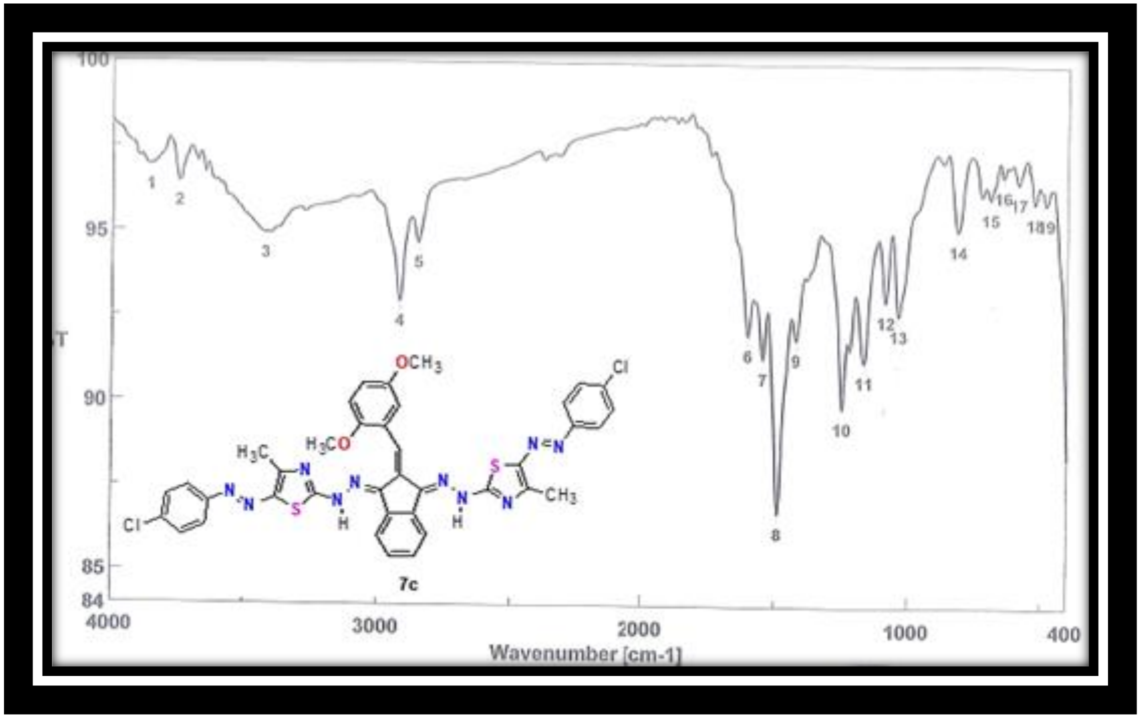

Figure S13. IR spectrum of compound (**7c**)

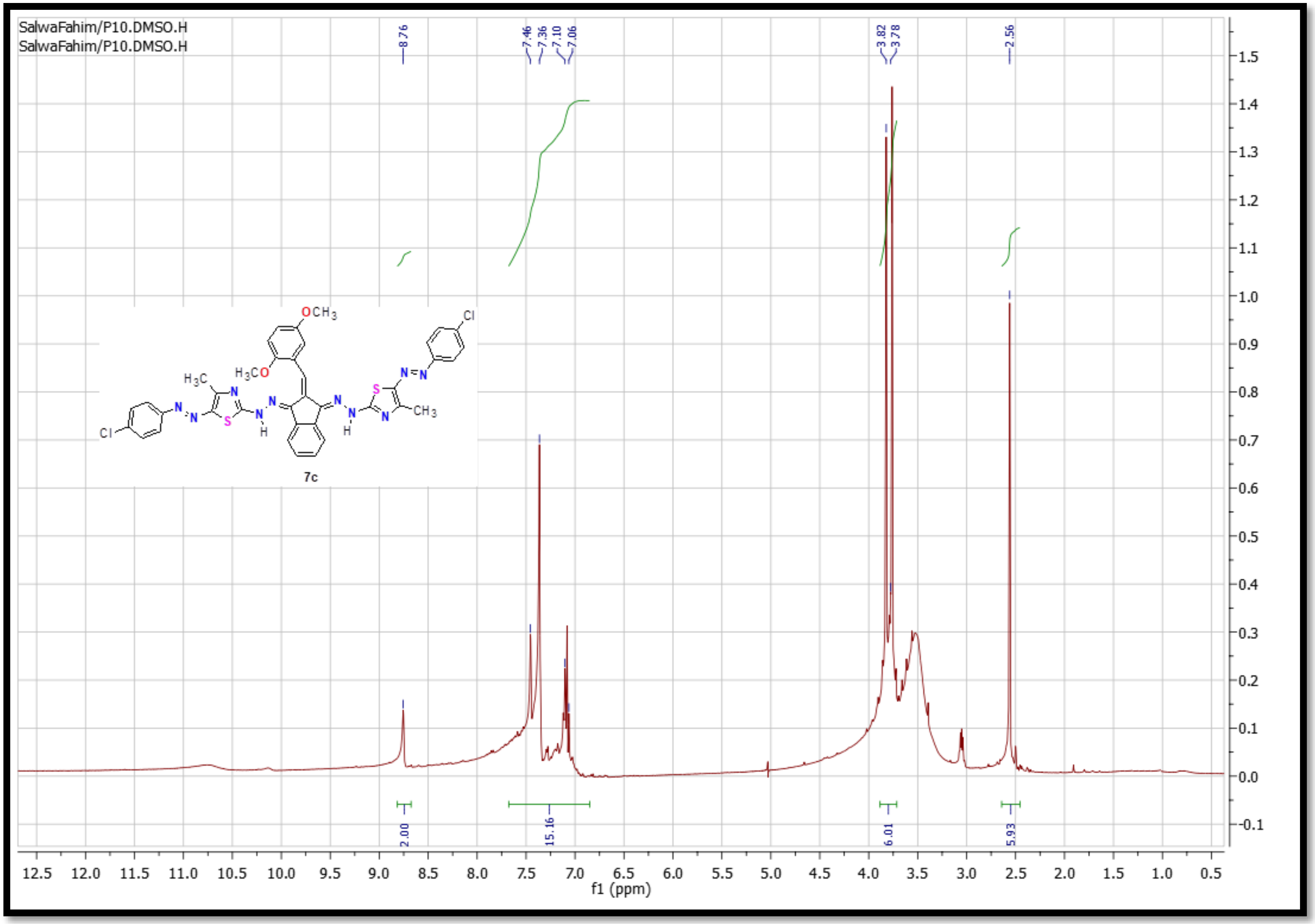

Figure S14. ^{1}H-NMR spectrum of compound (**7c**)

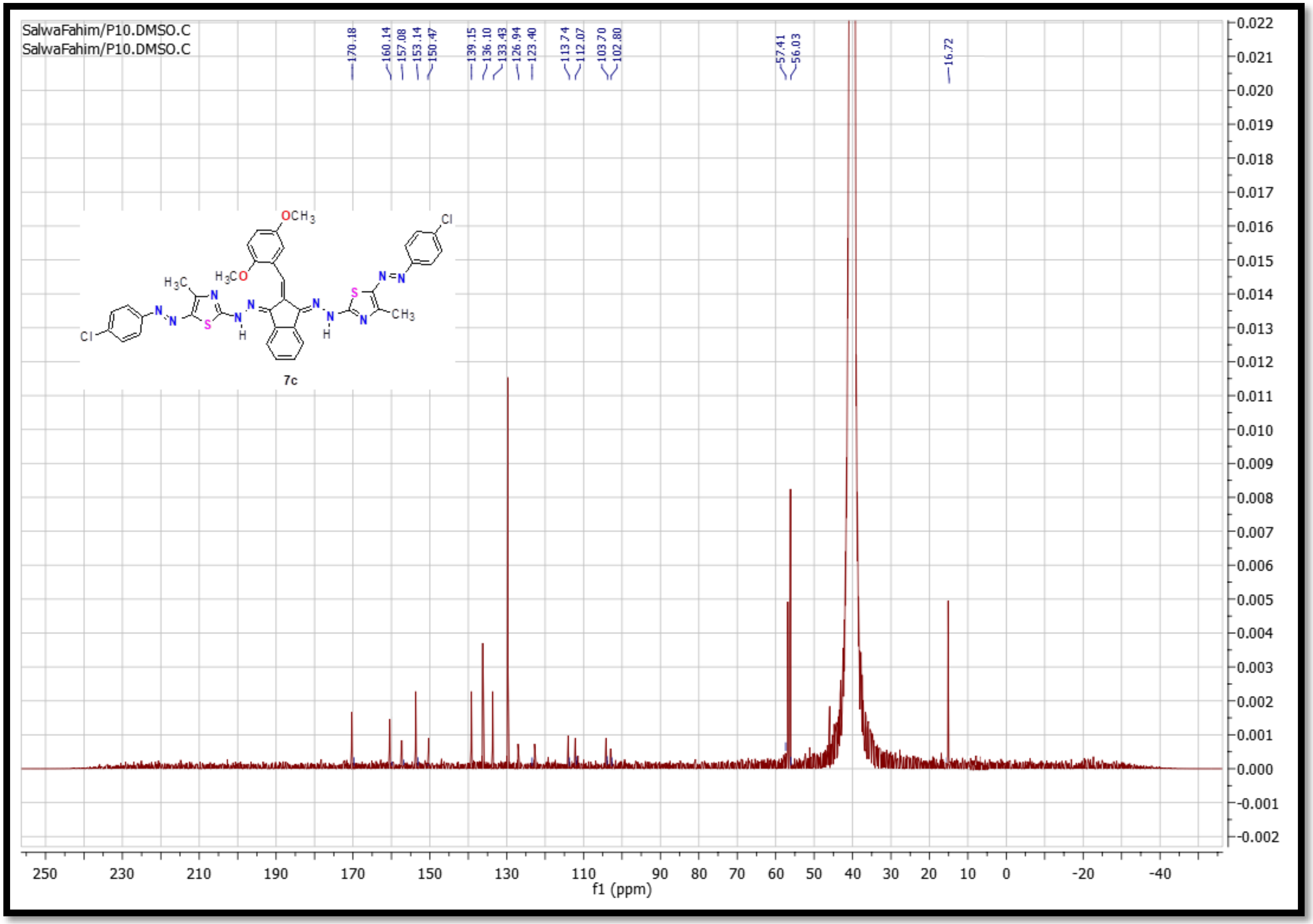

Figure S15. ^{13}C-NMR spectrum of compound (**7c**)

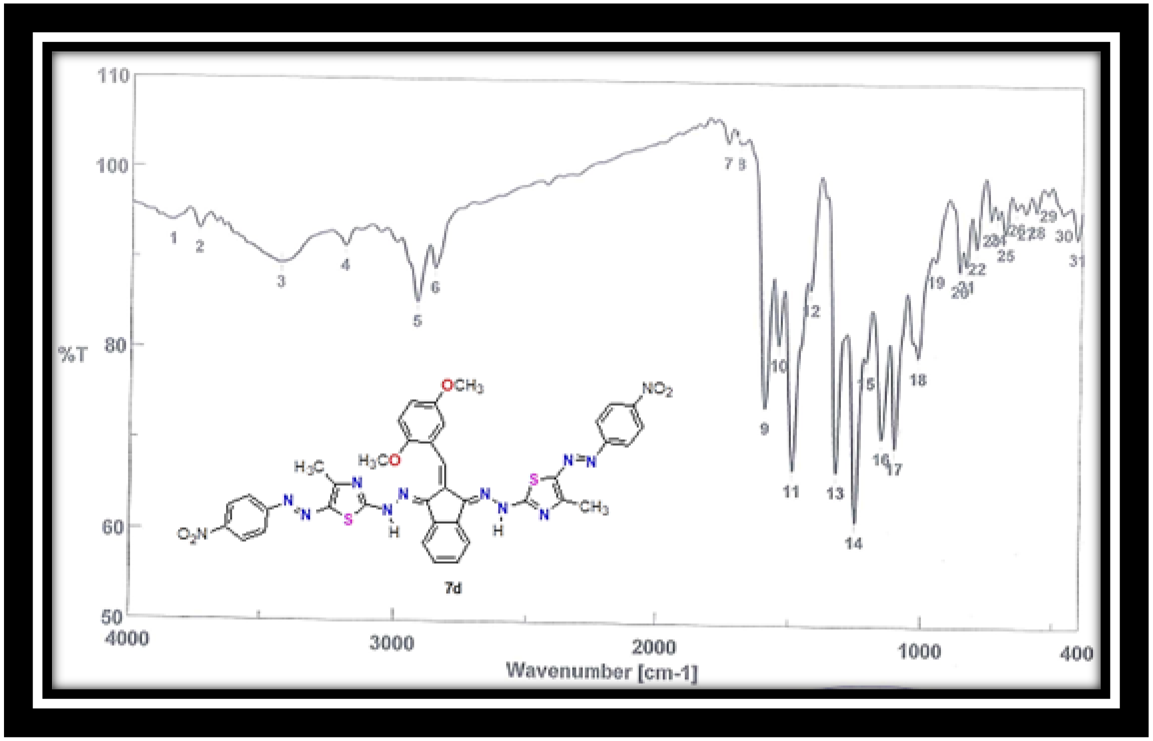

Figure S16. IR spectrum of compound (**7d**)

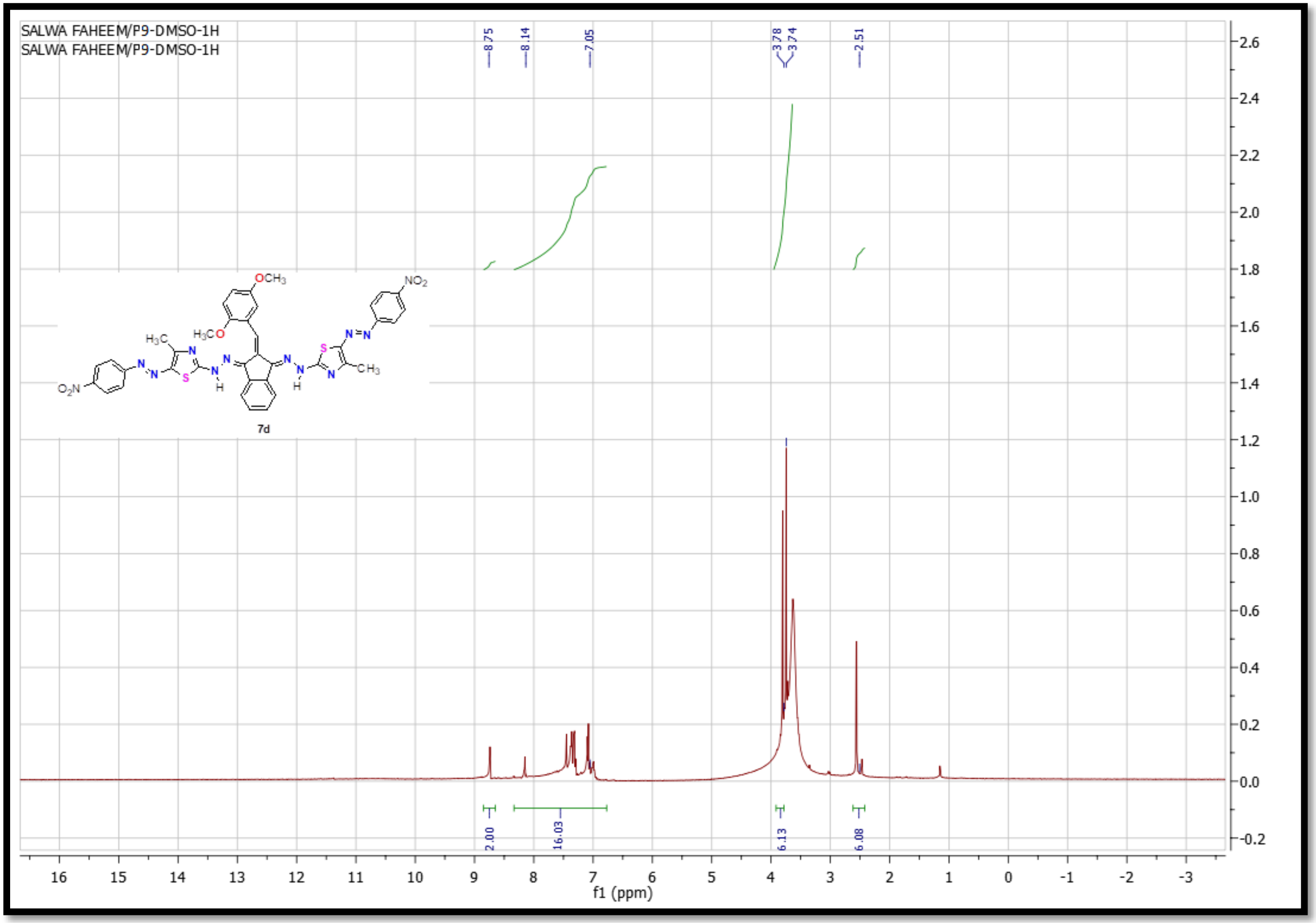

Figure S17. ^{1}H-NMR spectrum of compound (**7d**)

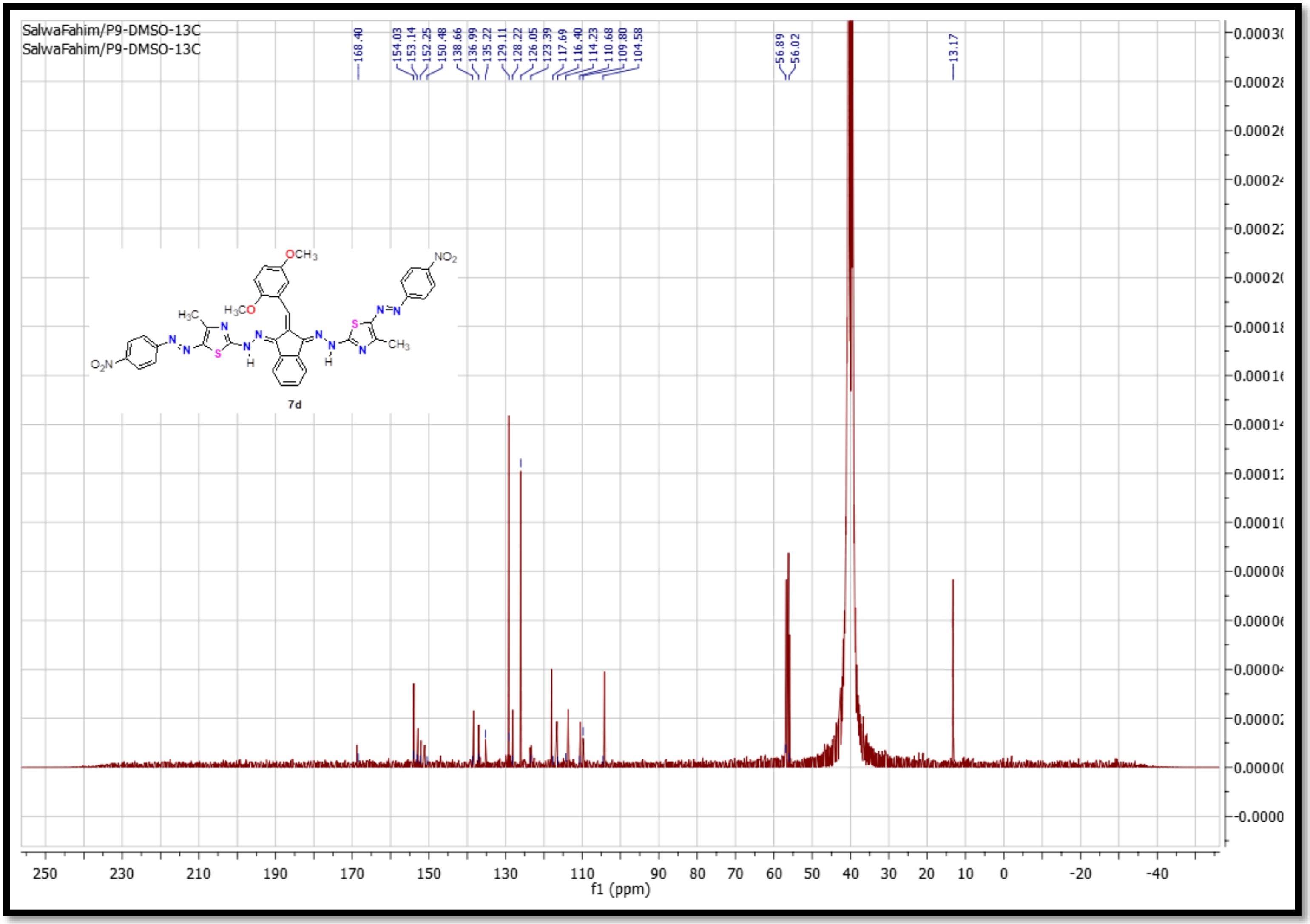

Figure S18. ^{13}C-NMR spectrum of compound (**7d**)

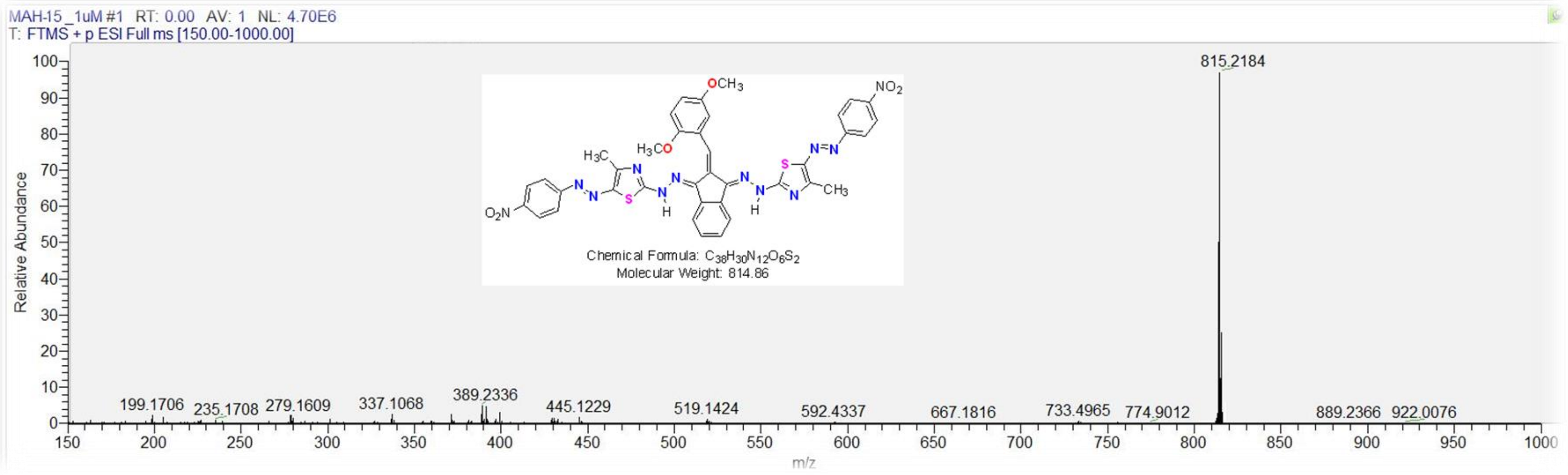

Figure S19. Mass spectrum of compound (**7d**)

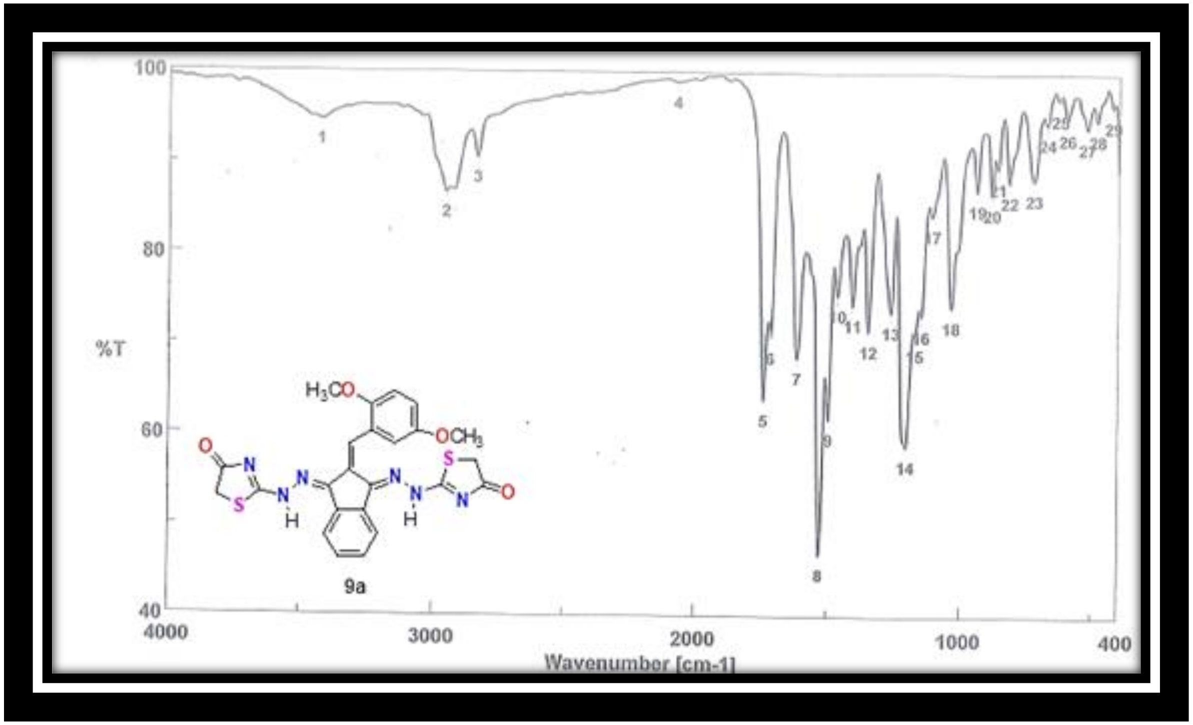

Figure S20. IR spectrum of compound (**9a**)

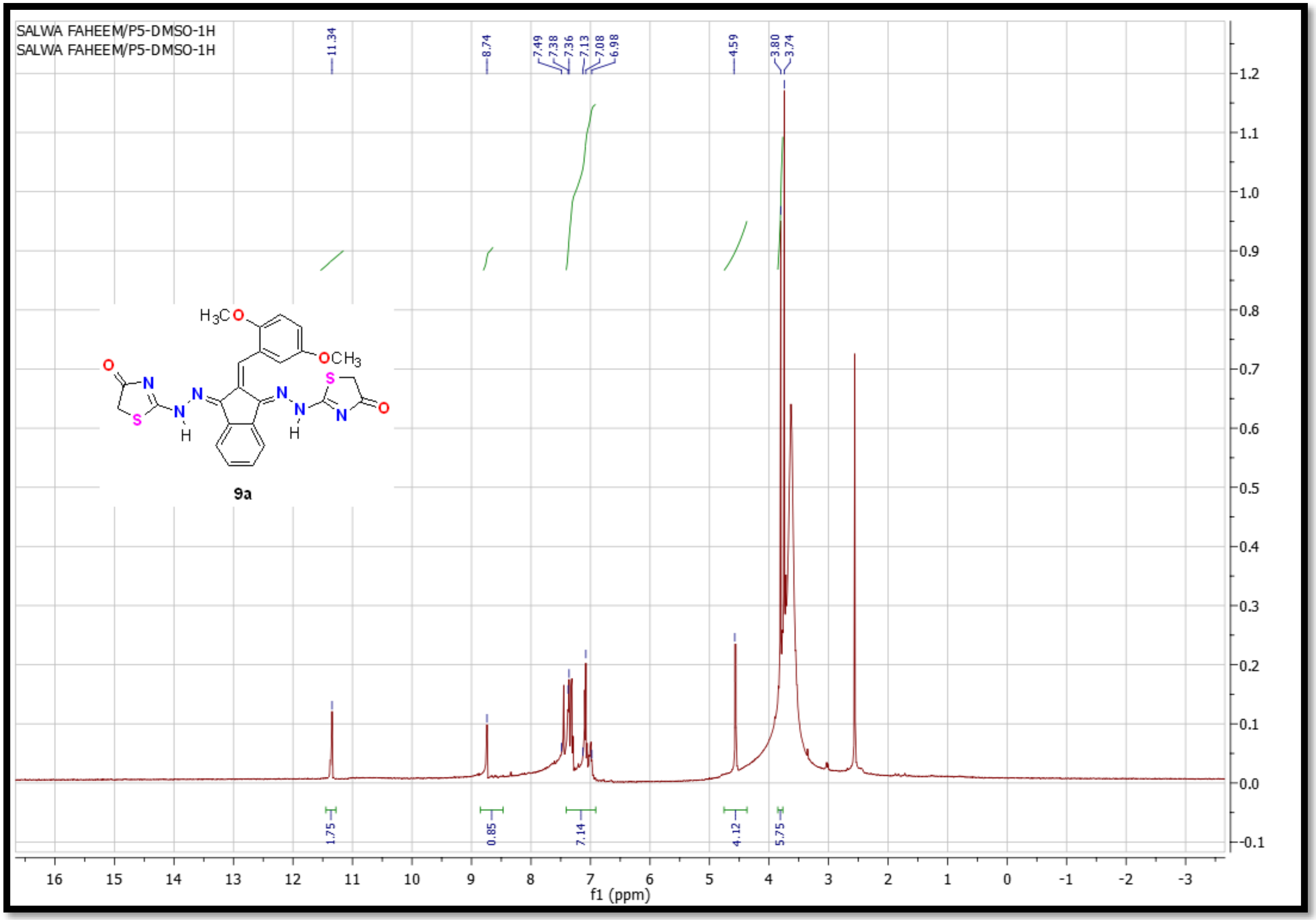

Figure S21. ^{1}H-NMR spectrum of compound (**9a**)

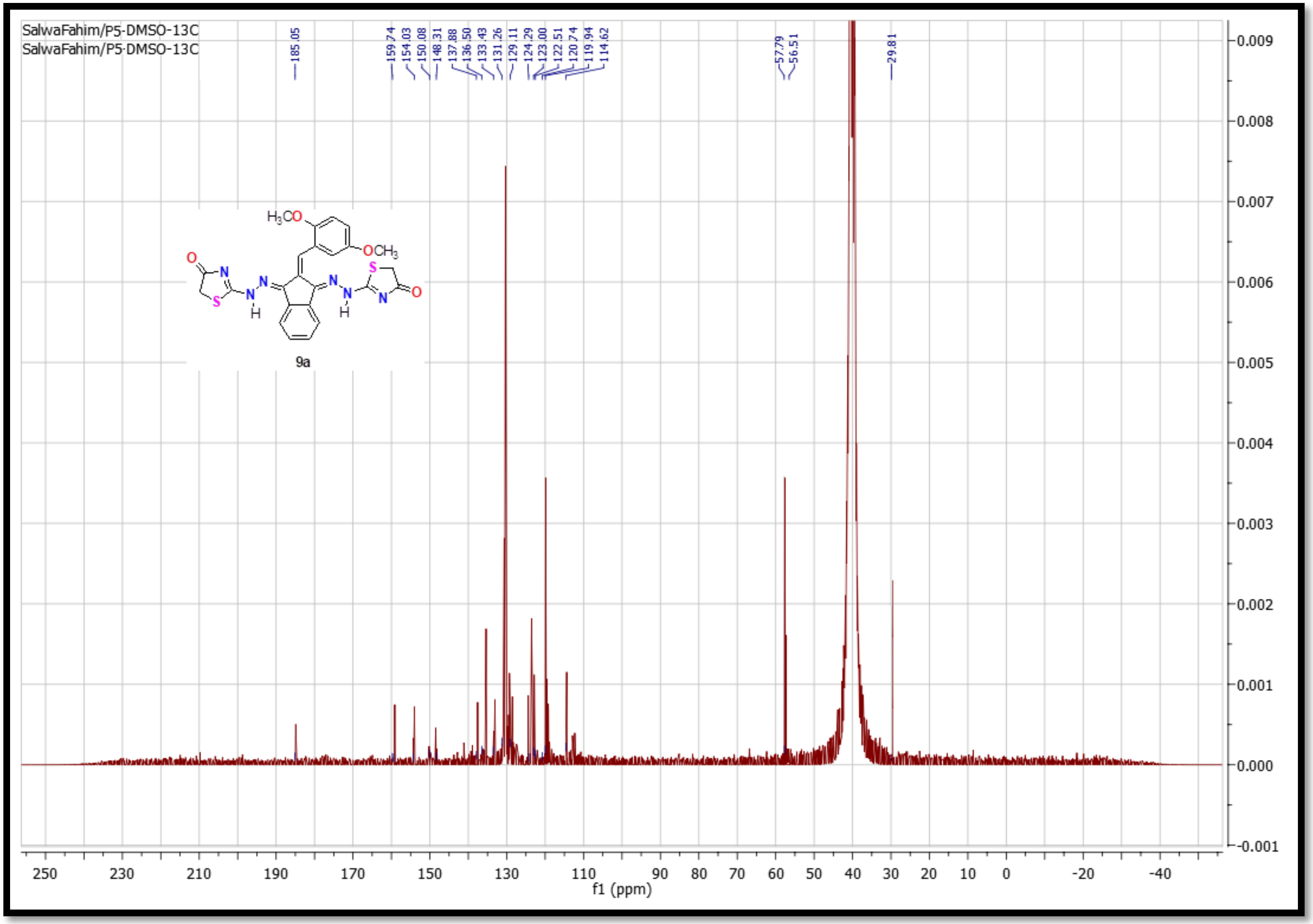

Figure S22. ^{13}C-NMR spectrum of compound (**9a**)

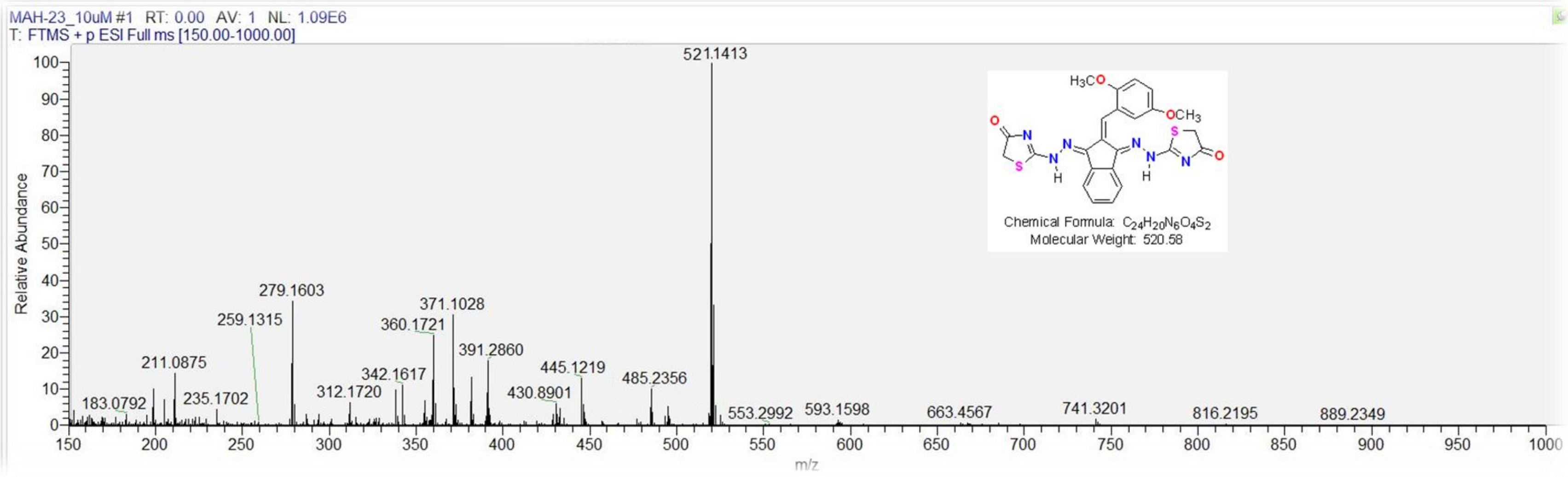

Figure S23. Mass spectrum of compound (**9a**)

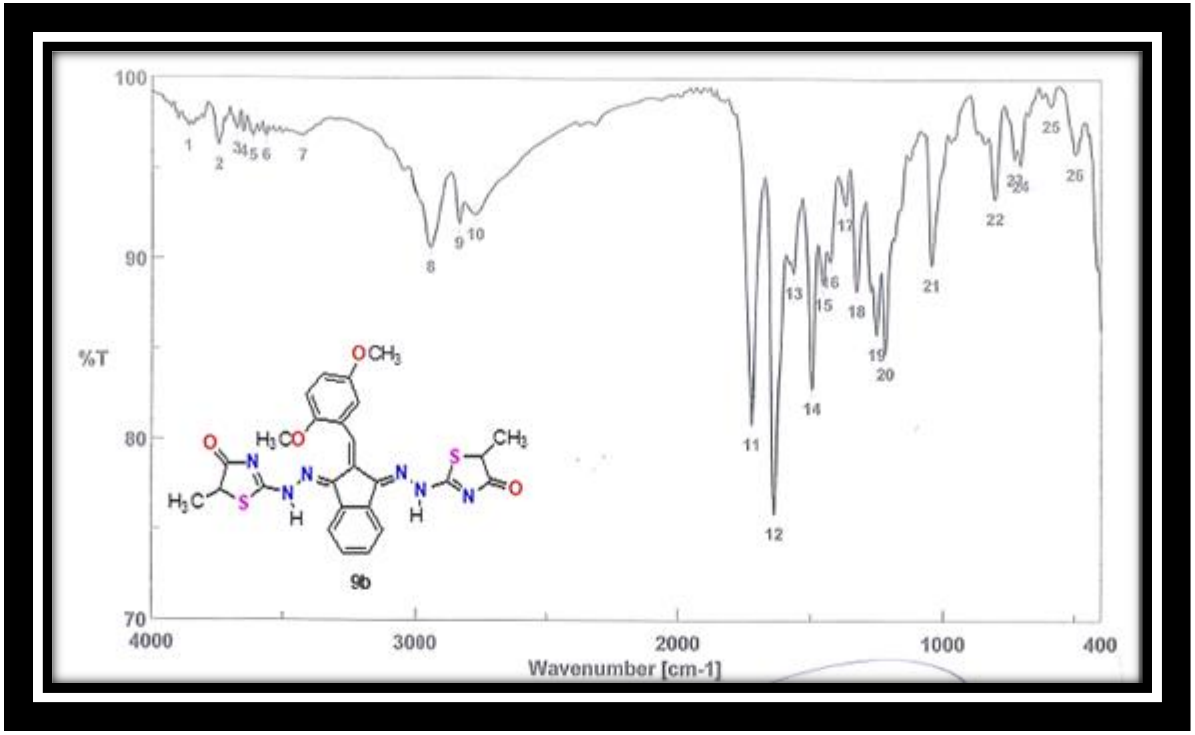

Figure S24. IR spectrum of compound (**9b**)

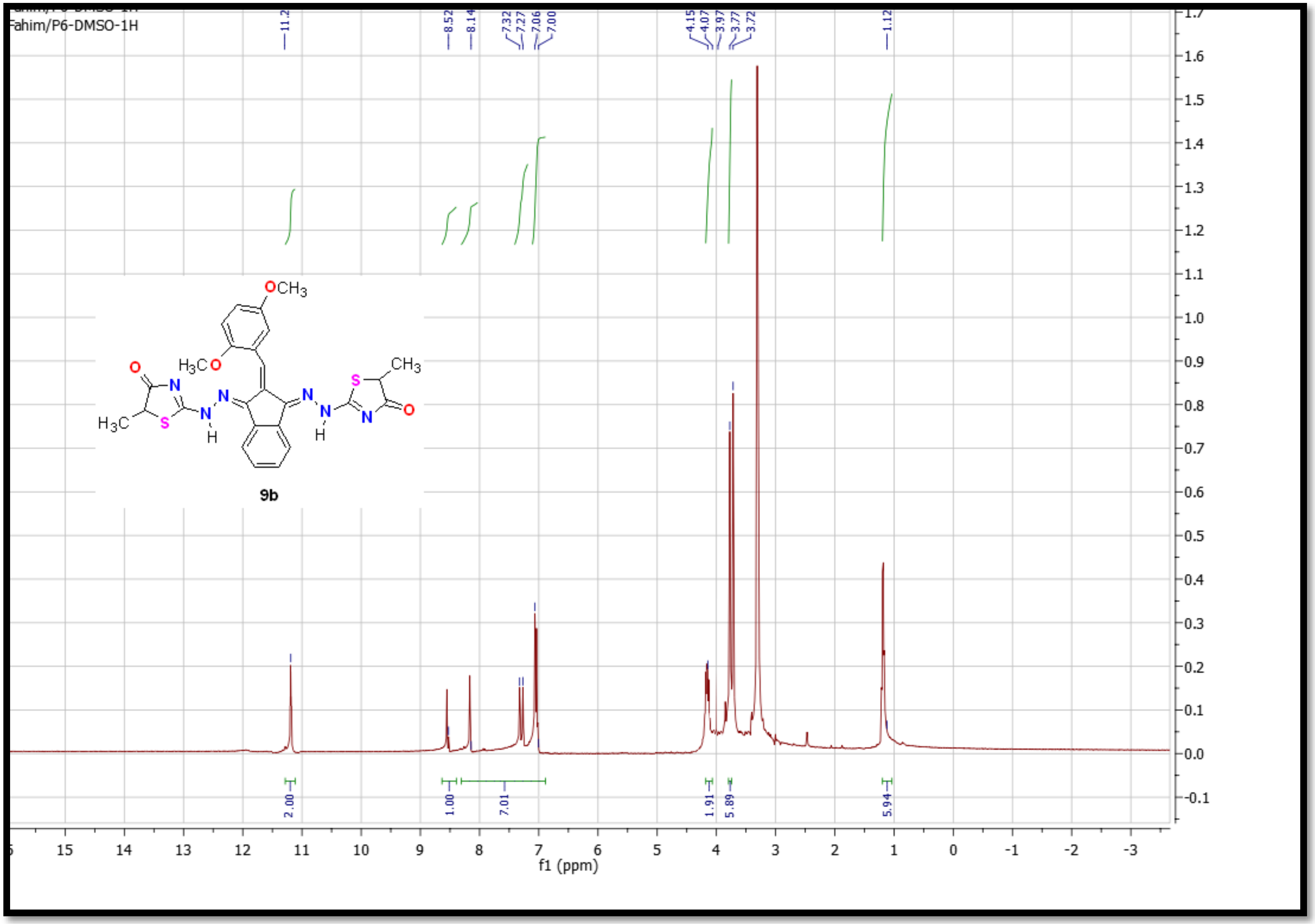

Figure S25. ^{1}H-NMR spectrum of compound (**9b**)

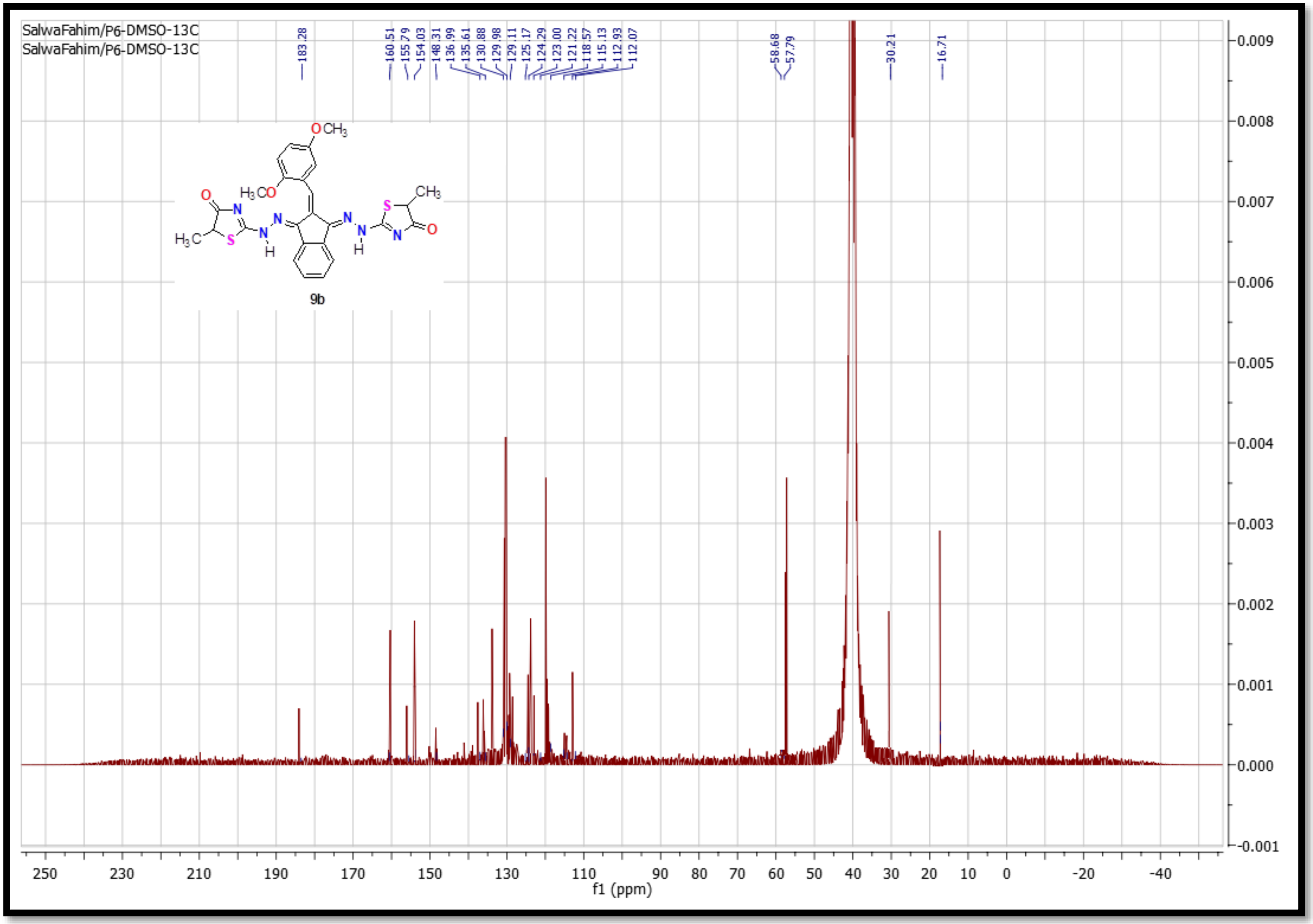

Figure S26. ^{13}C-NMR spectrum of compound (**9b**)

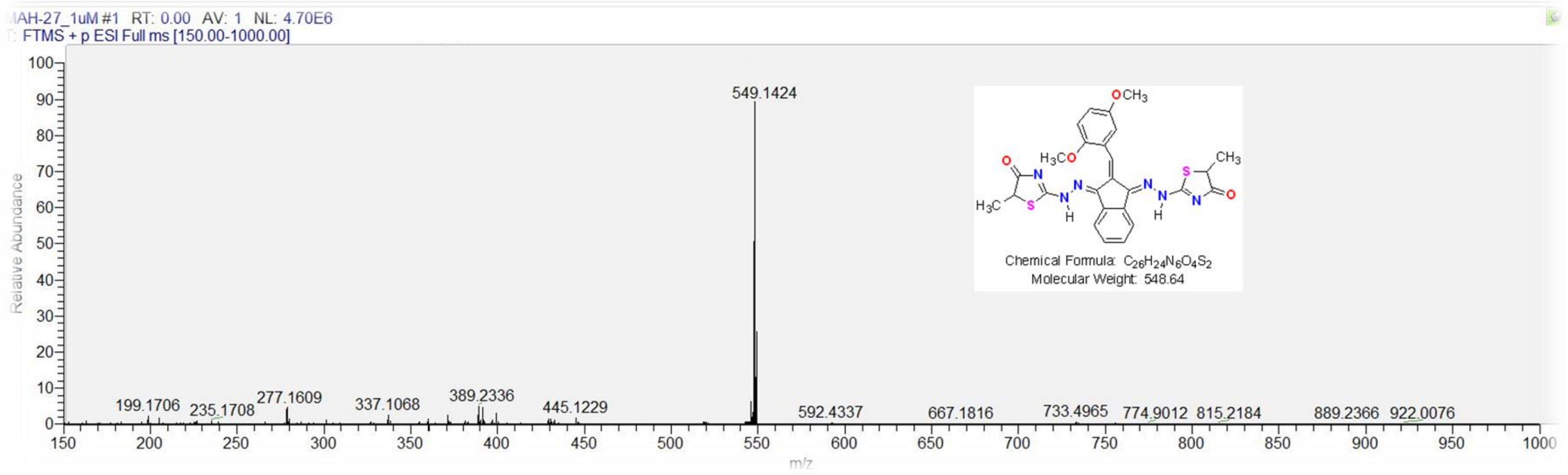

Figure S27. Mass spectrum of compound (**9b**)

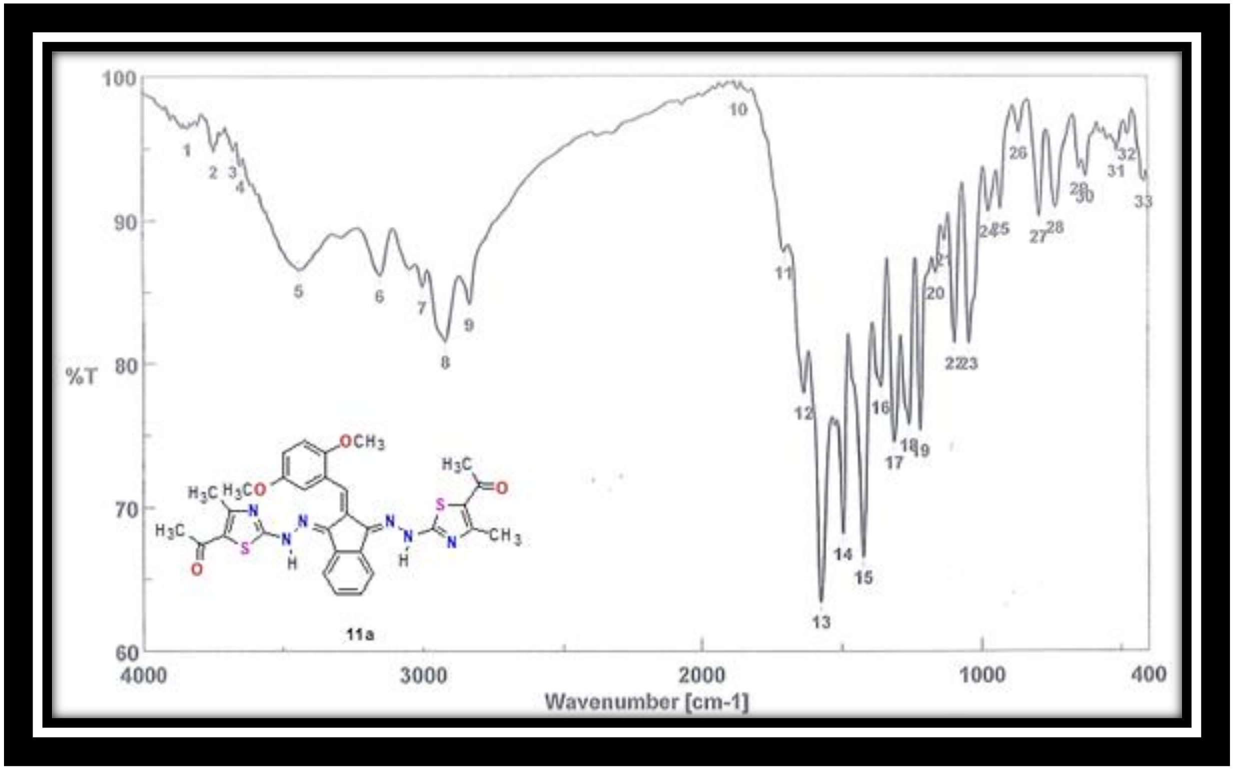

Figure S28. IR spectrum of compound (**11a**)

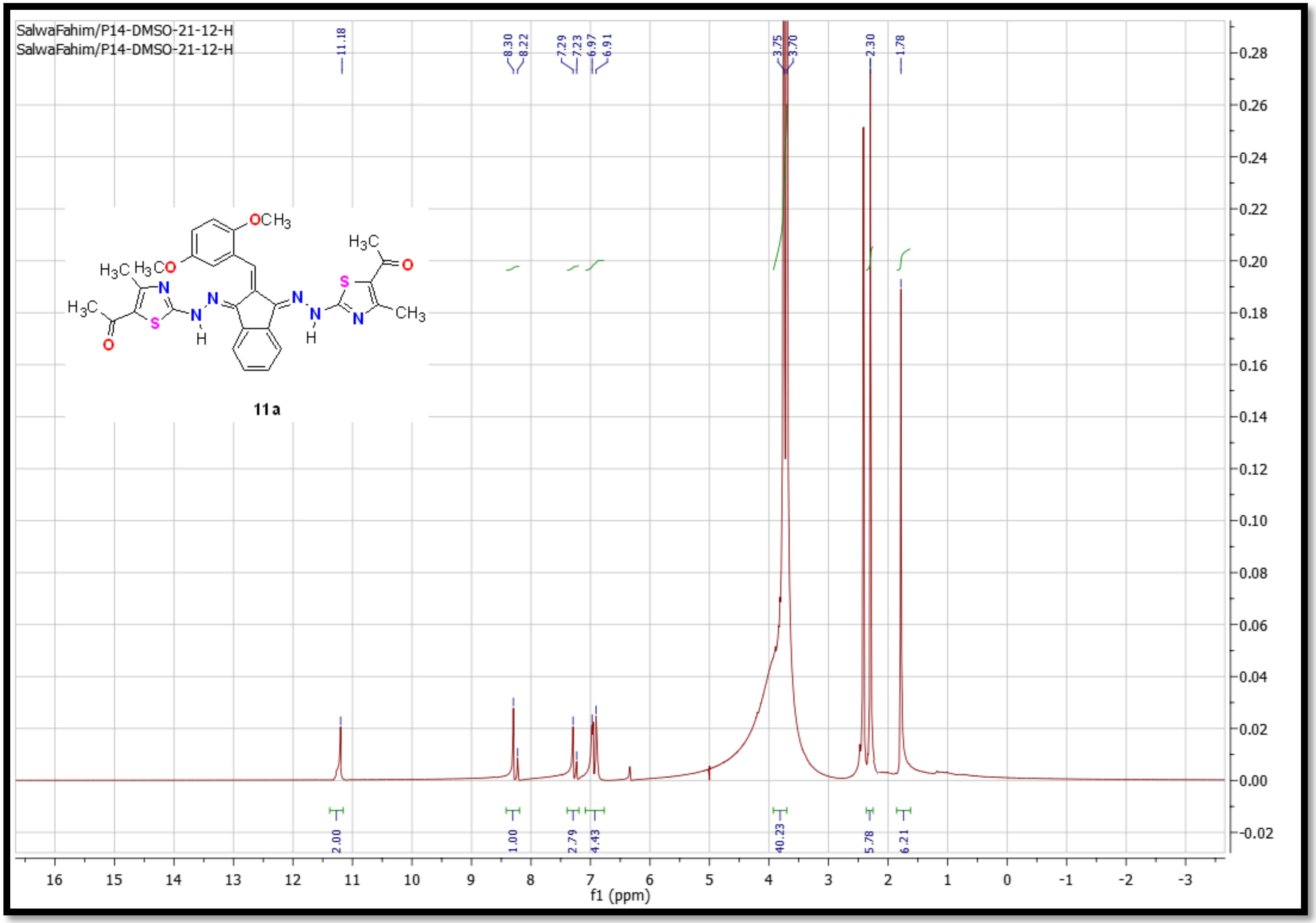

Figure S29. ^{1}H-NMR spectrum of compound (**11a**)

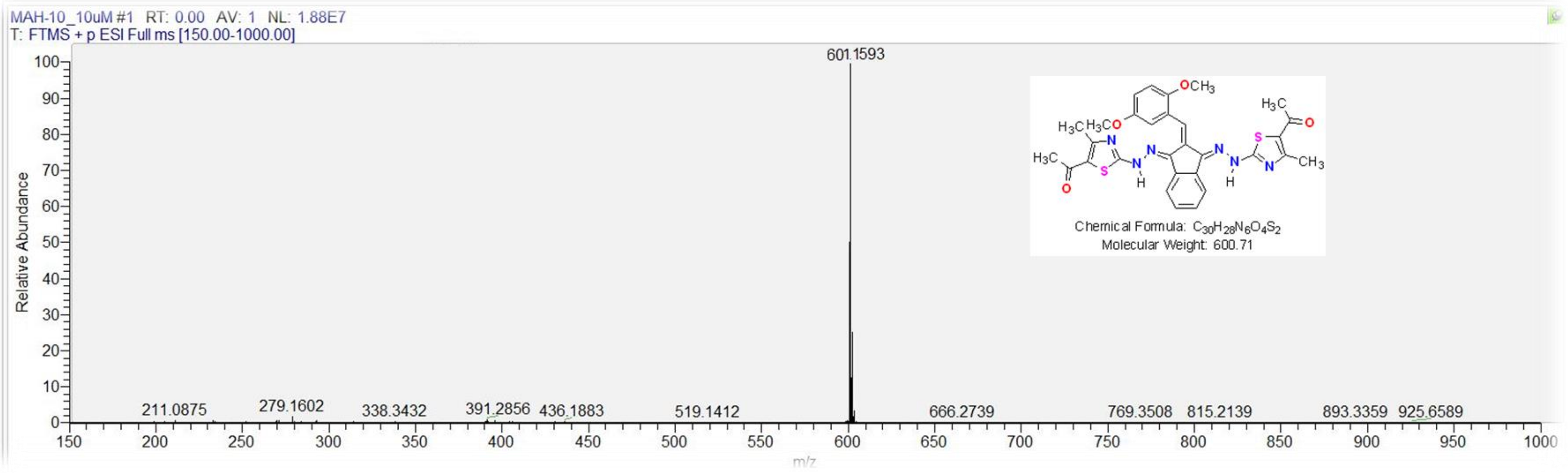

Figure S30. Mass spectrum of compound (**11a**)

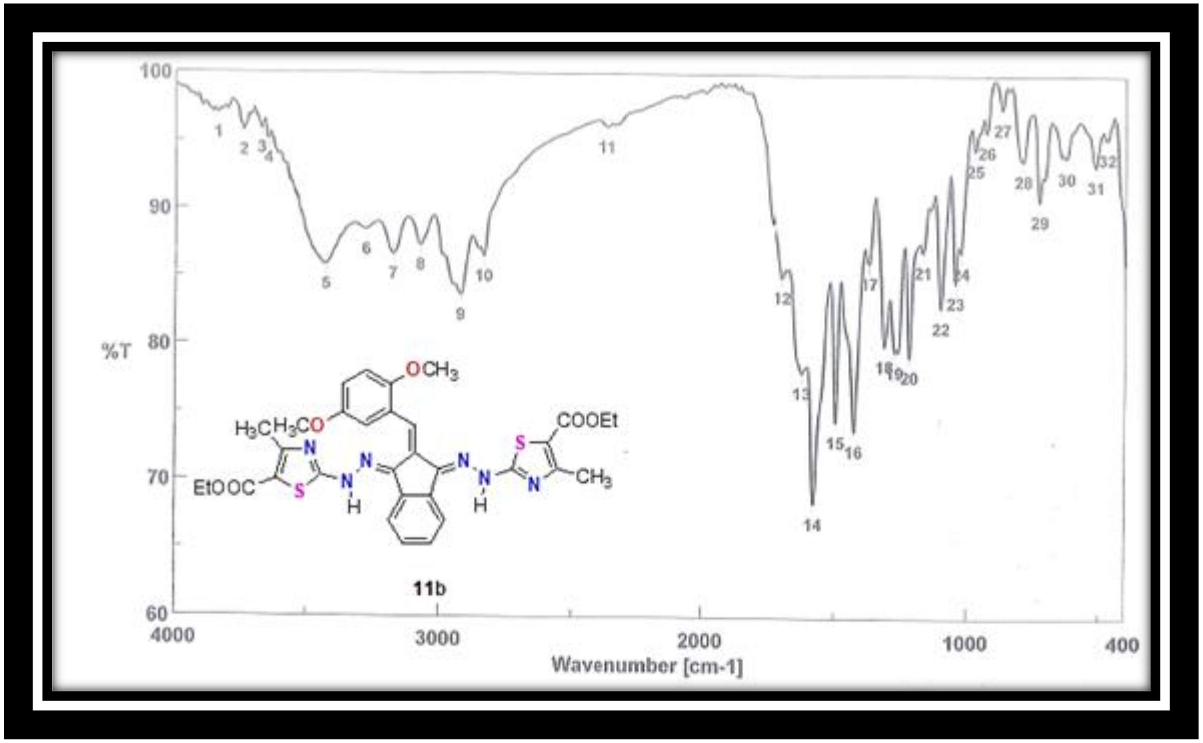

Figure S31. IR spectrum of compound (**11b**)

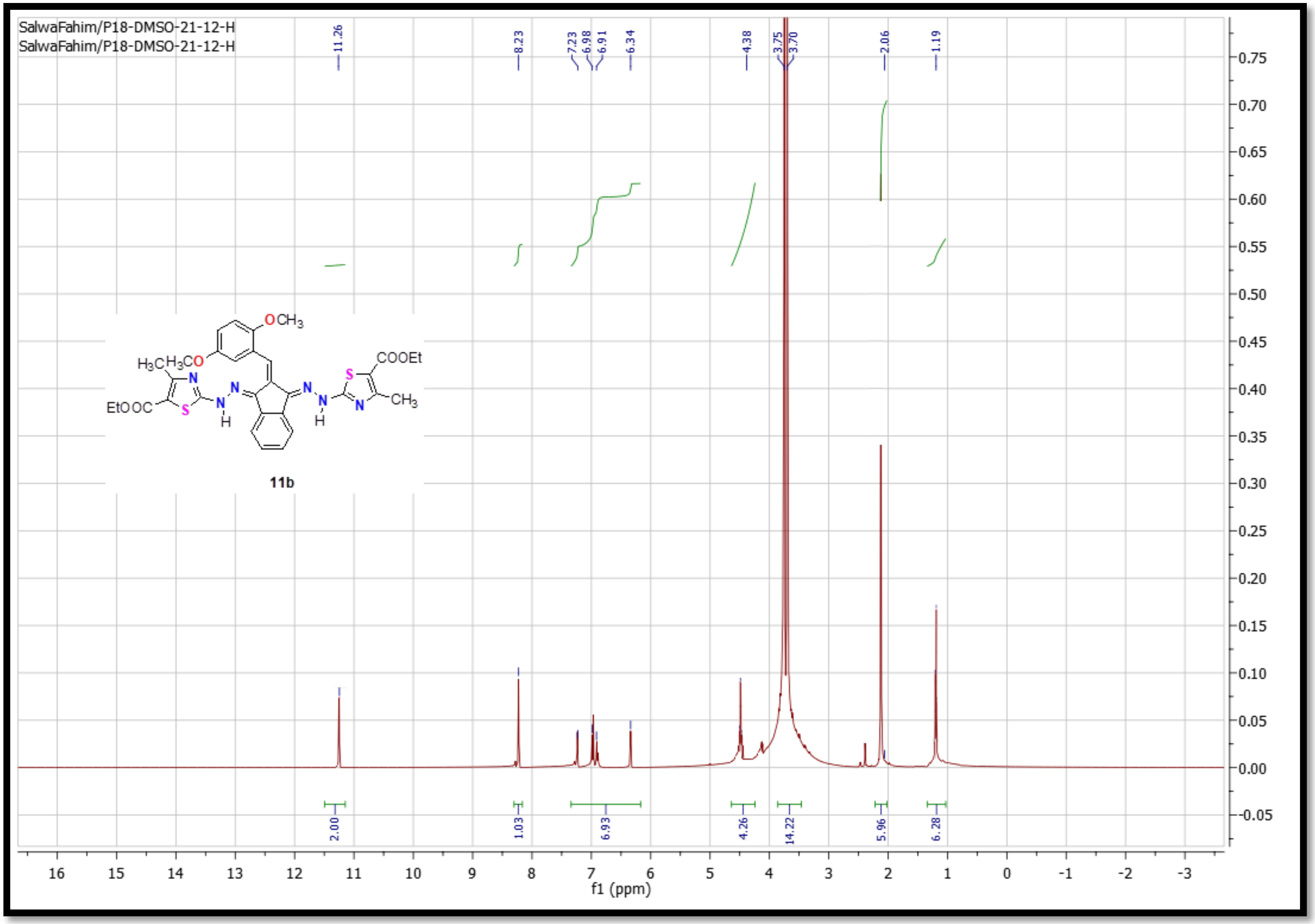

Figure S32. ^{1}H-NMR spectrum of compound (**11b**)

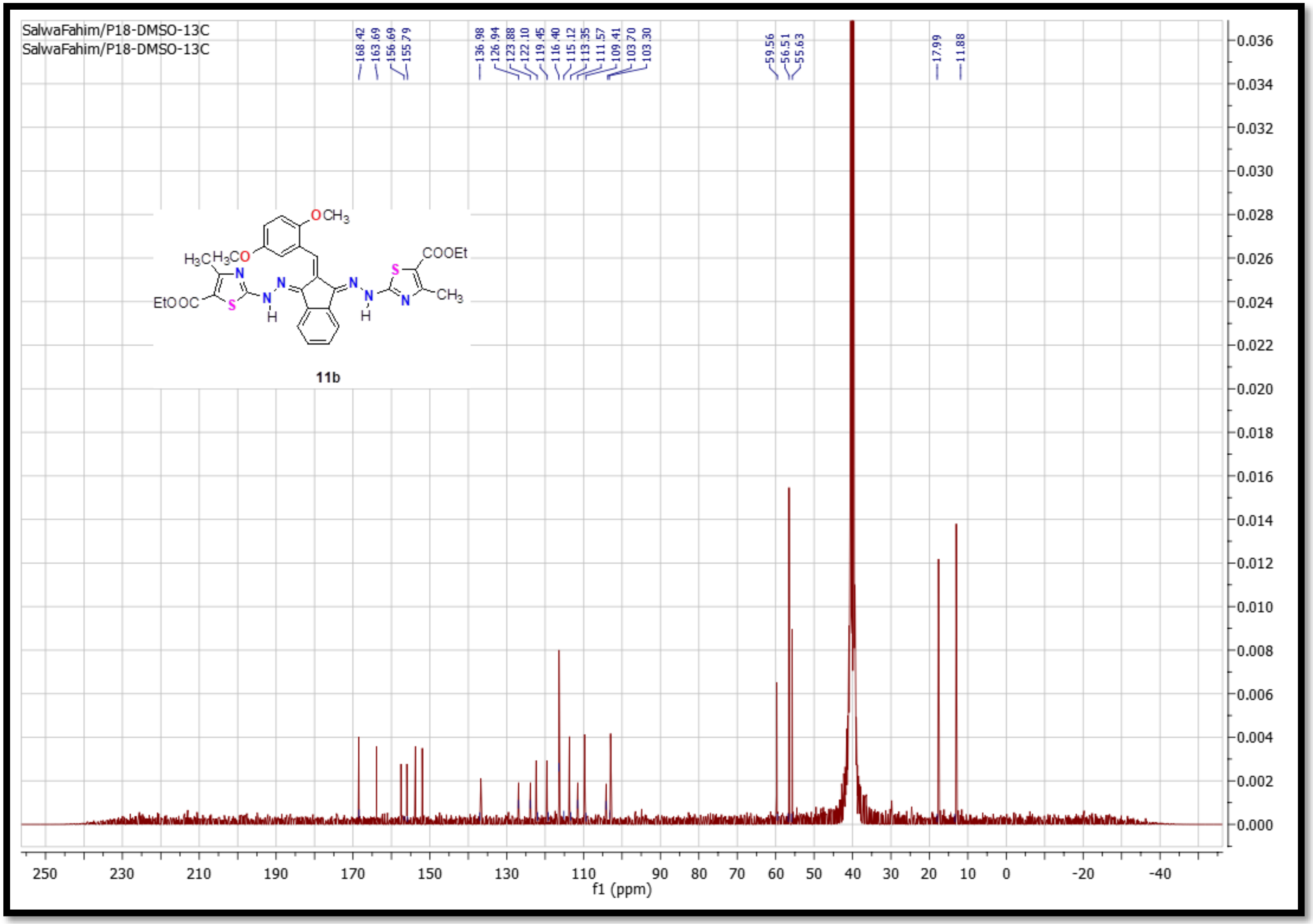

Figure S33. ^{13}C-NMR spectrum of compound (**11b**)

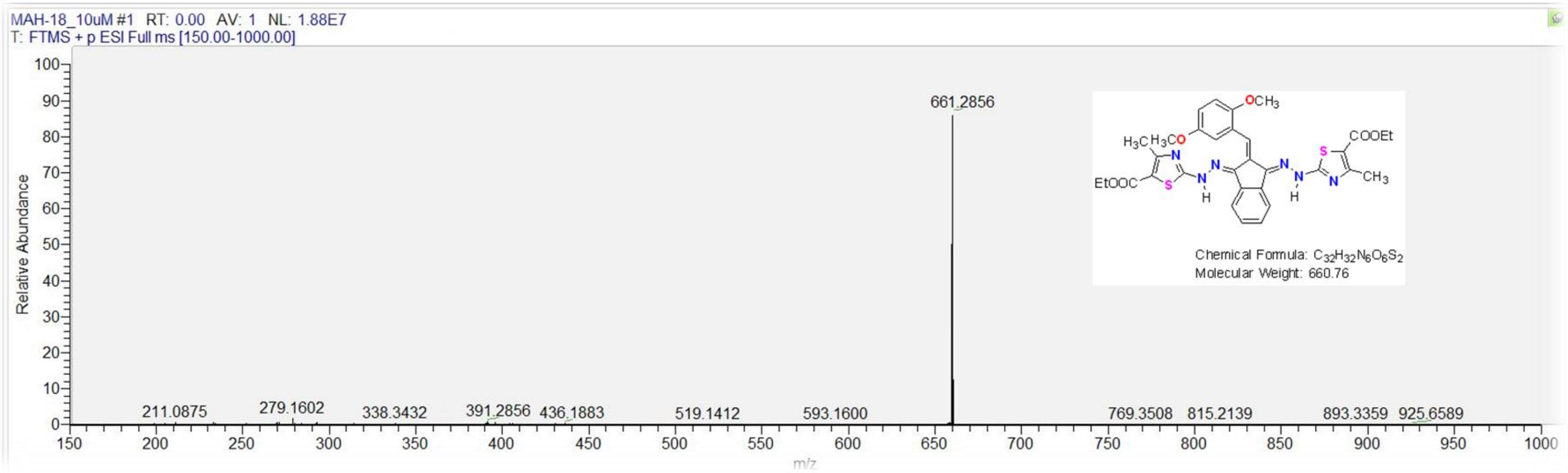

Figure S34. Mass spectrum of compound (**11b**)

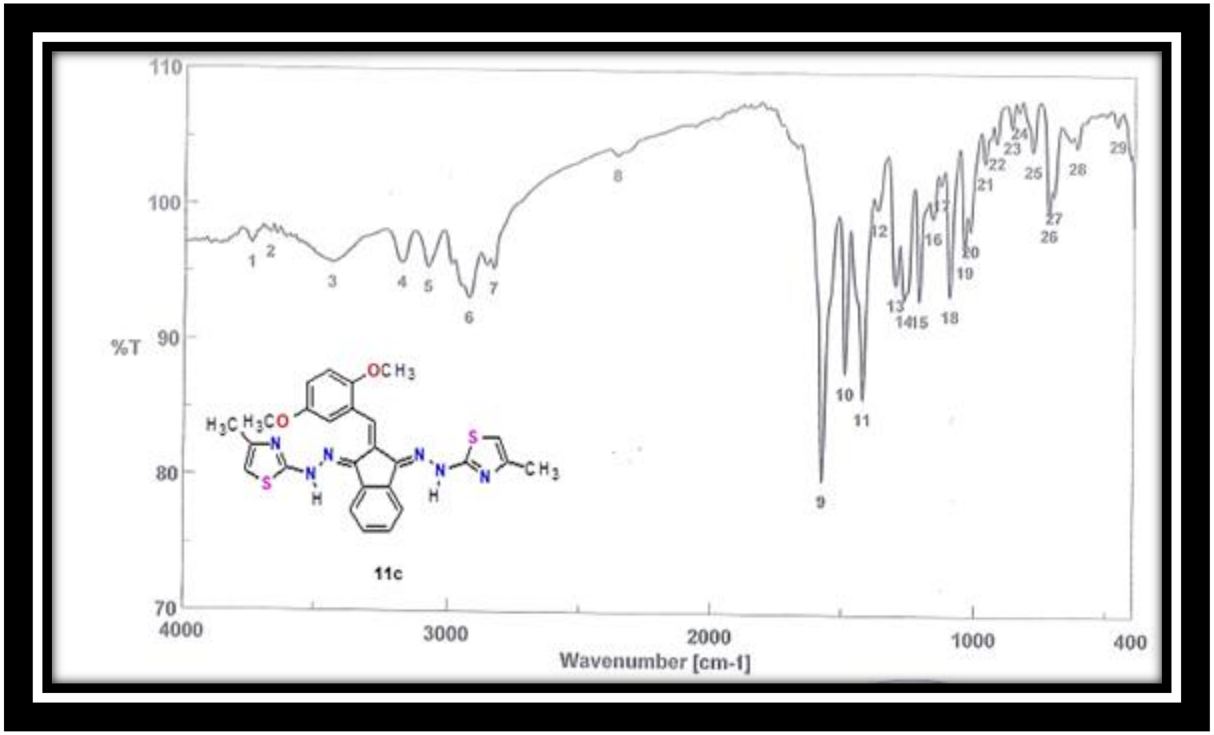

Figure S35. IR spectrum of compound (**11c**)

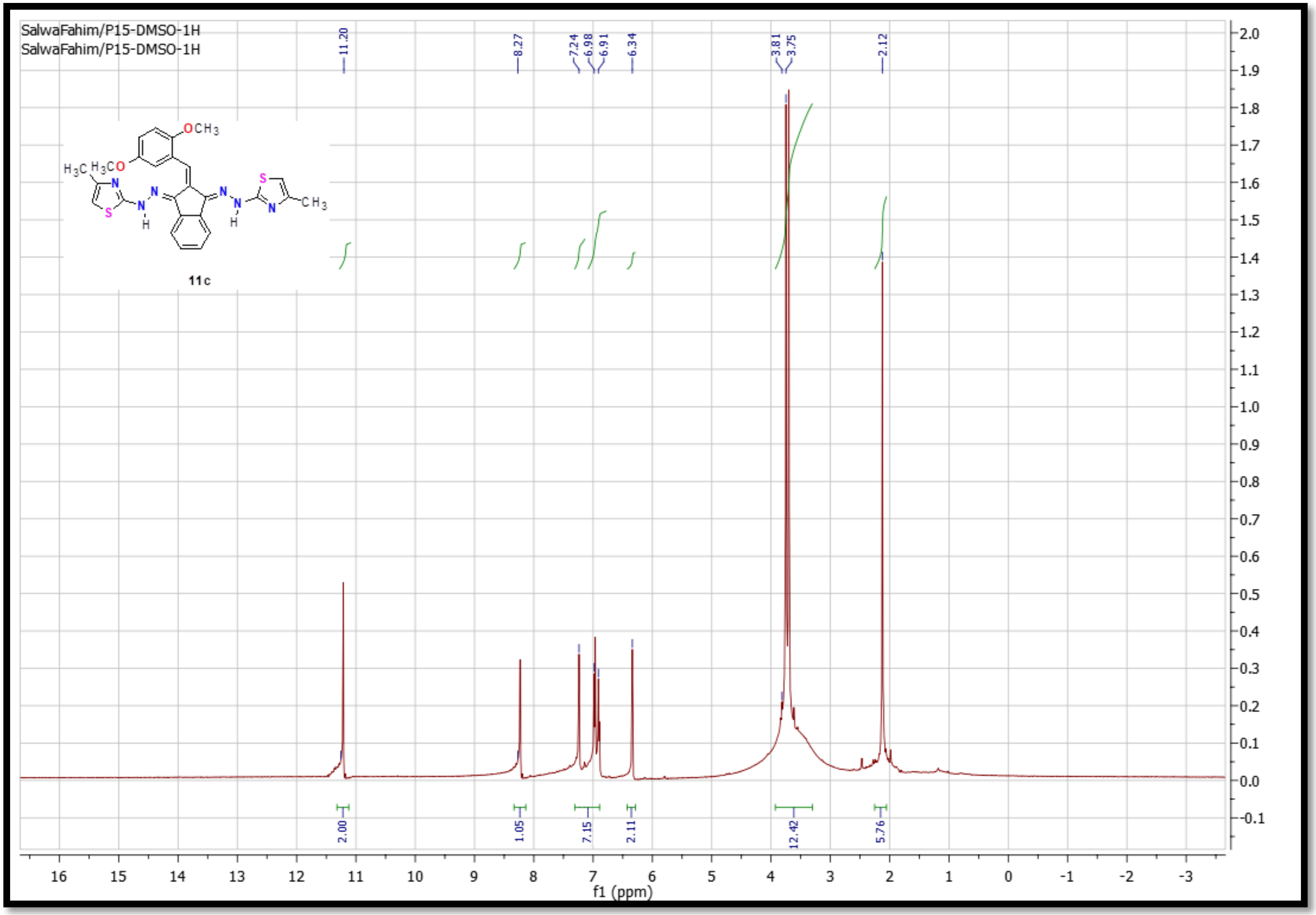

Figure S36. ^{1}H-NMR spectrum of compound (**11c**)

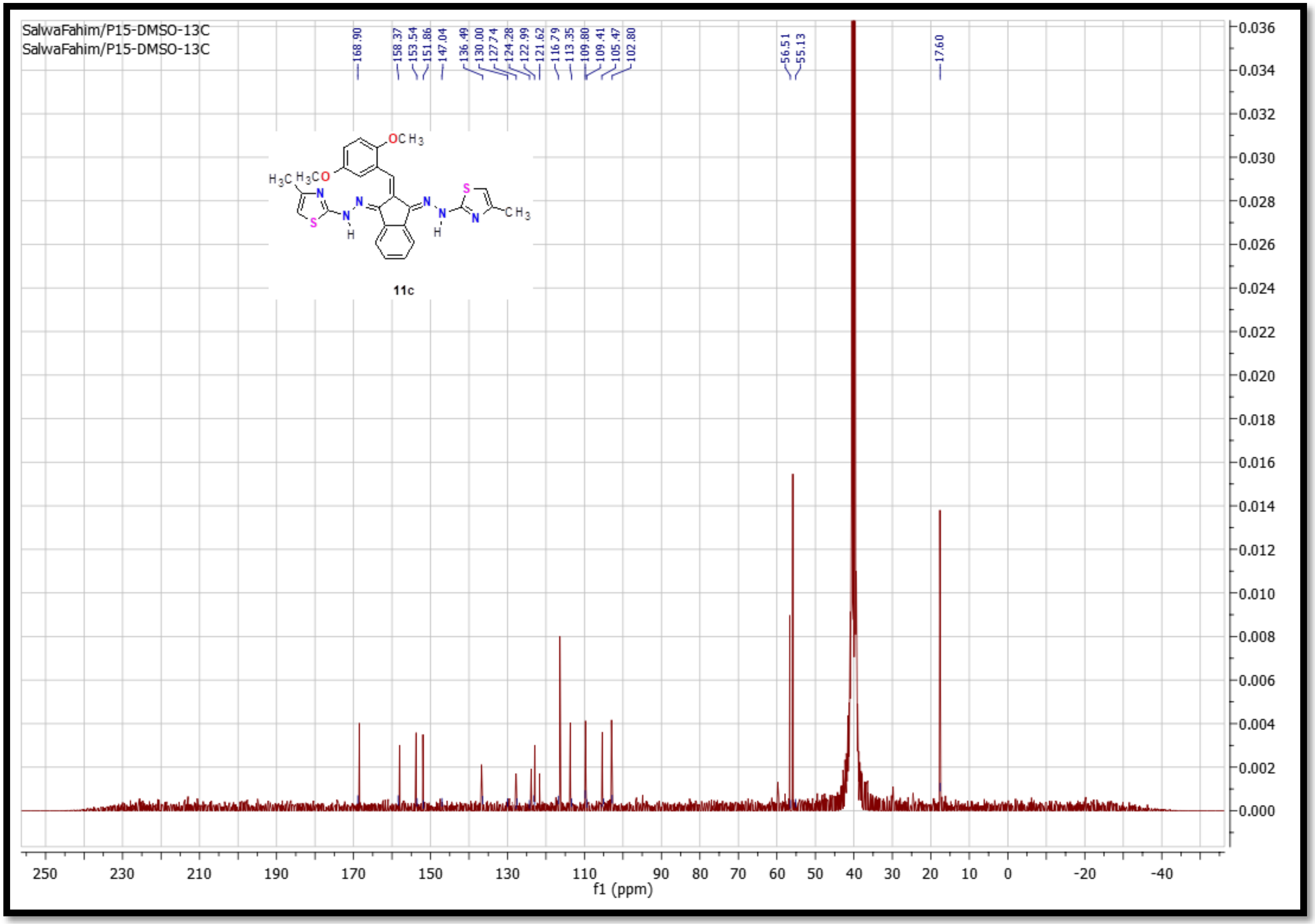

Figure S37. ^{13}C-NMR spectrum of compound (**11c**)

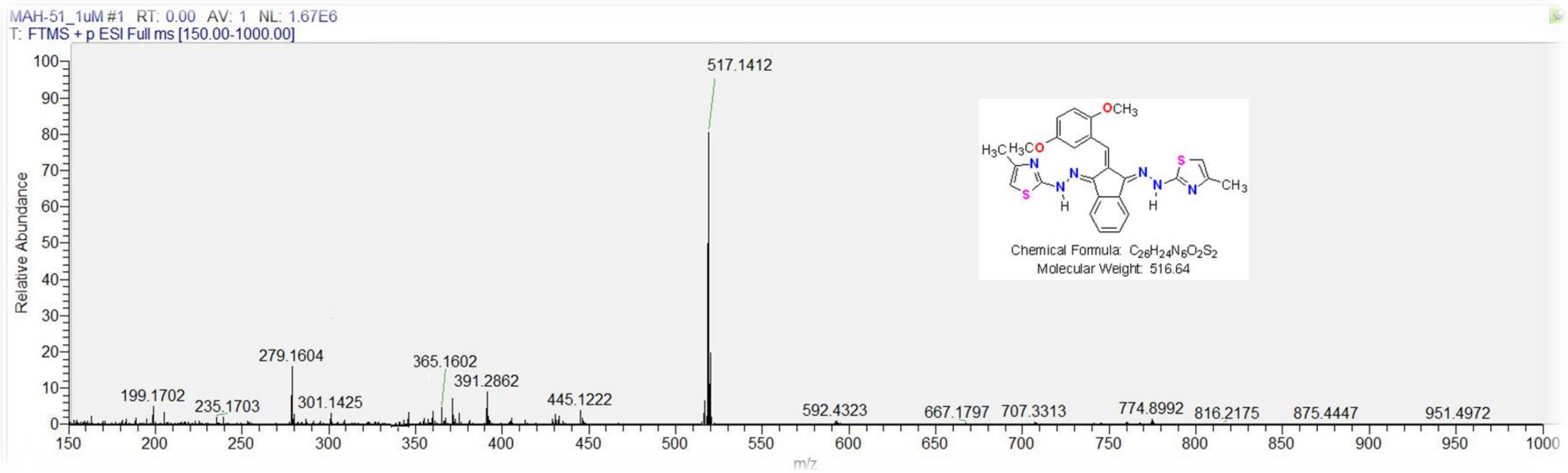

Figure S38. Mass spectrum of compound (**11c**)

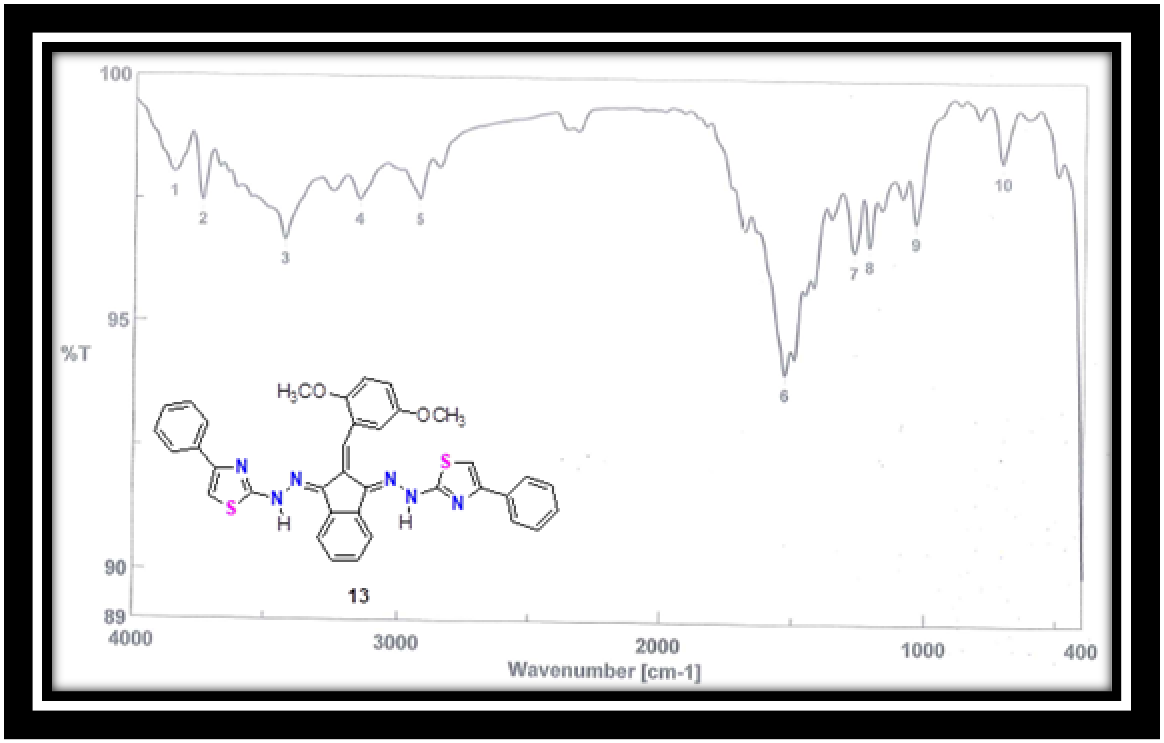

Figure S39. IR spectrum of compound (**13**)

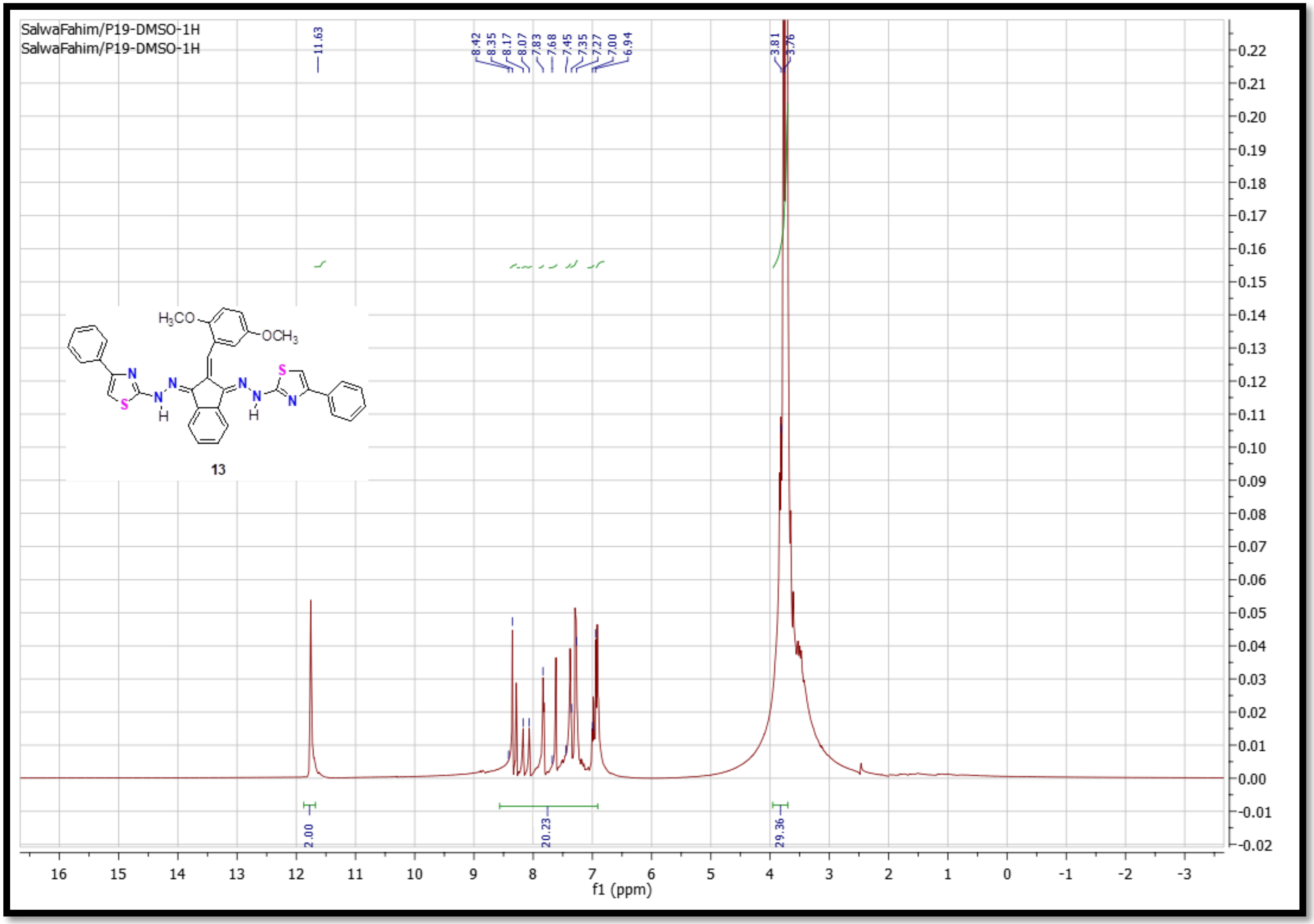

Figure S40. ^{1}H-NMR spectrum of compound (**13**)

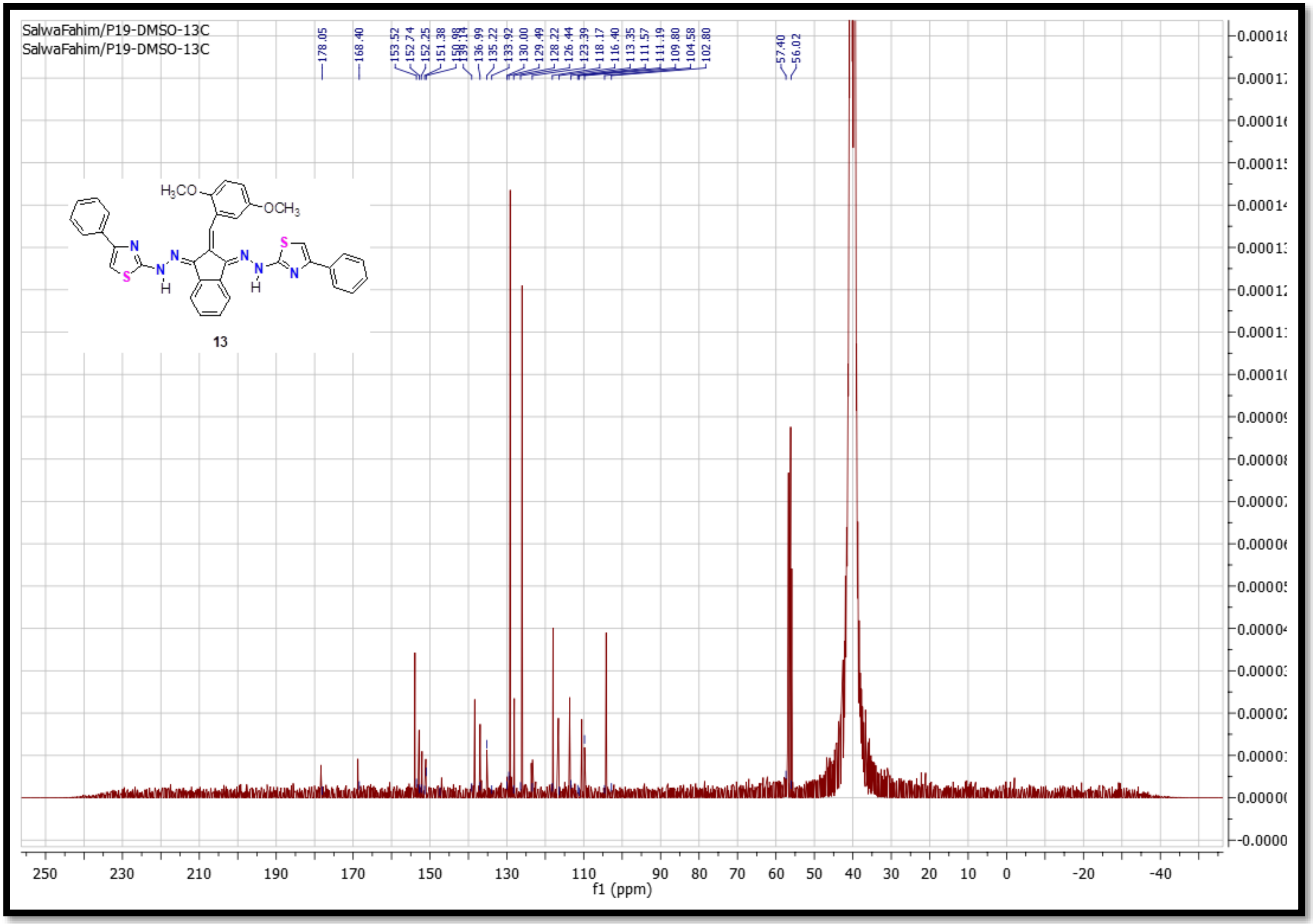

Figure S41. ^{13}C-NMR spectrum of compound (**13**)

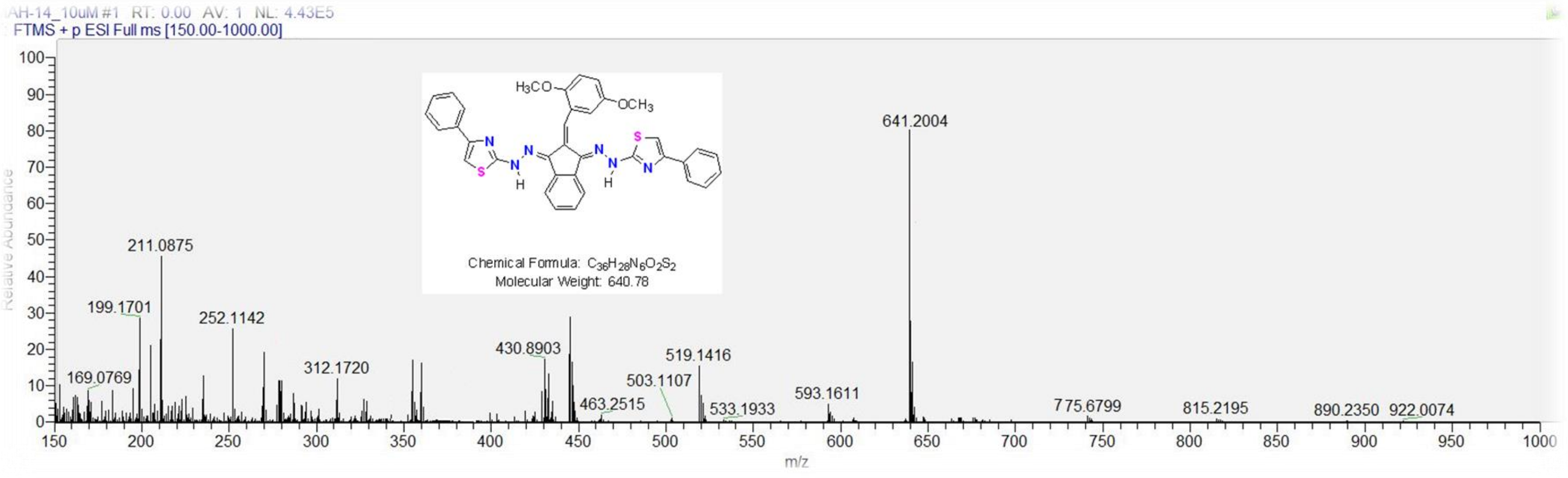

Figure S42. Mass spectrum of compound (**13**)
